# Steps of the Replication Cycle of the Viral Haemorrhagic Septicaemia Virus (VHSV) Affecting Its Virulence on Fish

**DOI:** 10.3390/ani10122264

**Published:** 2020-12-01

**Authors:** Carmen López-Vázquez, Isabel Bandín, Valentina Panzarin, Anna Toffan, Argelia Cuenca, Niels J. Olesen, Carlos P. Dopazo

**Affiliations:** 1 Instituto de Acuicultura-Dpt Microbiología, Universidade de Santiago de Compostela, 15782 Santiago de Compostela, Spain; mdelcarmen.lopez.vazquez@usc.es (C.L.-V.); isabel.bandin@usc.es (I.B.); 2 Department of Comparative Biomedical Sciences, Istituto Zooprofilattico Sperimentale delle Venezie, viale dell’Università 10, 35020 Legnaro, Padova, Italy; vpanzarin@izsvenezie.it (V.P.); atoffan@izsvenezie.it (A.T.); 3 Unit for Fish and Shellfish Diseases, National Institute of Aquatic Resources, Technical University of Denmark, Kemitorvet 202, 2800 Kgs Lyngby, Denmark; arcun@aqua.dtu.dk (A.C.); njol@aqua.dtu.dk (N.J.O.)

**Keywords:** novirhabdovirus, viral cycle, viral production, RNA synthesis, trout

## Abstract

**Simple Summary:**

Replication studies are frequently based on viral production, which provides limited information to understand certain processes. Therefore, to discover which failures in the viral haemorrhagic septicaemia virus (VHSV) replication cycle might be involved in the differences in its virulence on fish, a different approach has been taken. Our results have demonstrated that adsorption and morphogenesis are the steps most involved in the modulation of virulence, although failures in the synthesis step were also observed. As a potential application of our results, we believe that this kind of knowledge relating in vivo virulence to in vitro markers could help reduce the need for experimental infections in animals, representing a step forward in ethical issues.

**Abstract:**

The viral haemorrhagic septicaemia virus (VHSV), a single-stranded negative-sense RNA novirhabdovirus affecting a wide range of marine and freshwater fish species, is a main concern for European rainbow trout (*Oncorhynchus mykiss*) fish farmers. Its genome is constituted by six genes, codifying five structural and one nonstructural proteins. Many studies have been carried out to determine the participation of each gene in the VHSV virulence, most of them based on genome sequence analysis and/or reverse genetics to construct specific mutants and to evaluate their virulence phenotype. In the present study, we have used a different approach with a similar aim: hypothesizing that a failure in any step of the replication cycle can reduce the virulence in vivo, we studied in depth the in vitro replication of VHSV in different cell lines, using sets of strains from different origins, with high, low and moderate levels of virulence for fish. The results demonstrated that several steps in the viral replication cycle could affect VHSV virulence in fish, including adsorption, RNA synthesis and morphogenesis (including viral release). Notably, differences among strains in any step of the replication cycle were mostly strain-specific and reflected only in part the in vivo phenotype (high and low virulent). Our data, therefore, support the need for further studies aimed to construct completely avirulent VHSV recombinants targeting a combination of genes rather than a single one in order to study the mechanisms of genes interplay and their effect on viral phenotype in vitro and in vivo.

## 1. Introduction

The viral haemorrhagic septicaemia virus (VHSV) is a worldwide virus causing a widespread disease (VHS) in a large list of fish species, both from marine and freshwater environments [1]. The virus has been classified into four genotypes and several sublineages that reflect, at least in part, their geographical origin and host range [2,3,4]. Genogroup I includes subgroups I (constituted by the unclassified DK-F1 original VHSV strain [5]), Ia (constituted by freshwater European isolates), Ib (including mainly marine isolates from Northern Europe, although some strain has also being detected in Japan; [6]), Ic (constituted by isolates from continental Europe), Id (with marine and freshwater strains from Scandinavia and the Baltic Sea), and Ie (marine isolates from the Black Sea). Genogroups II and III include marine strains from the Baltic Sea and the North Atlantic Sea, respectively. Four subgroups constitute genogroup IV: IVa, which includes isolates from the Pacific coast of North America and from Japanese and Korean areas; IVb, represented by strains from the Great Lakes, and IVc, from the North America Atlantic coast, and IVd recently described from lumpfish in Iceland [7]. Among all these viral groups, isolates from cultured rainbow trout (*Oncorhynchus mykiss*) are mostly represented in genogroup I [8].

The VHSV is a ssRNA enveloped bullet-shaped virus within the family *Rhabdovididae*, genus *Novirhabdovirus*. Its genome, 11 to 12 Kb long, codifies six structural and nonstructural proteins (from 3′ to 5′). The N gene codifies the nucleoprotein, the major structural viral protein. The P gene corresponds to the phosphoprotein, which, as reported by Park et al. [9], is essential for the replication of VHSV since it forms part of the ribonucleoprotein, and acts as a bridge between the N-RNA complex and the polymerase. The M gene encodes the matrix protein, which condenses the nucleocapsid and has an essential role in favoring viral replication by suppressing cell transcription [10]. The G gene codifies the viral glycoprotein, implicated in cell specificity and antigenic activity. The NV gene encodes the homonym nonstructural protein, of significant importance for higher efficiency of the viral production in vitro and in vivo due to its involvement in suppressing cell apoptosis [11,12,13]. The L gene corresponds to the viral polymerase, responsible of the transcription and replication of the viral genome, and which has also been demonstrated to modulate virulence [14].

Many studies have been performed to understand the involvement of each gene in determining the level of virulence of VHSV to the host. Most are based on the analysis of genome sequences, either by assessing the nucleotide divergence among strains with different levels of virulence [15,16,17,18], by deleting or substituting complete genes [19,20,21], or by assessing the effect of specific mutations on virulence [10,14,20,22,23].

Those studies have led to the identification of viral genes implicated in the replication cycle, or in the understanding of the host response. However, whether and how VHSV virulence could be associated with failures in specific steps of the replication cycle have never been assessed.

This study was performed within Novimark, an Era Net-Anihwa EU project aimed to identify virulence markers to rainbow trout in novirhabdoviruses, with the participation of French, Italian, Danish, British and Spanish research teams. Previously, as part of the project, 55 VHSV strains from different origins were tested by the challenge in rainbow trout to determine their in vivo phenotype as described by percent cumulative mortality, and then subjected to high-throughput sequencing (HTS; using the Illumina MiSeq and IonProton^TM^ technologies) to identify amino acid polymorphisms putatively involved in virulence [24]. Afterward, candidate markers were validated through in vivo trials in rainbow trout, by assessing the effect of specific mutations on the virulence phenotype of recombinant viruses harboring specific amino acid signatures.

The objective of the present study was to evaluate the association of the level of virulence in vivo with the steps and kinetics of VHSV replication in vitro, in order to demonstrate when and how failures in replication modulates the level of virulence observed in vivo.

## 2. Materials and Methods

### 2.1. VHSV Strains

Fifteen VHSV strains provided by four different laboratories were tested (Table 1). The dataset encompasses viruses of different geographic origins, genotypes, and host species, as well as recombinant strains generated via reverse genetics. The selection of strains was based on their in vivo phenotype, whether it is field observation or percent cumulative mortality (CM) under experimental conditions as assessed by Panzarin et al. [24]. In their study, three levels of virulence were established: high [H] (CM > 42%), moderate [M] (14% < CM ≤ 42%) and low [L] (CM ≤ 14%).

Among the Italian collection strains, three of the highly virulent strains to rainbow trout were selected: two isolates from rainbow trout (TN68[H], TN80[H]; yielding 100% mortality) and one from brown trout (TN470[H], yielding 55.7% mortality). One additional strain from rainbow trout (TN480[L]) was selected due to its markedly lower level of mortality produced in vivo (14.5%). Regarding this last strain, although following the criteria indicated before it should be formally classified as a moderate [M] virulence strain. Based on our observations, it reflects a low virulent phenotype and thus was labeled as [L].

The Spanish strains were not tested against rainbow trout, and were selected for their virulence to its original host: SM2897[H], of high virulence to turbot [25], and DC1412[L], isolated from asymptomatic wild wedge sole (*Dicologlossa cuneata*).

Three Danish strains from the collection of the European Union Reference Laboratory (EURL) were tested, namely, DK-1p8[L] originally isolated from herring (*Clupea harengus*) and being avirulent to rainbow trout, and DK3592[H], of high virulence (92.3% mortality). Additionally, we also tested the F1 strain from the same host; because it caused variable percentages of mortality (1.3–17.3; i.e., between L and M), it was labeled as DK-F1[V] in the present study.

Finally, regarding the French strains only recombinant viruses were tested. Two recombinants (WT[H] and strain DD224[L]) constructed by Biacchesi et al. [30] and Baillon et al. [23] from the wild-type strains VHSV23/75 [29] and DD224 (from Merlangius merlangus) respectively, were used as reference of high and low virulence. In addition, four recombinants with specific mutations at the NV and/or the N genes were selected due to their high (NV-R116Y[H]; 64% mortality), moderate (NV-R116S[M]; 34%), and low (N-K46G[L], 0%; NV_N[L], 2%) virulence profiles.

### 2.2. Cell Lines and Viral Titration

Three cell lines were employed in this study: EPC (epithelium papulosum cyprinid), RTG-2 (rainbow trout gonad) and BF-2 (bluefin gill). EPC and BF-2 were grown at 20°C, and RTG-2 at 15 °C, and all the assays were performed at 15 °C using 80% (approximately) confluent monolayers. The cells were grown with minimum essential medium with Earle’s salts (EMEM, Sigma, Paisley, PA49RF, UK) supplemented with 10% foetal bovine serum (FBS, Lonza, Porriño 36410, Spain), 100 iu/mL penicillin and 100 µg/mL streptomycin (EMEM-10); before the replication assays, the medium was substituted by EMEM with 2% FBS and antibiotics (EMEM-2; pH 7.5). The cell lines originally employed by each laboratory for the propagation of their strains were selected to perform the assays in this study. Additionally, RTG-2 was also selected because it corresponds to the fish species their virulence was evaluated in. In the case of the Danish strains, we tested an additional cell line (BF-2) since it is also usually employed by that laboratory for the propagation of the virus. In the case of the French strains, we did not test RTG-2 since we were unable to satisfactorily propagate them in those cells.

The viral titrations were performed using the endpoint dilution method and with the same cell line adopted for the corresponding replication assay. Briefly: ten-fold dilutions of each virus were inoculated (by triplicate) in cell monolayers in 96-well plates with EMEM (2% FBS). The plates were incubated at 15 °C, a maximum of 10 days. Titers were determined by the Reed and Müench [31] procedure and given as TCID_50_/_mL_.

### 2.3. Experimental Design for the Study of Viral Replication in Cell Monolayers

The capacity of the virus to replicate in vitro was assessed by evaluating three steps of the replication cycle: adsorption, synthesis and final progeny production.

#### 2.3.1. Adsorption

To test the viral adsorption efficiency in each cell line, all the strains from each laboratory were simultaneously assayed. Prior to the assay, and in order to adjust all the viruses to the corresponding multiplicity of infection (MOI), the strains were titrated as reported above in the cell line they were originally propagated in. Adsorption assays were performed in 48-well plates with semi-confluent monolayers. After removing the culture medium, three wells per virus were covered with 0.5 mL of viral inoculum at the corresponding MOI (0.1 TCID_50_/cell unless specified). After the corresponding adsorption time (15, 30, 45 or 60 min; time is specified in the figures), the inoculum was completely removed and its volume measured; this fraction, called RI (‘remaining inoculum’), corresponding to the nonadsorbed virus (NAV), was stored at −20 °C until titration, for maximum 48 h. After three gentle washes with EMEM, the monolayers were covered with a measured volume of EMEM-2 (pH 6.5), and the reversible adsorbed virus (RAV) recovered by vigorous pipetting and kept at −20 °C until titration. The monolayers were covered with a measured volume of EMEM-2, pH 7.5, and lysed by freezing-thawing to determine the virus at time zero. Titration was performed by two methods: (*i)* the endpoint dilution titration method described in Section 2.2, to determine viral concentration as TCID_50_/_mL_, and (*ii)* determining the number of RNA copies per ml by the VHSV absolute quantification real time reverse transcription polymerase chain reaction (RT-qPCR) procedure reported by López-Vázquez et al. [32], and described in Section 2.3.4.

The titration of RI and RAV, and the measurement of working volumes allowed extrapolating the following data:

- total inoculated virus: TIV = titer of original inoculum × inoculum volume,
- non adsorbed virus: NAV = titer of RI × RI volume,
- total adsorbed virus: TAV = TIV–NAV,
- irreversible adsorbed virus: IAV = TAV–RAV,

These parameters let us evaluate the capacity of the virus to adsorb to the cell surface under three points of view:

- the apparent adsorption efficacy: AAE = TAV/TIV × 100,
- the real adsorption efficacy: RAE = IAV/TIV × 100,
- the efficiency of adsorption: EOA = IAV/TAV × 100.

For the sake of clarity: the term efficacy defines the ability to perform an action, while efficiency refers to the energy or time required to perform it. In this regard, the virus might be able to adsorb to a high percentage (high efficacy of adsorption), but is most of it reversibly adsorbed (low efficiency), the probability of the virus to enter the cell would be reduced. The AAE is the value normally used by researchers to assess adsorption. However, it does not consider the occurrence of failures in the conformational changes of the viral-cell protein complex that are required for successful adsorption and penetration; this information is provided by the estimation of RAE, which is associated to the efficiency of adsorption (EOA): The closest the EOA to 100%, the highest the equivalence between AAE and RAE.

#### 2.3.2. RNA Synthesis and Progeny Production Kinetics

Time-course experiments: To evaluate the synthesis and morphogenesis phases, 48-well plates with semi-confluent monolayers were employed in time-course experiments. For each time-point (from 0 to 204 h p.i.: every 2 h between 0 and 6 h p.i.; every 3 h between 9 and 18 h, and every 12 h between 24 and 204 h p.i.), three replicas were used. In most cases, the time-course experiments were performed for a maximum of 180 h p.i. (7.5 d). With the Italian strains in EPC, 4 d p.i. the monolayers were almost completely lysed; therefore, the assay was not continued. In some cases, the monolayers seemed too damaged after 120 h p.i. and therefore, the assay was ended. And finally, for some strains (those with slower kinetics) the time course was extended until 8.5 d (204 h) p.i. To begin these assays, after completely removing the culture medium from cells, viruses were inoculated at a MOI of approximately 0.1, and incubated for adsorption for 1 h at 15 °C. Then, the inoculum was completely removed (the volume measured, and stored at –20 °C until titration), and the wells were gently washed with EMEM-2 (pH 7.5) and covered with fresh EMEM-2. At each time point, the overlaying medium was completely removed, measured and stored at −20 °C until titration (to quantify extracellular virus); the monolayers were then gently washed and covered with fresh EMEM-2 to be lysed by freezing-thawing for quantification of the intracellular RNA copies.

For quantification of the extracellular viral progeny, the endpoint dilution method was used as previously described. To determine the number of intracellular and extracellular RNA genome copies, the RT-qPCR VHSV absolute quantification procedure described by López-Vázquez et al. [32] was employed, as briefly described below.

#### 2.3.3. Total and Maximum Progeny Production

Two additional data sets were determined, using the same experimental setting adopted for the estimation of replication and progeny production kinetics: The total viral production was quantified when cytopathic effect (CPE) was visualized; the maximum viral production was extrapolated from the time course experiments considering the maximum titer observed in each replication curve.

#### 2.3.4. Absolute Quantitation of RNA Copies by RT-qPCR

Extraction of total RNA was carried out using the Ezna Total RNA purification Kit (VWR) following the indications of the manufacturer. The extracted RNA was resuspended in 60 μL of diethyl pyrocarbonate (DEPC)-treated water and quantified by absorbance at 260 nm (A260) in a Nanodrop ND-100 spectrophotometer (Nanodrop, ThermoFisher Scientific Technologies, Wilmington, DE, USA).

The synthesis of cDNA from viral RNA was performed using Superscript IV RT (ThermoFisher Scientific, Vilnius, Lithuania): 9 μL of RNA were mixed with 2.5 μM of random primers and the mixture incubated at 95 °C for 5 min and then at 4 °C for at least 1 min. A reverse transcription mixture containing 10 U/μL of enzyme, 0.5 μM dNTPs and 5 mM DTT in 1× first strand buffer was then added, and the final mixture incubated at 25 °C for 10 min, followed by 20 min at 50 °C. The enzyme was finally inactivated at 80 °C for 10 min.

For the qPCR amplification, an iCycler iQ CFX96TM Real Time System (Bio Rad, Hercules, CA, USA) was employed using the iQ™ SYBR^®^ Green Supermix (BioRad, Singapore). The reaction conditions were set as stated by the manufacturer’s instructions. Briefly: initially, a 3 min activation/denaturation step at 95 °C is applied, and then the mixture subjected to 40 cycles of amplification (denaturation for 15 s at 95 °C, annealing, and extension for 30 s at 58 °C). After the PCR cycles, a melting curve was generated to confirm the specificity of the amplicons: the temperature was raised from 58 to 95 °C, and the fluorescence detected during 10 s after each 0.2 °C.

Serial dilutions of titrated crude virus were used as a standard for quantification.

### 2.4. Analysis of the Noncoding 5’-Terminal Region

In those cases when a failure in morphogenesis was suspected, and based on the knowledge that the 5’-end region is involved in controlling the assembly of other rhabdoviruses [33], the sequence differences between strains with and without those putative failures in morphogenesis were compared (by means of the software CLS Sequence Viewer_vs8.0, Qiagen Aarhus C, Denmark) using the whole viral sequences previously determined by HTS as part of the Novimark project [24,34].

### 2.5. Statistical Analysis

All data shown in the Results section correspond to the average value from three replicas. The only exception was the estimation of the total and maximum progeny production, examined in a single replica.

Statistical analysis was performed using the software GraphPad Prism vs. 6.0 (GraphPad Software, La Jolla, CA, USA). For the comparison of the adsorption percentages and the viral titers, a two-tailed impaired *t*-test was employed; *p* values ≤ 0.05 demonstrated significant differences. For the comparison of the replication curves, two statistical tests were applied: correlation and ANOVA. To assess if the kinetics of two curves vary together (correlate), the correlation coefficient (r) was determined (an r value equal to 1 was interpreted as the perfect correlation, and the closest to 1, the highest correlation between both curves); r value was considered significant with a *p* value ≤ 0.05. Two curves can correlate (can vary) while being different in their topology. Therefore, for testing differences between curves, two additional approaches were used. First, a Sidak multiple comparison two-way ANOVA tests was employed (differences considered significant only for values of *p* ≤ 0.01); however, since the procedure does not consider that differences below 1 Log10 in viral titer are not significant, this test gave apparently erroneous interpretations in too many cases. Therefore, another approach was applied: average differences among each time point between the two curves were also determined. Two curves were considered different only if no correlation was demonstrated between them (*p* > 0.05) and/or if they were demonstrated to be different by ANOVA (*p* ≤ 0.01) with average titer differences higher than 1 Log_10_.

## 3. Results

Before presenting the results of this study, it is important to remark that its objective was not to compare the replication fitness of all the strains in each cell line, but to determine the replication steps that could be affected in those strains with lower virulence than others of the same origin. The origin was considered not due to the geographical location they were isolated from, but because the influence of the adaptation of isolates to the specific cell line clone used by each laboratory. To this regard, most of the laboratories used EPC cells, except one which used BF-2; we used our own clone of both lines. Additionally, considering that the virulence of the strains was tested in rainbow trout, RTG-2 cells were also tested for reference. These are the reasons why these cells were used and why the results are analyzed between strains of different levels of virulence within each group of strains, not between strains of different groups. On the other hand, the first assays (with the Spanish and Italian strains) were performed in RTG-2 at different times of adsorption and different MOIs, in order to evaluate viral production. As it will be shown, the results let us to decide, for the remaining assays, the use of 30 min of adsorption and a MOI of 0.1 for the RNA synthesis and progeny production studies.

### 3.1. Viral Adsorption

**Figure 1:**
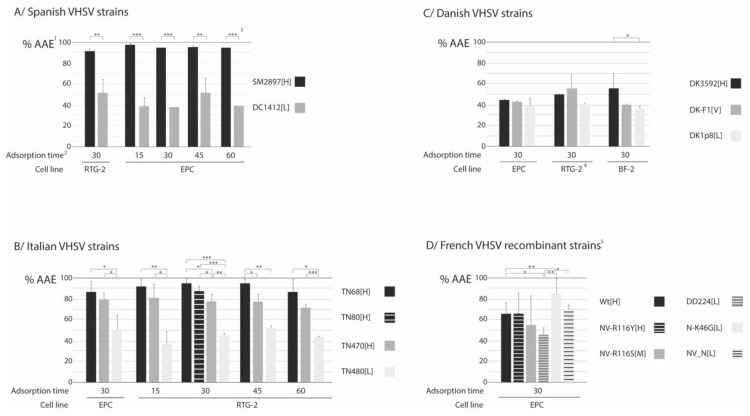
Efficacy of adsorption (using viral titer as *TCID_50_/*_mL_). ^1^ Due to the high efficiency of adsorption observed (see Supplementary Tables S1 to S4), it was possible to use only the apparent adsorption efficacy (AAE = adsorbed virus/inoculated virus ✕ 100). ^2^ Adsorption time of incubation shown in minutes. ^3^ A *t*-test was used to compare AAE values between [H] and [L] strains; significant differences between specific pair of strains within each group are shown: *, *p* ≤ 0.05; **, *p* ≤ 0.01; ***, *p* ≤ 0.001; for no significant differences, brackets are not shown (for the precise *P* values, see Supplementary Table S5). Error bars are shown for all the assays. ^4^ Data in RTG-2 are averaged from three repeats (3 replicas each). ^5^ All data (from three replicas each) with the French strains are averaged from one (NV-R116S[M], N-K46G[L] and NV_N[L]), 2 (NV-R116Y[H]) or three (the remaining strains) repeats.

The results obtained from the study of the adsorption capacity of the different VHSV strains tested are shown in Figure 1. The first important data was that the EOA (the efficiency of adsorption) was over 95% in most cases, considering quantification by viral titration, and over 90% in terms of RNA genome copies (see Supplementary Tables S1–S4). This made it possible to use just the AAE (apparent adsorption efficacy) values, which helps to simplify the analysis of results. Nevertheless, the three sets of data (AAE, RAE and EOA) are shown in Supplementary Tables S1 to S4, and the statistical analysis in Supplementary Table S5.

As shown, with the Spanish and Italian strains, the highest AAE values were obtained with the most virulent strains (SM2897[H], TN68[H], TN80[H], TN470[H]), with a difference of more than 40% (40–60%) in efficacy of adsorption with respect to those of lower virulence (DC1412[L], TN480[L]; *p* < 0.001, and from *p* < 0.05 to *p* < 0.001, respectively; Supplementary Table S5). Those differences were, only in a few cases, slightly lower when considering RNA genome copies (Supplementary Figure S1 and Supplementary Tables S1 and S2). With both sets of strains, a comparison between the percentages of adsorption observed at different times was performed using a *t* test, and no significant differences were observed, *P* values ranging from a minimum of 0.2217 (with the Spanish [L] strain, between 15 and 45 min) to 0.9682 (with the Italian TN68[H] strain, between 30 and 60 min) (Data not shown).

In the case of the Danish strains, either with viral titers (Figure 1C) or RNA genome copies (Supplementary Figure S1C), the AAE values were markedly lower than with the previous strains, and this finding was further confirmed by the use of a third cell line, BF-2. In addition, only in BF-2 the high virulent DK3592[H] strain showed a higher adsorption capacity than DK1p8[L] (*p* ≤ 0.05), and those differences were clearly higher in RNA genome copies (Supplementary Figure S1C). In RTG-2 and EPC, lower or no differences were observed between both strains (*p* > 0.05). Regarding F1[V], the highest adsorption in terms of TCID_50_ was observed in RTG-2 cells and in EPC considering RNA genome copies; interestingly, this strain showed variable results, being of similar, lower or higher adsorption values than DK3592[H], depending on the cell line.

With the French recombinants, although the strain used as reference of high virulence (WT[H]) showed higher adsorption capacity (average difference 22.04%; *p* < 0.05) than the one used as reference of low virulence (DD224[L]), the differences in AAE ranged from 12% (no significant: *p* = 0.0839; not shown) to more than 25% (significant: *p* = 0.0153; not shown) depending on the assay (Figure 1D and supplementary Table S5). More remarkable was the adsorption capacity of both avirulent recombinants, higher than that of the Wt[H] strain: N-K46G[L] (0% mortality on rainbow trout; Table 1) showed AAE 84.81% (more than 15% higher than the high virulence recombinant; *p* < 0,01), and NV_N[L] (0.2% mortality) showed AAE 73.61% (a little higher than Wt[H], although *p* > 0.05) (Supplementary Table S4).

### 3.2. Viral Production

**Figure 2:**
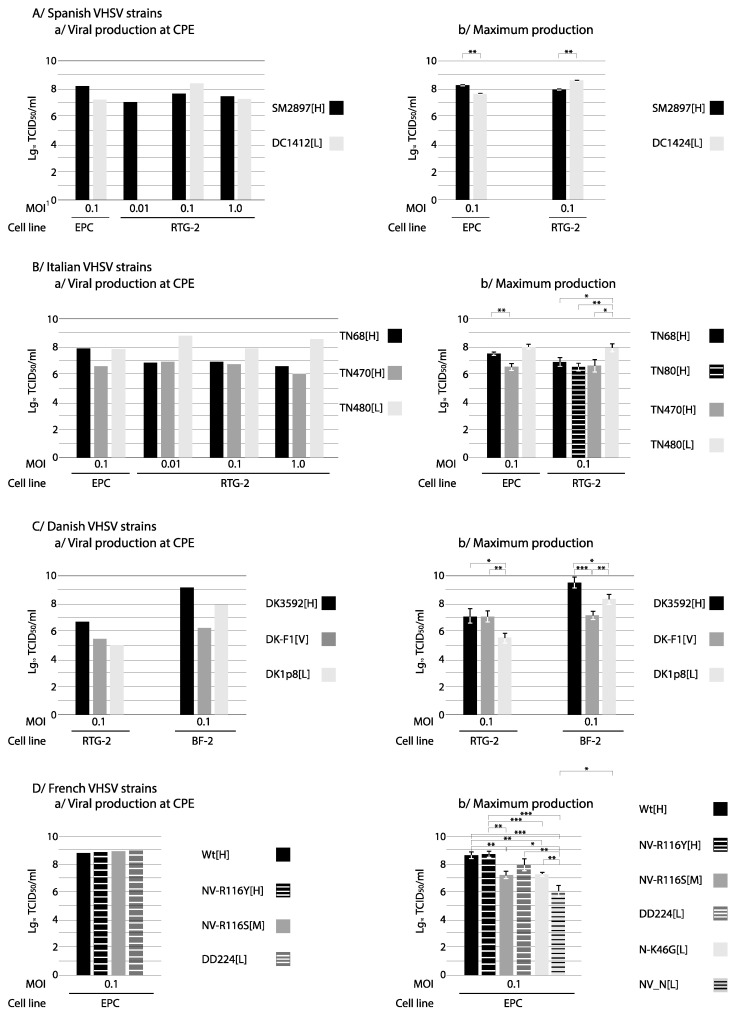
Viral production (using viral titer as TCID_50_/_mL_). Viral production was evaluated in two ways: (*i)* total viral production, by quantification of the crude virus when CPE was visualized, and (*ii)* maximum viral production, extrapolated from each curve considering the highest value observed. Data of total viral production are from a single assay; data of maximum production are from three replicas. Bars represent the standard deviation. MOI: multiplicity of infection (number of virus per cell) within each assay. A *t*-test was used to compare maximum production values between [H] and [L] strains; significant differences between specific pair of strains within each group are shown: *, *p* ≤ 0.05; **, *p* ≤ 0.01; ***, *p* ≤ 0.001; for no significant differences, brackets are not shown (for the precise *P* values, see Supplementary Table S8). When statistics could not be applied because no replicas were performed (viral production at CPE), the criterium that differences in titer higher that 1 Log_10_ can be considered significant was applied [35,36].

To analyze the total viral production, and to compare with the results of other authors, two sets of data were taken into account: the viral titer (and RNA genome copies) at the moment of development of CPE, and the maximum values observed along the replication cycle. The results using viral titer are shown in Figure 2 (and the numerical data in Supplementary Table S6, where time to development of CPE and to maximum viral production are also shown).

As shown in Figure 2A, among the Spanish viruses SM2897[H] was more productive in EPC and DC1412[L] in RTG-2, both at CPE and maximum production in terms of viral titer (at least at MOI 0.1); however, those differences did not exist (or were well below 1 Log_10_) when studying the number of RNA genome copies (Supplementary Figure S2 and Supplementary Table S7). It is noteworthy the effect of the MOI on the viral titer in RTG-2 (1.4 × 10^2^ TCID_50_/_mL_ with the [L] strain at MOI 0.01).

The interesting result with the Italian strains was the viral titer of TN480[L]; this strain of low virulence (close to the cut off value to [L] phenotype) showed the highest titer (with respect to TN68[H], TN80[H] and TN470[H]) at the moment of CPE production in RTG-2, and independently from the MOI. This pattern was not observed in EPC cells. The maximum viral production was also higher (and 2 d faster) with TN480[L] in both cells, EPC and RTG-2, but more remarkable in the latter (Figure 2B and Supplementary Table S6). Those differences were not observed in terms of RNA copies. We must remark that in EPC the monolayers appeared lysed 96 h p.i.; therefore, the maximum titers were recorded before that time. Considering that the peak of production in RTG-2 could appear later, higher maximum titer values in EPC cannot be rejected.

With the Danish strains, the results were the opposite: the virulent strain DK3592[H] was more productive (both in terms of viral titer at CPE and of maximum viral titer) than the low virulent DK-1p8[L] strain, in both RTG-2 and BF-2 cells (Figure 2C, and Supplementary Figure S2C). Again, the results with F1[V] were very variable depending on the measuring time and procedure, and on the cell line.

The French recombinant strains used as reference for high (WT[H]) and low (DD224[L]) virulence showed no difference in the viral production at CPE, and lower than 1 Log_10_ in the maximum titer (Figure 2D) (*p* > 0.05; see Supplementary Table S8). No differences between the high virulent Wt recombinant and NV-R116Y[H] were detected, but the maximum production of these 2 strains was higher with respect to N-K46G[L] (>1 Log_10_; *p* < 0.001), and much higher than NV_N[L] (>2 Log_10_; *p* = 0.001). Those differences where quite reduced in terms of RNA copies but NV_N[L] was also less productive in this case (Supplementary Figure S2 and Supplementary Table S7).

### 3.3. RNA Synthesis and Progeny Production

**Figure 3:**
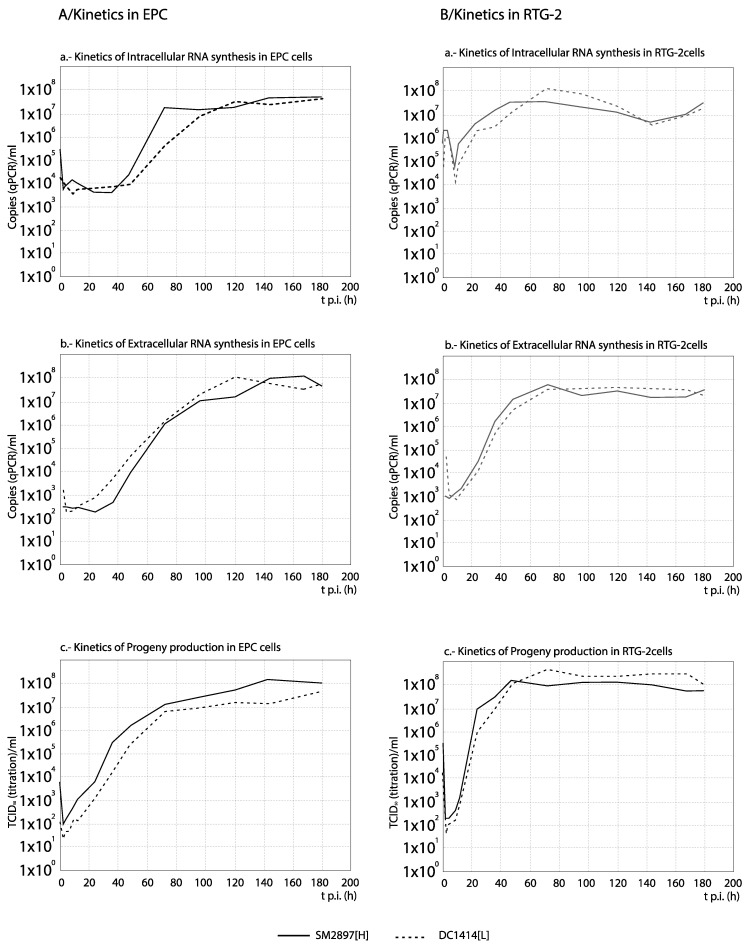
Comparison between the kinetics of RNA synthesis and progeny production among the Spanish strains. The kinetics of extracellular (**a**) and intracellular (**b**) RNA, and of progeny production (**c**) in EPC (**A**) and RTG-2 cells (**B**), are compared between both Spanish strains of high (SM2897[H]) and low (DC1414[L]) virulence *in vivo*. No significant differences between both strains were observed in any case: Significant correlation (high r values, with *p* ≤ 0.05) between curves and *p* values in ANOVA <0.05 with titers differences below 1 Log_10_ (see Supplementary Table S9). The results for each time point correspond to three replicas.

**Figure 4:**
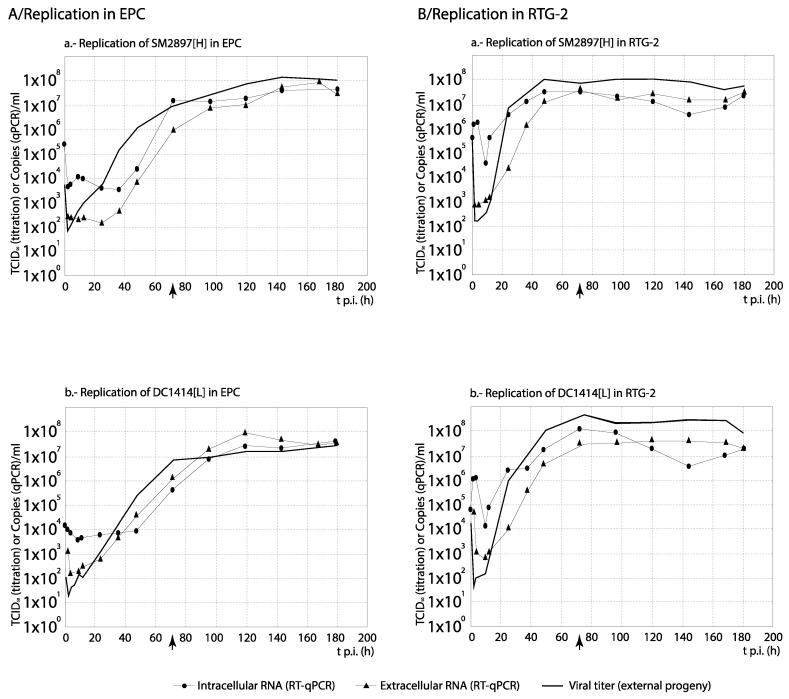
Analysis of the replication cycle of Spanish strains in cell lines. The kinetics of RNA synthesis and progeny production of each strain was evaluated in EPC (**A**) and RTG-2 (**B**) cells. The arrows show the time (hours p.i.) when CPE was visualized. For each time point, differences ≥1 Log_10_ between curves are considered significant. Each time point corresponds to three replicas.

**Figure 5:**
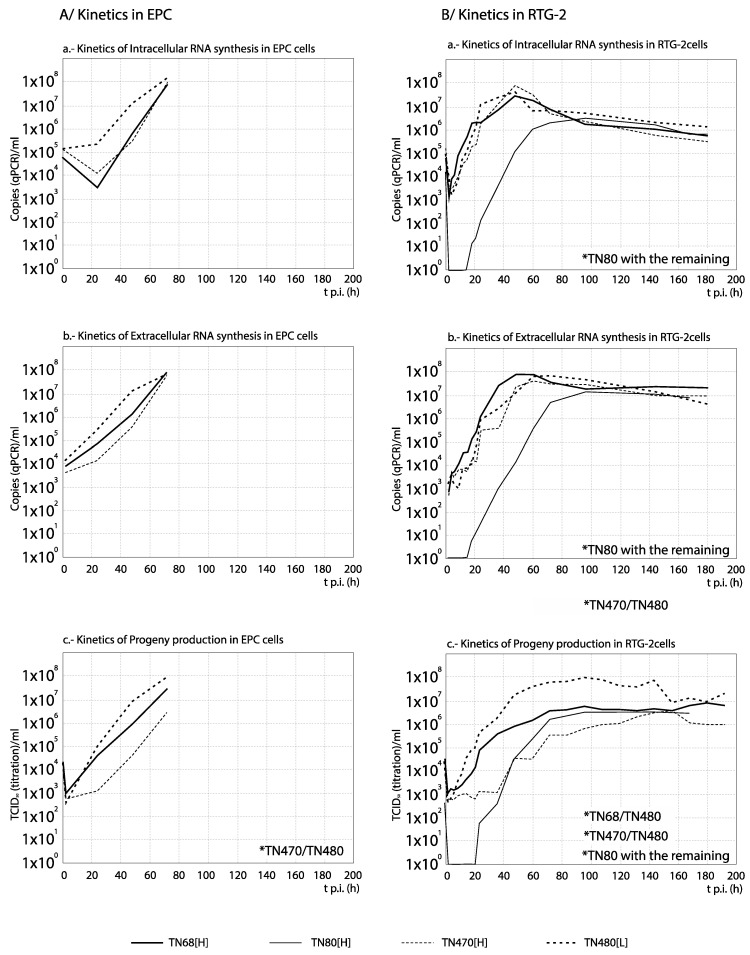
Comparison between the kinetics of RNA synthesis and progeny production among the Italian strains. The kinetics of extracellular (**a**) and intracellular (**b**) RNA, and of progeny production (**c**) in EPC (**A**) and RTG-2 (**B**) cells (**B**) are compared between the Italian strains. For statistically significant differences between each 2 curves, no correlation (r values with *p* > 0.05) and/or differences by ANOVA with *p* ≤ 0.01 associated with titer differences higher than 1 Log_10_ must be demonstrated (see Supplementary Table S10). * Pairs of curves with significant differences are indicated in the corresponding graph.

**Figure 6:**
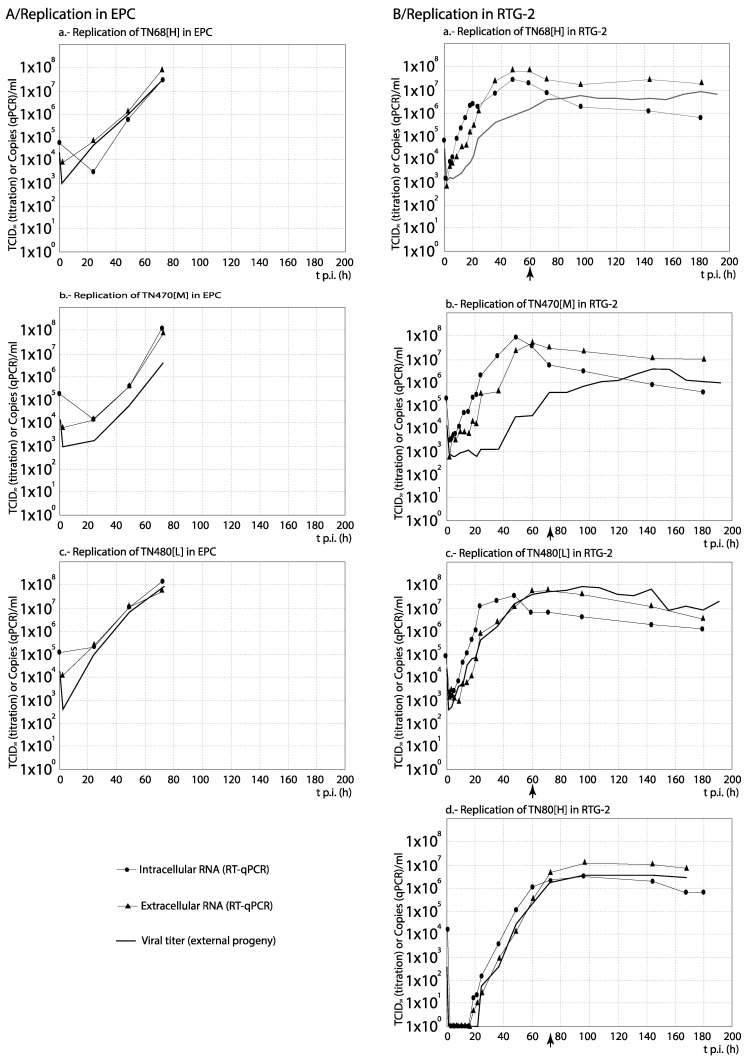
Analysis of the replication cycle of Italian strains in cell lines. The kinetics of RNA synthesis and progeny production of each strain was evaluated in EPC (**A**) and RTG-2 (**B**) cells. The arrows show the time (hours p.i.) when CPE was visualized. For each time point, differences ≥1 Log_10_ between curves are considered significant. Each time point corresponds to three replicas. Replication of TN80 was not assessed in EPC.

**Figure 7:**
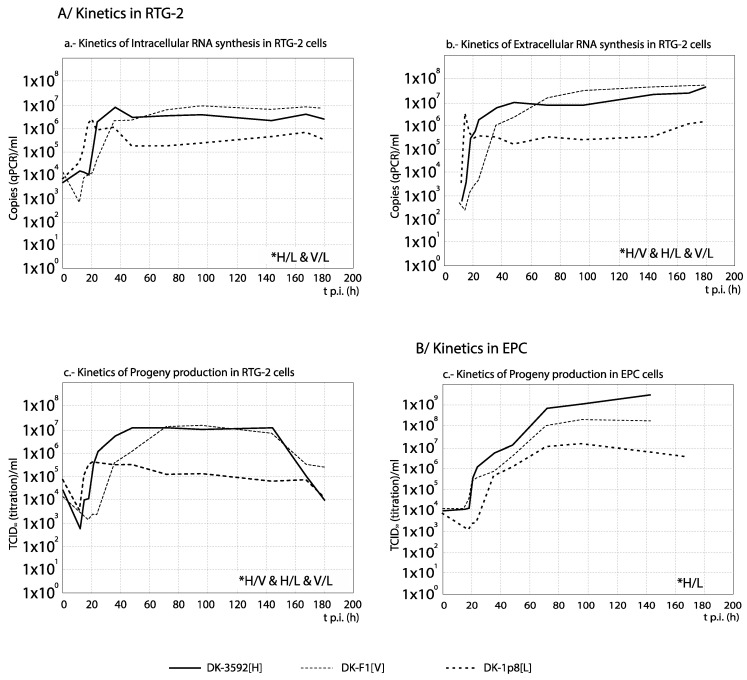
Comparison between the kinetics of RNA synthesis and progeny production among the Danish strains. The kinetics of extracellular (**a**) and intracellular (**b**) RNA in RTG-2, and progeny production (**c**) in EPC and RTG-2 cells are compared between three Danish VHSV strains with different levels of virulence in vivo. For statistically significant differences between each 2 curves, no correlation (r values with *p* > 0.05) and/or differences by ANOVA with *p* ≤ 0.01 associated with titer differences higher than 1 Log_10_ must be demonstrated (see Supplementary Table S11). * Pairs of curves with significant differences are indicated in the corresponding graph.

**Figure 8:**
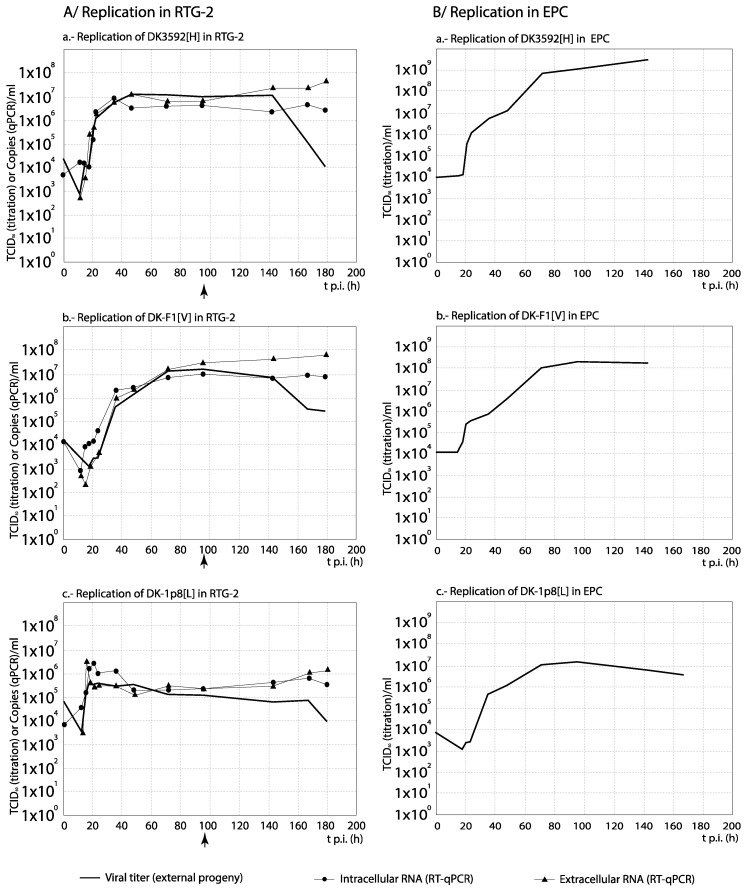
Analysis of the replication cycle of Danish strains in cell lines. The kinetics of RNA synthesis and progeny production of each strain was evaluated in EPC (**A**) and RTG-2 (**B**) cells. The arrows show the time (hours p.i.) when CPE was visualized. For each time point, differences ≥1 Log_10_ between curves are considered significant. Each time point corresponds to three replicas. In EPC, only data from progeny production was available.

**Figure 9:**
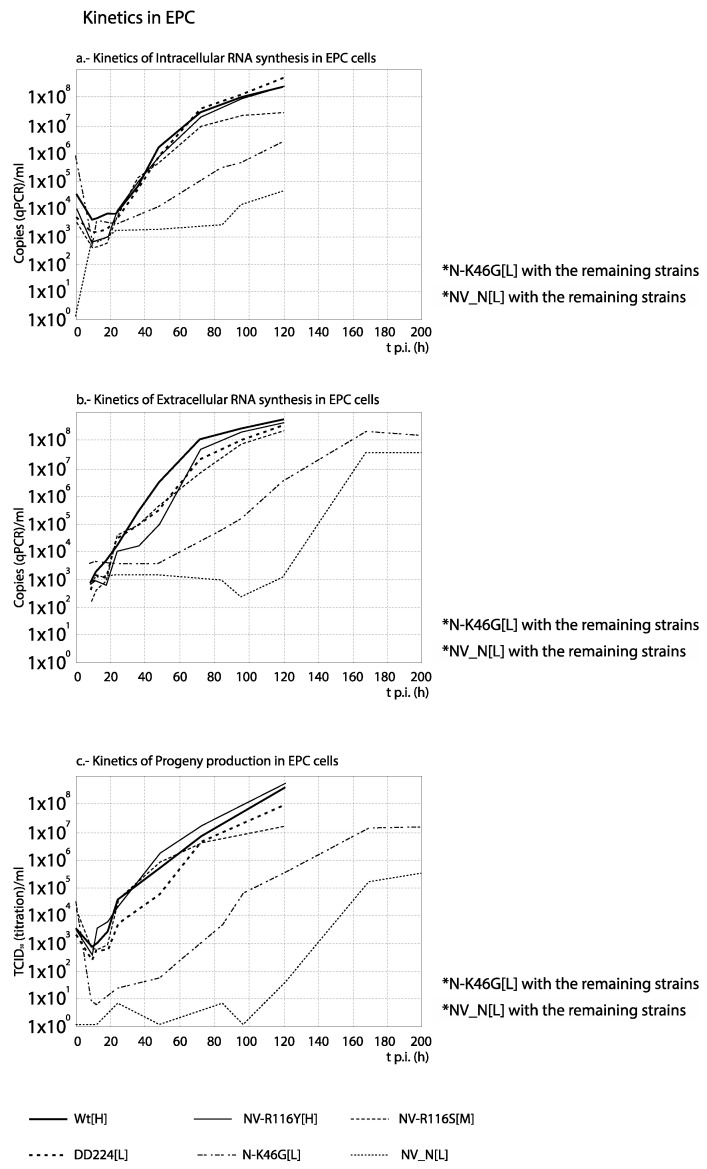
Comparison between the kinetics of RNA synthesis and progeny production among the French recombinant strains. The kinetics of intracellular (**a**) and extracellular (**b**) RNA and progeny production (**c**) in EPC cells are compared between several French VHSV strains and recombinants of different levels of virulence in rainbow trout. For statistically significant differences between every 2 curves, no correlation (r values with *p* > 0.05) and/or differences by ANOVA with *p* ≤ 0.01 associated with titer differences higher than 1 Log_10_ must be demonstrated (see Supplementary Table S11). * Pairs of curves with significant differences are indicated in the corresponding graph.

**Figure 10:**
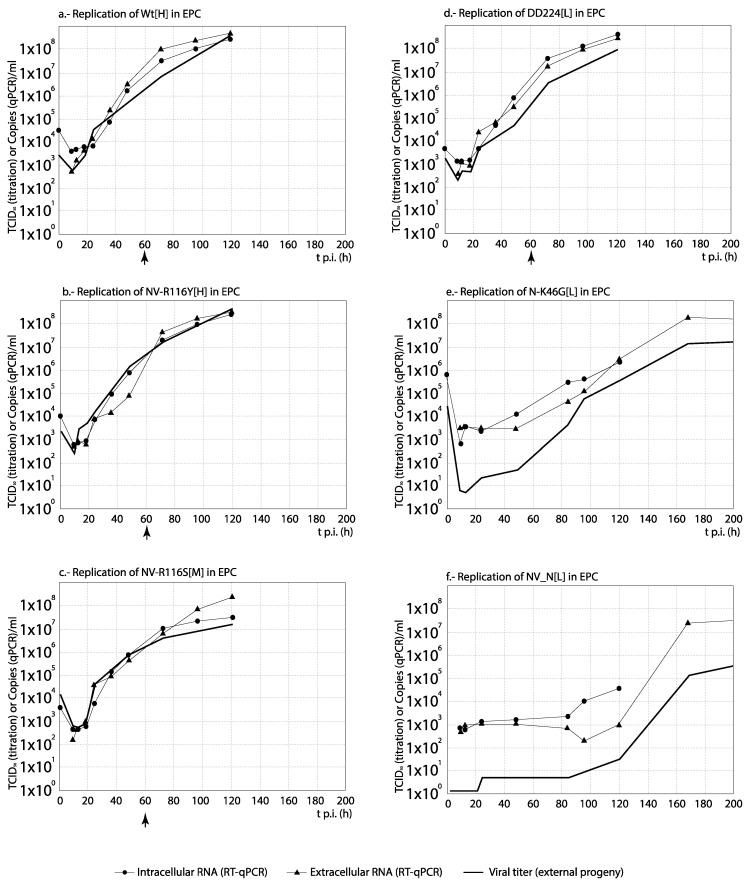
Analysis of the replication cycle of French recombinant strains in cell lines. The kinetics of RNA synthesis and progeny production of each strain was evaluated in EPC cells. The arrows show the time (hours p.i.) when CPE was visualized. For each time point, differences ≥1 Log_10_ between curves are considered significant. Each time point corresponds to three replicas.

For each set of strains, the curves obtained from the time course experiments are presented in two ways in order to assist the interpretation of the results: The comparison among [H] and [L] strains between their kinetics of RNA synthesis (intracellular and extracellular RNA) and progeny production is shown in Figures 3, 5, 7 and 9, whereas for each strain, its replication is evaluated in Figures 4, 6, 8 and 10 (the results from the statistical analysis are shown in Supplementary Tables S9 to S12). The first representation helps to detect differences between [H] and [L] strains within each group; the second helps to determine the affected steps in each specific strain.

Between both Spanish strains, no significant differences in RNA synthesis nor in progeny production (significant correlation between curves, and average differences in titer lower than 1 Log_10_) were observed throughout the replication study (except at specific time points) in neither the intracellular and extracellular fraction; such results were similar in both cell lines, EPC and RTG-2 (Figure 3). As expected, the progeny production (and the detection of extracellular viral RNA copies) was delayed with respect to the intracellular viral synthesis (Figure 4). We must note that, at least in part of the time course, the level of viral titers was higher (≥1 Log_10_) than that of the external RNA copies, a result which we cannot explain.

Among the Italian strains, and to facilitate the interpretation of results on synthesis kinetics, we will initially not include one of the high virulence strains: TN80[H]. Considering the remaining three strains, the results were different depending on the cell line: whereas in EPC the strain of lower virulence (TN480[L]) showed the highest synthesis kinetics, mainly of internal viral RNA (differences ≥1 Log_10_ were observed between 20 and 60 h p.i.; Figure 5A), in RTG-2 no differences were observed in intracellular and extracellular RNA copies (curves correlate, and average titer differences below 1 Log_10_; Supplementary Table S10). Only in terms of progeny, TN480[L] showed a markedly greater production than the [H] strains TN68[H] and TN470[H] after 2 h p.i. (Figure 5B) in this cell line. Interestingly, the level of RNA synthesis and progeny production of strain TN80[H] (only tested in RTG-2) was markedly delayed with respect to the other three strains, and only after 4 d p.i. the level of production matched between both [H] strains isolated from rainbow trout (Figure 5B). As shown in Figure 6 (and statistically demonstrated in Supplementary Table S10), no significant differences were observed between the kinetics of progeny production and viral RNA synthesis (mainly extracellular) of strains TN80[H] and TN480[L] in EPC and RTG-2 cells (significant correlation between curves (*p* ≤ 0.05), no significant differences by ANOVA (*p* > 0.01), and average titer differences below 1 Log_10_). In respect to the remaining two isolates (TN68[H] and TN470[H]), and mainly in RTG-2, the highest level of extracellular viral RNA than of viral titer (progeny) was observed (differences of more than 1 Log_10_ in most of the time course). We must remark that this result was not observed with any of the remaining strains except for two French recombinants, as it will be shown below.

Regarding the Danish strains, DK-3592[H] always showed a highest level of RNA synthesis and viral production, although slightly delayed a few hours at the beginning to the time course (Figure 7). Additionally, no differences between RNA synthesis and progeny production were observed in the replication of any of the three strains (Figure 8) (correlation between curves, and average titer differences ≤1 Log_10_; Supplementary Table S11).

As observed in Figure 9, with the French recombinant strains no important differences in RNA synthesis and progeny production were observed among both recombinants used as reference (WT[H] and (DD224[L]) and the mutant strains NV-R116Y[H] and NV-R116S[M] (correlation between curves, and average titer differences <1 Log_10_; Supplementary Table S12). On the contrary, the remaining two strains, N-K46G[L] and NV_N[L] (and more remarkably NV_N[L]), showed a significantly reduced and/or delayed RNA and progeny synthesis. Regarding the strains replication (Figure 10), again these two recombinants, and mainly NV_N[L], showed a progeny level quite reduced with respect to the quantity of external RNA (Figure 10e,f). However, much lower differences between RNA synthesis and progeny production were observed in the replication of any of the remaining four recombinants (Figure 10a–d). An interesting result was obtained with NV_N[L] in EPC, where this strain showed a significantly (>1 Log_10_) higher level of intracellular RNA than of extracellular RNA (between 84 and 120 h) and progeny titer (Figure 10f).

### 3.4. Non-Coding 5′-Terminal Region of the Italian Strains

**Figure 11:**
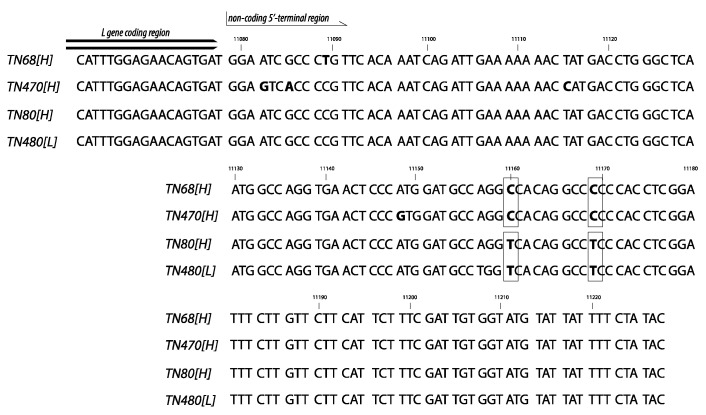
5′-terminal sequence of the Italian strains. Comparison of the Italian strains TN68[H], TN80[H], TN470[H] and TN480[L] noncoding 5′-terminal sequences (obtained as part of the NOVIMARK EraNet project [24,34]). Differences in nucleotides (nc) are labeled in bold; the nc differencing between TN68[H]/TN470[H] and TN80[H]/TN480[L] are highlighted by rectangles.

To try to find an explanation for the different behavior of the Italian strains TN68[H] and TN470[H] –with respect to the remaining strains– regarding the high rate between extracellular RNA copies and viral progeny, the noncoding 5′-terminal region of these strains was studied, using the sequences obtained by HTS as part of this project [24,34]. As shown in Figure 11, few differences between the 4 strains were observed in this region, and only at positions 11,160 and 11,169 (with respect to the strain TN68[H] sequence) those differences clearly differentiated TN68[H] and TN470[H] from TN80[H] and TN480[L], with cytosine or thymine in both positions, respectively. In most of the remaining strains (DK-1p8 and DK-F1, among the Danish strains and the strain DD224 and DK23-75 used to construct the French recombinants), thymine was observed at those positions (results not shown). Only in the case of the Danish strain DK3592, nucleotides C and T were present at those positions.

## 4. Discussion

The organization of the strains in groups by origin was casual: they were tested as they arrived to our laboratory from the reference laboratories. Our initial idea was to gather the results of all strains and analyze them together, assuming that there would be no differences between groups by origin. Surprisingly, there were differences linked to the strain origin: they did not behave similarly, on the contrary, the differences between [H] and [L] were due to different steps of the replication, depending on the laboratory of origin. That, of course, does not necessarily mean that strains from different origin behave differently in all cases, but, coincidentally, that was what happened in this study. It remains to be assessed whether the link between viral phenotype and laboratory of origin is related to the geographic provenance of the isolates, or to different fitness of the viruses in relation to the cell clones used at the different laboratories for viral propagation. Therefore, in order to focus to the mechanisms that explain differences between high and low virulence strains, we will maintain the organization of the discussion in groups of strains by their origin.

Few studies on the kinetics of VHSV replication in cell culture have been reported, and many of them with time intervals of 24 h, good enough to study the kinetics of viral replication, but not to analyze the synthesis phase, which is very important to understand the effectiveness of the viral cycle and the effect of any intrinsic or external factor on the virus. This is because the virus must need less than 18 h to complete a replication cycle, based on a very early report by Frost et al. [37], which set the stage for understanding the replication process of VHSV in cell lines (RTG-2 and FHM), from the viral adsorption to synthesis kinetics and progeny production. Inspired by this work, we have designed our experiments in order to assess the efficiency of VHSV replication at all the steps involved in this process, to identify the mechanisms possibly involved in VHSV virulence.

Based on the results of Frost et al. [37], a 1-h adsorption time was enough to reach a maximum 90% of adsorption to the cells. Those levels of adsorption have been reported in VHSV by other authors [38]. We have demonstrated that higher adsorption values can be obtained in as low as 15 min, but 30 min seems to be an appropriate adsorption time to ensure that even the low efficient strains reach their maximum adsorption capacity.

It is remarkable the high efficiency of adsorption (that is the EOA, or the percentage of irreversible adsorbed virus with respect to the total adsorbed) observed in most cases: higher than 95% (frequently over 99%). This means that the AAE and RAE values are similar. Only in two cases (two low virulent strains, DC1412[L] in EPC and DK1p8[L] in RTG-2) EOA values were around 90%; it would be interesting to study if any change in the glycoprotein in these strains could affect the conformational processes leading to internalization and making the adsorption step irreversible, but it was not the aim of our study. In addition, in their report, Frost et al. [37] obtained the highest viral titers at 18 h p.i., which does not coincide with what we have observed, probably because we have not worked with single-cycle replication assays.

In previous reports, other authors have studied the relationship between viral replication and sequence and its influence on viral replication. Ito et al. [39] reported the in vivo viral production of genotype III strains of different virulence level in rainbow trout, but their study did not provide data on the viral replication fitness. On the other hand, Cano et al. [40] analyzed the intracellular RNA synthesis of one [H] and four [L] strains, in comparison with the Mx expression, observing that there was an association between the higher intracellular RNA synthesis of [H] and the lower Mx expression of [L], even in coinfection. Unfortunately, the authors did not study the whole replication process to compare with our results. We agree that the high/low immune response is associated with [H]/[L] phenotypes, but the aim of our study was to determine which steps in the viral replication would be affected in the [L] strains, and this cannot be deduced just knowing that the [L] strains replication is affected. Therefore, we studied the intracellular and extracellular RNA synthesis and progeny production kinetics, in addition to the total and maximum viral production. Next, we will discuss the results obtained with each set of strains originating from the different laboratories.

In the case of the Spanish strains, no differences in the RNA synthesis and progeny kinetics that might explain their different levels of virulence were observed, nor differences in viral production (both, values at CPE and at the maximum of replication). The only significant difference was located at the adsorption step, since the highly virulent SM2897[H] strain showed percentages of adsorption much higher than the low virulence one (DC1412[L]). However, we must keep in mind that with these strains the level of virulence is not regarded to rainbow trout but to the original host species, namely turbot and wedge sole, respectively. This represents an important difference with the remaining sets of strains, since their level of virulence is referred to mortality in rainbow trout. It could be then interesting to evaluate the virulence of these two viruses to that host.

Also for the Italian strains, the highly virulent isolates showed a markedly higher adsorption capacity than the strain with the lowest virulence (TN480[L]). But, the impressive result was obtained in the RNA synthesis and viral production: RNA synthesis of TN480[L] strain equaled that of the high virulent one in RTG-2, and was much higher in EPC; in addition, the TN480[L] viral production significantly exceeded that of TN68[H]. This clearly indicates that the higher virulence of TN68[H] is not due to a higher replication capacity, but probably because of a higher capacity to spread through the cells due to its higher (and faster) adsorption capacity, although the time for the budding of the progeny, a step which was not studied here, could also be involved.

In a similar study by Park et al. [41], the authors reported that the difference in virulence between the U (low) and M (high) types of the infectious hematopoietic necrosis virus (IHNV), another novirhabdovirus, was related with a higher synthesis of viral RNA of the M type (37-fold higher than U) and of a higher viral production (42-fold). They demonstrated that the explanation was not in the construction of defective particles during the U strains replication, since the external RNA rate with respect to the M strain was similar (47-fold), and concluded that cell factors would be involved in more efficient control of the growth of the U-type strains than of the M type. Obviously, this is not the case with the Italian strains in our study, since the lower virulence strain is clearly more productive in cell culture. 

More interestingly, strains TN68[H] and TN470[H] showed a different replication pattern (with respect to the remaining two) in RTG-2 (and TN470 also in EPC): the level of progeny production was markedly low if related to the amount of extracellular RNA, suggesting either a failure in morphogenesis, producing a certain proportion of noninfective particles, or a failure in the release of infective particles, or a combination of both. In this regard, considering the statement by Whelan et al. [33] that in the vesicular stomatitis virus (VSV), another rhabdovirus, the noncoding 5′-end region controls the assembly of the virus, the 5′ termini of those two strains was compared with the remaining strains, finding two positions with different nucleotides. Would those differences be responsible for a failure in the morphogenesis/release? This is a question that further studies should answer. In Novimark, coding regions were prioritized as they were the objective of the design, but the search for virulence markers must be extended to noncoding regions, as well.

The Danish strains behaved in a different manner. First, their adsorption was clearly lower with respect to the Spanish and Italian strains, and the differences observed in AAE percentages between the [H] and [L] strains (except in BF-2 cells) were too low to justify the different levels of virulence. Regarding the fact that differences between the [H] and [L] strains were observed only in the absorption on BF-2, not on the other two strains, it is clear that the adsorption step (as all the replication cycle) is cell-dependent; therefore, the cell line where each strain has been propagated in could influence. However, all the strains had been propagated in the same cell line; thus, it would be risky to speculate, with the available data, the reason for that different behavior. On the other hand, both RNA synthesis and progeny production were clearly higher with the high virulence DK3592[H] strain than with DK-1p8[L]. Because this result is similar to that of Park et al. [41], it would be tempting to state that a higher efficiency of replication of the [H] strain would be the reason for its higher virulence. This would be supported by the results reported by Brudeseth et al. [42] testing in gill epithelial cells and macrophages the same two strains used in our study (DK3592[H] and DK1p8[L]). However, host factors such as a failure of the immune response should be ruled out to confirm, as Peñaranda et al. [43] already did for IHNV, that “a faster replication enables to rapidly achieve a threshold level of virus needed to override a strong host innate immune response”.

Regarding the French strains, the two recombinant strains used as a reference of high (WT[H]) and low (DD224[L]) virulence in rainbow trout showed no differences in RNA synthesis and progeny production kinetics, but the adsorption capacity of the [L] strain was markedly lower in some assays. Among the recombinants with specific mutations, NV-R116Y[H] and NV-R116S[M] were constructed by Baillon et al. [23] with single mutations in the NV gene, which provoked reduction of virulence in rainbow trout in both cases. The nonstructural protein codified by this gene has been widely studied and a consensus was reached regarding its role in the VHSV virulence in vivo, although certain controversy persists with respect to its expendability in viral replication. The NV protein downregulates the interferon response and suppresses apoptosis [12,13], which provides the virus with an advantage against its host, becoming more virulent than if the gene is not present. Regarding its involvement in replication, the suppression of apoptosis at early stages of the infection made Ammeyappan et al. [11] ensure that, although NV might not be essential for viral replication, its role in viral production is of a certain importance, which had already been stated for IHNV [44]. 

In our study, none of both mutants showed differences in the RNA-synthesis and progeny production kinetics, and in the viral titer at CPE, with respect to the [H] recombinant used as reference, what seems to suggest that the mutation does not affect the NV activity in vitro. However, the maximum production was clearly reduced in the NV-R116S[M] recombinant, which could be a clue to confirm that NV activity was actually affected and would explain the reduction of fish mortality reported by Baillon et al. [23]. Both recombinants (mainly NV-R116S[M]) also showed a reduced adsorption capacity with respect to the WT[H] reference strain, a result which we cannot explain since both share the same backbone sequence (except for the specific mutation of NV-R116S). Mutations at the aa positions 43 and 46 of the nucleoprotein had already been proposed to be potentially implicated in virulence [17,45], and mutations at those positions were applied to obtain the remaining French recombinants. 

The single mutation of N-K46G[L], located at the beginning of the N gene, and the combination of mutations in the N and NV genes applied to the NV_N[L] recombinant did not negatively affect their capacity of adsorption (the N-K46G[L] AAE was even higher than that of the WT[H] strain). However, their viral production at 5 d p.i (the last time point tested with the rest of French strain) was lowered 1–2 Log_10_ with respect to the [H] reference recombinant. Additionally, their kinetics of synthesis and progeny production were markedly delayed and reduced with respect to the rest of recombinants. Interestingly, these two strains showed a similar result, in terms of their replication pattern, than the Italian strains TN68[H] and TN470[H], since a lower level of progeny virus than extracellular RNA was observed. Since the backbone sequence of both N-K46G[L] and NV_N[L] strains is the DK23-75 strain, which did not show the same two mutations at the 5′-end than TN68[H] and TN470[H], the reason for the failure in morphogenesis cannot be the same. Instead, it might be that those mutations at the 3′-end of the N gene affect the nucleoprotein structure and, since N is the protein constructing the viral nucleocapsid, it is understandable that alterations in the N structure could affect assembly. But, in addition, since the N protein also captures the genome to—together with P and L proteins—form the ribonucleoprotein complex responsible for viral transcription and replication, it is also understandable that certain mutations in the N protein could also affect RNA synthesis. This would explain the results obtained with those two recombinants.

Summarizing our observations, it is obvious that, as expected, there is not a single mechanism affecting VHSV replication in vitro. Although our data must be further validated using animal models to fully translate our findings, we suggest that the modulation of VHSV virulence in vivo might also be regulated at different steps of the infectious process—even in a “cascade” manner. Indeed, mutations affecting adsorption reduce the ability of the virus to infect a single cell, but also the efficiency of the viral spread throughout the tissue, reducing its capacity to colonize the host. Similarly, mutations affecting the RNA synthesis (transcription and/or replication) and others affecting morphogenesis and/or viral release reduce the capacity of the virus to produce enough progeny and/or infect new cells. Such mutations, also have an indirect effect, as a lower capacity is the viral replication yields a more efficient host immune response against the infection, with the net result of a lower virulence phenotype. Therefore, strategies aimed to design completely voided virulence mutants should consider the need to target a combination of viral genes, rather than a single one; this will assist a better engineering of attenuated viruses to be used for the vaccine’s development. In addition, if a good set of in vitro phenotypic assay, coupled with molecular typing, was demonstrated to be sufficient to assess VHSV virulence, the use of experimental infections could be reduced.

## Figures and Tables

**Table 1:** Viral hemorrhagic septicemia virus strains tested.

Origin	Strain	Abbr Name ^1^	Gtyp ^2^	Origin ^3^	Virul ^4^	Reference ^5^
*Spanish*	VHSV/SpSM-2897.92	SM2897[H]	III	Turbot (Spain) *Scophthalmus maximus*	DT	López-Vázquez et al. [25,26]
	VHSV/SpDC-1412.08	DC1412[L]	III	Wedge sole (Spain) *Dicologlossa cuneata*	AF	Anonymous [27]
*Italian*	VHSV/ O. mykiss/I/TN/68/Feb15	TN68[H]	Ia	Rainbow trout (Italy) *Oncorhynchus mykiss*	100.0	N/R
	VHSV/O.mykiss/I/TN/80/Mar10	TN80[H]	Ia	Rainbow trout (Italy) *Oncorhynchus mykiss*	100.0	N/R
	VHSV/S.trutta/I/TN/470/Nov09	TN470[H]	Ia	Brown trout (Italy) *Salmo trutta*	55.7	N/R
	VHSV/O.mykiss/I/TN/480/Oct96	TN480[L]	Ia	Rainbow trout (Italy) *Oncorhynchus mykiss*	14.3	N/R
*Danish*	DK-3592B (Voldjerb)	DK-3592[H]	Ia	Rainbow trout (Denmark) *Oncorhynchus mykiss*	92.3	Mortensen et al. [28]
	DK-F1	DK-F1[V]	I	Rainbow trout (Denmark) *Oncorhynchus mykiss*	1.3–17.3	Jensen [5]
	DK-1p8	DK-1p8[L]	Ib	Herring (North Sea) *Clupea harengus*	0.0	N/R
*French*	*r*VHSV 23/75 ^6^	WT[H]	Ia	Brown trout (France) *Salmo trutta*	100.0	Thiery et al. [29] Biacchesi et al. [30]
	*r*VHSV DD224 ^7^	DD224[L]	III	Whitting (North Sea) *Merlangius merlangus*	0.0	N/R
	*r*VHSV NV-R116Y ^8^	NV-R116Y[H]	-		64.0	Baillon et al. [23]
	*r*VHSV NV-R116S ^8^	NV-R116S[M]	-		34.0	Baillon et al. [23]
	*r*VHSV N-K46G ^9^	N-K46G[L]	-		0.0	N/R
	*r*VHSV NV-R116S/N-K46G/N-G42R ^10^	NV_N[L]	-		2.0	N/R

^1^ Abbreviated name: In brackets: high (H), moderate (M), low (L) or variable (V) virulence on rainbow trout, as determined by Panzarin et al. [24]. ^2^ Gtyp: Genotype. ^3^ Origin: Common name (geographic origin)/*scientific name*. ^4^ Virulence: in the percentage of mortality of challenged rainbow trout (RT), as reported by Panzarin et al. [24]; the Spanish strains were not tested in that species, and their data are given as level of virulence in the original species (DT: Diseased turbot; AF: Asymptomatic fish). ^5^ Reference of the strain isolation-N/R: no reference available.^6^ A recombinant virus derived from the wild type VHSV23/75 [30], originally isolated from brown trout [28], and which demonstrated high virulence (98% of mortality) in RT [24]; the recombinant virus produced 100% mortality in RT. ^7^ A recombinant virus derived from the VHSV DD224 strain (Baillon/Biacchesi/Bremont, unpublished sequence). ^8^ Constructed from the recombinant VHSV 23/75, with a single aa change (R116Y or R116S) in the NV gene [23]. ^9^ Constructed from the recombinant VHSV 23/75, with a single aa change (K46G) in the N gene (Bremont/Biacchesi, unpublished). ^10^ Constructed from the recombinant VHSV 23/75, with a single aa change (R116S) in the NV gene, and 2 changes (K46G and G42R) in the N gene (Bremont/Biacchesi, unpublished).

## Supplementary Materials

The following are available online at https://www.mdpi.com/2076-2615/10/12/2264/s1, Figure S1: Efficacy of adsorption (using RNA copies determined by RT-qPCR, Figure S2: Viral production (using RNA copies determined by RT-qPCR), Table S1: Adsorption capacity of the Spanish VHSV strains, Table S2: Adsorption capacity of the Italian VHSV strains, Table S3: Adsorption capacity of the Danish VHSV strains, Table S4: Adsorption capacity of the French VHSV recombinant strains, Table S5: t Test comparison of adsorption values, Table S6: Viral production, quantified by viral titration, Table S7: Viral production, quantified by qPCR (RNA copies), Table S8: t Test comparison of maximum production values, Table S9: Correlation coefficient and statistical differences between replication curves: Spanish strains, Table S10: Correlation coefficient and statistical differences between replication curves: Italian strains, Table S11: Correlation coefficient and statistical differences between replication curves: Danish strains, Table S12: Correlation coefficient and statistical differences between replication curves: French strains.
