# Supplementary material for: Steps of the Replication Cycle of the Viral Haemorrhagic Septicaemia Virus (VHSV) Affecting Its Virulence on Fish
Source: Animals (Basel). 2020 Dec 1;10(12):2264. doi: 10.3390/ani10122264 (PMC7761041; doi:10.3390/ani10122264)
Supplement: Supplementary file 1 [file animals-10-02264-s001.zip › Supplementary items-wo Fig Legend-2/Supplementary Table 9-Corr coef and stat diffs between curves_Sp strains-vs3.docx]

Supplementary Table 9.- Correlation coefficient and statistical differences between replication curves: Spanish strains

| A.- Correlation between replication curves | | | | | | | | | | | | | | | | | | | | | | | | | | | | | | | | | | | | | | | | | | | | | | | | | | | | | | | | | | | | | | | | | | | | | | | | | | | | | |  |  |  |
| --- | --- | --- | --- | --- | --- | --- | --- | --- | --- | --- | --- | --- | --- | --- | --- | --- | --- | --- | --- | --- | --- | --- | --- | --- | --- | --- | --- | --- | --- | --- | --- | --- | --- | --- | --- | --- | --- | --- | --- | --- | --- | --- | --- | --- | --- | --- | --- | --- | --- | --- | --- | --- | --- | --- | --- | --- | --- | --- | --- | --- | --- | --- | --- | --- | --- | --- | --- | --- | --- | --- | --- | --- | --- | --- | --- | --- | --- | --- | --- | --- |
|  | |  | EPC | | | | | | | | | | | | | | | | | | | | | | | | | | | | |  |  | | |  | | | | | RTG-2 | | | | | | | | | | | | | | | | | | | | | | | | | | | | | | | | | | | | | |  |  |
|  | |  | Sm2897[H] | | | | | | | | | | | | | | | |  | | DC1412[L] | | | | | | | | | | |  | | |  | |  | | | | | Sm2897[H] | | | | | | | | | | | | | | | | | | |  | | DC1412[L] | | | | | | | | | | | | | | |  |  |  |
|  | |  | Intr | | | |  | | | Extr | |  | | | | Prog | | |  | | | Intr | |  | | Extr | |  | | Prog | |  | | |  | | |  | | | | | Intr | | |  | | | Extr | | |  | | | Prog | | |  | | | | Intr | | | |  | | | Extr | | |  | | | Prog | | | | | |
| Sm[H] | | Intr | - | | | |  | | | 0.983 | |  | | | | 0.860 | | |  | | | 0.945 | |  | | - | |  | | - | |  | | | Sm[H] | | | Intr | | | | | - | | |  | | | 0.814 | | |  | | | 0.792 | | |  | | | | 0.946 | | | |  | | | - | | |  | | | - | | | | | |
|  |  | Extr | - | | | |  | | | - | |  | | | | 0.915 | | |  | | | - | |  | | 0.978 | |  | | - | |  | | |  |  |  | Extr | | | | | - | | |  | | | - | | |  | | | 0.950 | | |  | | | | - | | | |  | | | 0.958 | | |  | | | - | | | | | |
|  |  | Prog | - | | | |  | | | - | |  | | | | - | | |  | | | - | |  | | - | |  | | 0.988 | |  | | |  |  |  | Prog | | | | | - | | |  | | | - | | |  | | | - | | |  | | | | - | | | |  | | | - | | |  | | | 0.984 | | | | | |
| DC[L] | | Intr | - | | | |  | | | - | |  | | | | - | | |  | | | - | |  | | 0.961 | |  | | 0.874 | |  | | | DC[L] | | | Intr | | | | | - | | |  | | | - | | |  | | | - | | |  | | | | - | | | |  | | | 0.861 | | |  | | | 0.810 | | | | | |
|  |  | Extr | - | | | |  | | | - | |  | | | | - | | |  | | | - | |  | | - | |  | | 0.568 | |  | | |  |  |  | Extr | | | | | - | | |  | | | - | | |  | | | - | | |  | | | | - | | | |  | | | - | | |  | | | 0.912 | | | | | |
|  |  | Prog | - | | | |  | | | - | |  | | | | - | | |  | | | - | |  | | - | |  | | - | |  | | |  |  |  | Prog | | | | | - | | |  | | | - | | |  | | | - | | |  | | | | - | | | |  | | | - | | |  | | | - | | | | | |
|  | |  |  | | | |  | | |  | |  | | | |  | | |  | | |  | |  | |  | |  | |  | |  | | |  | | |  | | | | |  | | |  | | |  | | |  | | |  | | |  | | | |  | | | |  | | |  | | |  | | |  | | | | | |
| B.- Differences between replication curves (2 ways ANOVA; data are shown as P values) | | | | | | | | | | | | | | | | | | | | | | | | | | | | | | | | | | | | | | | | | | | | | | | | | | | | | | | | | | | | | | | | | | | | | | | | | | | | | |  |  |  |
|  | |  | | | EPC | | | | | | | | | | | | | | | | | | | | | | | | | | |  | | |  | | | | |  | | | | | RTG-2 | | | | | | | | | | | | | | | | | | | | | | | | | | | | | | | | | | |  |
|  | |  | | | Sm2897[H] | | | | | | | | | | | | | |  | | | DC1412[L] | | | | | | | | | |  | | |  | | | | |  | | | | | Sm2897[H] | | | | | | | | | | | | | |  | | | | | DC1412[L] | | | | | | | | | | | | | | | |  |
|  | |  | | | Intr | | |  | | | Extr | | |  | | | Prog | |  | | | Intr | |  | | Extr | |  | | Prog | |  | | |  | | | | |  | | | | | Intr | | |  | | | Extr | |  | | | Prog | | |  | | | | | Intr | | |  | | | Extr | | |  | | | Extr | | | |  |
| Sm[H] | Intr | | | - | |  | | | 0.0002 | | | |  | | - | | |  | | 0.0026 | | |  | | - | |  | | - | |  | | | Sm[H] | | | | | Intr | | | | | - | | |  | | | <0.0001 | | | |  | | | - | | |  | | | | | <0.0001 | | |  | | | - | | |  | | | - | | | |
|  | Extr | | | - | |  | | | - | | | |  | | 0.0058 | | |  | | - | | |  | | <0.0001 | |  | | - | |  | | |  |  |  |  |  | Extr | | | | | - | | |  | | | - | | | |  | | | 0.0001 | | |  | | | | | - | | |  | | | 0.4277 | | |  | | | - | | | |
|  | Prog | | | - | |  | | | - | | | |  | | - | | |  | | - | | |  | | - | |  | | 0.0047 | |  | | |  |  |  |  |  | Prog | | | | | - | | |  | | | - | | | |  | | | - | | |  | | | | | - | | |  | | |  | | |  | | | <0.0001 | | | |
| DC[L] | Intr | | | - | |  | | | - | | | |  | | - | | |  | | - | | |  | | 0.9274 | |  | |  | |  | | | DC[L] | | | | | Intr | | | | | - | | |  | | | - | | | |  | | | - | | |  | | | | | - | | |  | | | <0.0001 | | |  | | | - | | | |
|  | Extr | | | - | |  | | | - | | | |  | | - | | |  | | - | | |  | | - | |  | | 0.0089 | |  | | |  |  |  |  |  | Extr | | | | | - | | |  | | | - | | | |  | | | - | | |  | | | | | - | | |  | | | - | | |  | | | 0.0004 | | | |
|  | Prog | | | - | |  | | | - | | | |  | | - | | |  | | - | | |  | | - | |  | | - | |  | | |  |  |  |  |  | Prog | | | | | - | | |  | | | - | | | |  | | | - | | |  | | | | | - | | |  | | | - | | |  | | | - | | | |

|  |  | C.- Differences between replication curves (Average difference between time points titers) | | | | | | | | | | | | | | | | | | | | | | | | | | | | | | | | | | | | | | |
| --- | --- | --- | --- | --- | --- | --- | --- | --- | --- | --- | --- | --- | --- | --- | --- | --- | --- | --- | --- | --- | --- | --- | --- | --- | --- | --- | --- | --- | --- | --- | --- | --- | --- | --- | --- | --- | --- | --- | --- | --- |
|  |  | EPC | | | | | | | | | | | | | | | |  |  | |  | | RTG-2 | | | | | | | | | | | | | | | | |  |
|  |  | Sm2897[H] | | | | | | |  | | DC1412[L] | | | | | | |  |  | |  | | Sm2897[H] | | | | | |  | DC1412[L] | | | | | | | | | |  |
|  | AvTD SD | Intr |  | Extr |  | | Prog |  | | Intr | |  | Extr |  | Prog |  |  | | | AvTD SD | | Intr | |  | Extr |  | Prog |  | Intr | | |  | | Extr | |  | | Extr |  |  |
| Sm[H] | Intr | - |  | 1.41* |  | 1.01* | |  | | 0.46 | |  | - |  | - |  | Sm[H] | | | Intr | | - | |  | 1.22* |  | 1.33* |  | 0.39 | |  | | - | |  | | - | |  |  |
|  | Extr | 2.07 |  | - |  | 1.48* | |  | | - | |  | 0.41 |  | - |  |  |  |  | Extr | | 1.20 | |  | - |  | 0.73 |  | - | |  | | 0.40 | |  | | - | |  |  |
|  | Prog | 0.70 |  | 2.13 |  | - | |  | | - | |  | - |  | 0.83 |  |  |  |  | Prog | | 1.33 | |  | 0.60 |  | - |  | - | |  | | - | |  | | 0.51 | |  |  |
| DC[L] | Intr | 0.45 |  | - |  | - | |  | | - | |  | 1.24* |  | 1.15* |  | DC[L] | | | Intr | | 0.30 | |  | - |  | - |  | - | |  | | 1.07* | |  | | 2.33* | |  |  |
|  | Extr | - |  | 0.33 |  | - | |  | | 1.93 | |  | - |  | 1.16* |  |  |  |  | Extr | | - | |  | 0.42 |  | - |  | 0.87 | |  | | - | |  | | 2.12* | |  |  |
|  | Prog | - |  | - |  | 0.45 | |  | | 0.87 | |  | 1.89 |  | - |  |  |  |  | Prog | | - | |  | - |  | 2.77 |  | 2.54 | |  | | 2.69 | |  | | - | |  |  |

A.- In the first part of the table, correlation between curves is given by the correlation coefficient values (**r**), being **r**=1 the maximum correlation between 2 curves; correlation is confirmed by P≤0.05; *no significant correlation values (**r** values with P>0.05). B.- In the second part of the table, the results of a SIDAK multiple comparison 2-way ANOVA test was employed (differences considered significant only for values of P≤0.01). C.- The third part shows the average differences of titer in each time point between two curves (average differences higher than 1 Log_10_ are considered significant and labelled with a *****). AvTD: Average titer differences (data in blue color; from 3 replicas); SD: Standard deviation.
