# Supplementary material for: Steps of the Replication Cycle of the Viral Haemorrhagic Septicaemia Virus (VHSV) Affecting Its Virulence on Fish
Source: Animals (Basel). 2020 Dec 1;10(12):2264. doi: 10.3390/ani10122264 (PMC7761041; doi:10.3390/ani10122264)
Supplement: Supplementary file 1 [file animals-10-02264-s001.zip › Supplementary items-wo Fig Legend-2/Supplementary Table 3-Adsorption Danish strains-vs6.docx]

Supplementary Table 3.- Adsorption capacity of the Danish VHSV strains

| *Cell line: EPC* |  |  |  |  |  |  |  |  |  |  |
| --- | --- | --- | --- | --- | --- | --- | --- | --- | --- | --- |
| Adsorption time |  | Strain |  | Method |  | AAE^1^ |  | RAE^2^ |  | EOA^3^ |
| 30 min |  | DK3592B[H] |  | TCID |  | 43.88±0.16 |  | 43.18±0.16 |  | 98.40±0.01 |
|  |  |  |  | qPCR |  | 44.50±14.34 |  | 42.69±14.84 |  | 95.94±3.70 |
|  |  | DK-F1[V] |  | TCID |  | 43.58±0.32 |  | 42.99±0.43 |  | 98.65±1.33 |
|  |  |  |  | qPCR |  | 60.15±18.82 |  | 58.96±20.30 |  | 98.02±4.70 |
|  |  | DK1p8[L] |  | TCID |  | 38.94±7.62 |  | 38.72±7.62 |  | 99.43±0.11 |
|  |  |  |  | qPCR |  | 37.14±10.61 |  | 34.62±10.00 |  | 93.22±3.84 |
| *Cell line: RTG-2 (Repeat 1)* | | |  |  |  |  |  |  |  |  |
| Adsorption time |  | Strain |  | Method |  | AAE |  | RAE |  | EOA |
| 30 min |  | DK3592B[H] |  | TCID |  | 43.18±0.46 |  | 40.75±2.68 |  | 94.37±5.78 |
|  |  |  |  | qPCR |  | 47.76±6.57 |  | 45.98±7.88 |  | 95.32±4.41 |
|  |  | DK-F1[V] |  | TCID |  | 53.55±13.98 |  | 52.38±14.20 |  | 97.81±1.29 |
|  |  |  |  | qPCR |  | 44.93±7.85 |  | 44.82±9.71 |  | 96.02±5.01 |
|  |  | DK1p8[L] |  | TCID |  | 43.77±6.49 |  | 39.45±7.91 |  | 90.13±10.14 |
|  |  |  |  | qPCR |  | 43.79±11.15 |  | 43.48±1.79 |  | 97.10±1.25 |
| *Cell line: RTG-2 (Repeat 2)* | | |  |  |  |  |  |  |  |  |
| Adsorption time |  | Strain |  | Method |  | AAE |  | RAE |  | EOA |
| 30 min |  | DK3592B[H] |  | TCID |  | 56.44±10.40 |  | 51.98±17.69 |  | 95.10±10.02 |
|  |  |  |  | qPCR |  | 47.62±6.57 |  | 45.88±7.78 |  | 95.32±4.41 |
|  |  | DK-F1[V] |  | TCID |  | 55.95±11.14 |  | 55.39±10.85 |  | 98.99±0.76 |
|  |  |  |  | qPCR |  | 46.67±7.55 |  | 43.52±8.50 |  | 96.02±5.01 |
|  |  | DK1p8[L] |  | TCID |  | 41.87±0.49 |  | 41.61±0.69 |  | 99.38±0.46 |
|  |  |  |  | qPCR |  | 22.77±13.33 |  | 19.66±12.19 |  | 89.99±1.15 |
| *Cell line: RTG-2 (Average^4^)* | | |  |  |  |  |  |  |  |  |
| Adsorption time |  | Strain |  | Method |  | AAE |  | RAE |  | EOA |
| 30 min |  | DK3592B[H] |  | TCID |  | 49.81±5.43 |  | 45.37±10.19 |  | 94.74±7.9 |
|  |  |  |  | qPCR |  | 47.76±5.88 |  | 45.93±7.83 |  | 95.32±4.41 |
|  |  | DK-F1[V] |  | TCID |  | 55.75±12.56 |  | 55.89±12.53 |  | 98.40±1.03 |
|  |  |  |  | qPCR |  | 44.93±7.02 |  | 44.17±9.15 |  | 96.02±5.01 |
|  |  | DK1p8[L] |  | TCID |  | 41.82±3.54 |  | 40.53±4.30 |  | 94.76±5.30 |
|  |  |  |  | qPCR |  | 33.28±15.91 |  | 31.57±6.99 |  | 93.55±1.20 |
| *Cell line: BF-2* |  |  |  |  |  |  |  |  |  |  |
| Adsorption time |  | Strain |  | Method |  | AAE |  | RAE |  | EOA |
| 30 min |  | DK3592B[H] |  | TCID |  | 55.77±15.28 |  | 53.88±15.85 |  | 96.61±1.96 |
|  |  |  |  | qPCR |  | 59.64±21.97 |  | 56.22±24.83 |  | 94.25±10.41 |
|  |  | DK-F1[V] |  | TCID |  | 40.95±0.56 |  | 39.91±1.50 |  | 97.45±2.59 |
|  |  |  |  | qPCR |  | 31.84±1.97 |  | 29.31±1.74 |  | 92.03±3.53 |
|  |  | DK1p8[L] |  | TCID |  | 35.89±2.98 |  | 35.01±2.89 |  | 97.54±0.81 |
|  |  |  |  | qPCR |  | 27.38±9.43 |  | 25.45±10.11 |  | 92.95±7.08 |

^1^Apparent adsorption efficacy: AAE=TAV (total adsorbed virus)/TIV (total inoculated virus) 🞪 100; ^2^Real adsorption efficacy: RAE=IAV (irreversibly adsorbed virus)/TIV 🞪 100; ^3^Efficiency of adsorption: EOA=IAV/TAV 🞪 100; ^4^Average from repeats 1 and 2.
