# Supplementary material for: Steps of the Replication Cycle of the Viral Haemorrhagic Septicaemia Virus (VHSV) Affecting Its Virulence on Fish
Source: Animals (Basel). 2020 Dec 1;10(12):2264. doi: 10.3390/ani10122264 (PMC7761041; doi:10.3390/ani10122264)
Supplement: Supplementary file 1 [file animals-10-02264-s001.zip › Supplementary items-wo Fig Legend-2/Supplementary Table 4-Adsorption French strains-vs3.docx]

Supplementary. Table 4.- Adsorption capacity of the French VHSV recombinant strains

| *Cell line: EPC* |  |  |  |  |  |  |  |  |  |  |  |
| --- | --- | --- | --- | --- | --- | --- | --- | --- | --- | --- | --- |
| Repetition 1: MOI 0.01-0.1 | | | | | | | | | | | |
| Adsorption time |  | Strain |  | Method |  | AAE^1^ |  | RAE^2^ |  | EOA^3^ |  |
| 30 min |  | Wt[H] |  | TCID |  | 52.02±11.68 |  | 51.72±11.56 |  | 99.41±0.10 |  |
|  |  |  |  | qPCR |  | 54.91±22.71 |  | 52.90±20.81 |  | 96.34±1.79 |  |
|  |  | DD224[L] |  | TCID |  | 39.28±4.05 |  | 37.98±3.22 |  | 96.69±1.68 |  |
|  |  |  |  | qPCR |  | 24.95±14.31 |  | 20.86±13.21 |  | 83.60±7.78 |  |
|  |  | NV-R116Y[H] |  | TCID |  | 50.15±11.98 |  | 48.90±12.39 |  | 97.51±2.11 |  |
|  |  |  |  | qPCR |  | 61.59±21.14 |  | 59.27±20.56 |  | 96.22±2.21 |  |
| Repetition 2: MOI 0.1-1 | | | | | | | | | | | |
| Adsorption time |  | Strain |  | Method |  | AAE^1^ |  | RAE^2^ |  | EOA^3^ |  |
| 30 min |  | Wt[H] |  | TCID |  | 72.99±7.99 |  | 72.60±7.99 |  | 99.46±0.06 |  |
|  |  |  |  | qPCR |  | 95.26±2.25 |  | 91.94±3.63 |  | 96.51±2.46 |  |
|  |  | DD224[L] |  | TCID |  | 50.27±24.79 |  | 49.77±24.92 |  | 99.01±0.99 |  |
|  |  |  |  | qPCR |  | 27.93±21.42 |  | 26.34±21.62 |  | 94.32±8.48 |  |
|  |  | NV-R116Y[H] |  | TCID |  | 70.99±28.50 |  | 70.89±28.54 |  | 99.82±.68 |  |
|  |  |  |  | qPCR |  | 79.07±7.11 |  | 77.55±7.44 |  | 98.07±0.61 |  |
|  |  | NV-R116S[M] |  | TCID |  | 55.07±19.57 |  | 54.97±19.62 |  | 99.83±0.14 |  |
|  |  |  |  | qPCR |  | 24.25±8.36 |  | 22.44±7.78 |  | 92.54±0.71 |  |
| Repetition 3: MOI 0.1-1 | | | | | | | | | | | |
| Adsorption time |  | Strain |  | Method |  | AAE^1^ |  | RAE^2^ |  | EOA^3^ |  |
| 30 min |  | Wt[H] |  | TCID |  | 74.67±13.07 |  | 74.37±13.03 |  | 99.59±0.08 |  |
|  |  |  |  | qPCR |  | 45.48±6.66 |  | 45.13±6.63 |  | 99.24±0.08 |  |
|  |  | DD224[L] |  | TCID |  | 43.99±0.23 |  | 42.21±1.68 |  | 95.96±4.22 |  |
|  |  |  |  | qPCR |  | 18.67±20.69 |  | 15.29±20.52 |  | 82.82±11.76 |  |
|  |  | NV_N[L] |  | TCID |  | 73.61±14.90 |  | 73.28±14.95 |  | 99.54±0.21 |  |
|  |  |  |  | qPCR |  | 50.94±9.49 |  | 49.21±9.39 |  | 96.61±2.51 |  |
|  |  | N-K46G[L] |  | TCID |  | 84.81±4.49 |  | 84.62±4.32 |  | 99.77±0.19 |  |
|  |  |  |  | qPCR |  | 49.52±23.24 |  | 46.57±22.84 |  | 94.06±3.73 |  |
| Average from 3 repeats | | | | | | | | | | | |
| Adsorption time |  | Strain |  | Method |  | AAE^1^ |  | RAE^2^ |  | EOA^3^ |  |
| 30 min |  | Wt[H] |  | TCID |  | 66.56±10.91 |  | 66.23±10.86 |  | 99.49±0.08 |  |
|  |  |  |  | qPCR |  | 65.22±10.54 |  | 63.32±10.36 |  | 97.36±1.44 |  |
|  |  | DD224[L] |  | TCID |  | 44.51±9.69 |  | 43.32±9.94 |  | 97.22±2.30 |  |
|  |  |  |  | qPCR |  | 23.85±18.81 |  | 20.83±18.85 |  | 86.91±9.34 |  |
|  |  | NV-R116Y[H] |  | TCID |  | 60.57±20.24 |  | 59.90±20.47 |  | 98.67±1.40 |  |
|  |  |  |  | qPCR |  | 70.33±14.13 |  | 68.41±14.00 |  | 97.15±1.41 |  |
|  |  | NV-R116S[M] |  | TCID |  | 55.07±19.57 |  | 54.97±19.62 |  | 99.83±0.14 |  |
|  |  |  |  | qPCR |  | 24.25±8.36 |  | 22.44±7.78 |  | 92.54±0.71 |  |
|  |  | NV_N[L] |  | TCID |  | 73.61±14.90 |  | 73.28±14.95 |  | 99.54±0.21 |  |
|  |  |  |  | qPCR |  | 50.94±9.49 |  | 49.21±9.39 |  | 96.61±2.51 |  |
|  |  | N-K46G[L] |  | TCID |  | 84.81±4.49 |  | 84.62±4.32 |  | 99.77±0.19 |  |
|  |  |  |  | qPCR |  | 49.52±23.24 |  | 46.57±22.84 |  | 94.06±3.73 |  |

^1^Apparent adsorption efficacy: AAE=TAV (total adsorbed virus)/TIV (total inoculated virus) 🞪 100; ^2^Real adsorption efficacy: RAE=IAV (irreversibly adsorbed virus)/TIV 🞪 100; ^3^Efficiency of adsorption: EOA=IAV/TAV 🞪 100.
