# Supplementary material for: Steps of the Replication Cycle of the Viral Haemorrhagic Septicaemia Virus (VHSV) Affecting Its Virulence on Fish
Source: Animals (Basel). 2020 Dec 1;10(12):2264. doi: 10.3390/ani10122264 (PMC7761041; doi:10.3390/ani10122264)
Supplement: Supplementary file 1 [file animals-10-02264-s001.zip › Supplementary items-wo Fig Legend-2/Supplementary Table 2-Adsorption Italian strains-vs2.docx]

Supplementary Table 2.- Adsorption capacity of the Italian VHSV strains

| *Cell line: EPC* |  |  |  |  |  |  |  |  |  |  |
| --- | --- | --- | --- | --- | --- | --- | --- | --- | --- | --- |
| Adsorption time |  | Strain |  | Method |  | AAE^1^ |  | RAE^2^ |  | EOA^3^ |
| 30 min |  | TN68[H] |  | TCID |  | 86.80±11.10 |  | 86.80±11.66 |  | 98.85±0.83 |
|  |  |  |  | qPCR |  | 79.23±15.88 |  | 77.97±15.92 |  | 98.41±0.82 |
|  |  | TN470[H] |  | TCID |  | 79.95±6.87 |  | 79.76±6.96 |  | 99.77±0.73 |
|  |  |  |  | qPCR |  | 66.59±17.88 |  | 66.22±18.13 |  | 99.45±0.73 |
|  |  | TN480[L] |  | TCID |  | 50.81±13.90 |  | 50.02±13.78 |  | 98.44±0.59 |
|  |  |  |  | qPCR |  | 57.28±6.34 |  | 56.27±6.44 |  | 98.22±0.60 |
|  |  |  |  |  |  |  |  |  |  |  |
| *Cell line: RTG-2* |  |  |  |  |  |  |  |  |  |  |
| Adsorption time |  | Strain |  | Method |  | AAE^1^ |  | RAE^2^ |  | EOA^3^ |
| 15 min |  | TN68[H] |  | TCID |  | 92.36±7.33 |  | 92.31±7.37 |  | 99.95±0.05 |
|  |  |  |  | qPCR |  | 83.57±6.68 |  | 81.56±7.92 |  | 97.59±2.59 |
|  |  | TN470[H] |  | TCID |  | 81.22±13.76 |  | 81.11±13.84 |  | 99.87±0.13 |
|  |  |  |  | qPCR |  | 78.98±10.48 |  | 78.01±10.03 |  | 98.78±0.45 |
|  |  | TN480[L] |  | TCID |  | 36.97±11.97 |  | 35.97±11.97 |  | 97.30±1.18 |
|  |  |  |  | qPCR |  | 43.75±16.55 |  | 42.90±16.72 |  | 98.06±1.90 |
| 30 min |  | TN68[H] | | TCID |  | 95.14±4.48 |  | 95.09±4.47 |  | 99.95±0.04 |
|  |  |  |  | qPCR |  | 88.81±8.82 |  | 88.10±9.14 |  | 99.19±0.64 |
|  |  | TN80[H] |  | TCID |  | 87.01±4.55 |  | 84.79±4.51 |  | 97.46±1.49 |
|  |  |  |  | qPCR |  | 88.56±14.22 |  | 77.66±22.36 |  | 87.69±18.55 |
|  |  | TN470[H] |  | TCID |  | 77.26±6.87 |  | 77.17±6.91 |  | 99.88±0.10 |
|  |  |  |  | qPCR |  | 75.24±16.13 |  | 74.54±15.94 |  | 99.07±0.44 |
|  |  | TN480[L] |  | TCID |  | 45.27±1.72 |  | 44.41±1.54 |  | 98.11±0.52 |
|  |  |  |  | qPCR |  | 36.40±4.94 |  | 35.77±4.79 |  | 98.26±0.94 |
| 45 min |  | TN68[H] | | TCID |  | 95.27±4.18 |  | 95.17±4.12 |  | 99.90±0.15 |
|  |  |  |  | qPCR |  | 89.86±4.53 |  | 88.58±4.94 |  | 98.58±0.91 |
|  |  | TN470[H] |  | TCID |  | 77.37±7.03 |  | 77.28±7.07 |  | 99.88±0.10 |
|  |  |  |  | qPCR |  | 77.39±16.32 |  | 76.65±16.64 |  | 99.05±0.87 |
|  |  | TN480[L] |  | TCID |  | 52.50±15.12 |  | 51.79±14.87 |  | 98.65±0.08 |
|  |  |  |  | qPCR |  | 48.06±18.09 |  | 47.36±18.73 |  | 98.55±3.12 |
| 60 min |  | TN68[H] | | TCID |  | 86.80±20.25 |  | 85.80±21.33 |  | 98.85±2.03 |
|  |  |  |  | qPCR |  | 87.42±8.09 |  | 86.32±8.44 |  | 98.74±0.59 |
|  |  | TN470[H] |  | TCID |  | 71.38±3.54 |  | 71.17±3.69 |  | 99.71±0.37 |
|  |  |  |  | qPCR |  | 72.00±13.87 |  | 71.36±14.37 |  | 99.11±0.93 |
|  |  | TN480[L] |  | TCID |  | 44.14±0.80 |  | 43.43±0.81 |  | 98.40±0.59 |
|  |  |  |  | qPCR |  | 40.31±19.12 |  | 39.53±19.07 |  | 98.06±1.30 |

^1^Apparent adsorption efficacy: AAE=TAV (total adsorbed virus)/TIV (total inoculated virus) 🞪 100; ^2^Real adsorption efficacy: RAE=IAV (irreversibly adsorbed virus)/TIV 🞪 100; ^3^Efficiency of adsorption: EOA=IAV/TAV 🞪 100.
