# Supplementary material for: Steps of the Replication Cycle of the Viral Haemorrhagic Septicaemia Virus (VHSV) Affecting Its Virulence on Fish
Source: Animals (Basel). 2020 Dec 1;10(12):2264. doi: 10.3390/ani10122264 (PMC7761041; doi:10.3390/ani10122264)
Supplement: Supplementary file 1 [file animals-10-02264-s001.zip › Supplementary items-wo Fig Legend-2/Supplementary Table 6-Viral production_TCID-vs3.pdf]

Supplementary Table 6- Viral production, quantified by viral titration

Spanish VHSV strains									
At the time of CPE[1]					Maximum viral production				
Line[2]	MOI[3]	Strain	Titer[4]	Time	Line	MOI	Strain	Titer	Time
EPC	0.1	Sm2897[H]	1.5×10^8	3d	EPC	0.1	Sm2897[H]	1.6×10^8 *8.18±0.15*	6d
		DC1412[L]	1.5×10^7	3d			DC1412[L]	4.2×10^7 *7.62±0.06*	7d
RTG-2	0.01	Sm2897[H]	1.1×10^7	4-5d	RTG-2	0.1	Sm2897[H]	8.8×10^7 *7.93±0.13*	6d
		DC1412[L]	ND				DC1412[L]	4.5×10^8 *8.63±0.14*	3d
	0.1	Sm2897[H]	4.1×10^7	3d					
		DC1412[L]	2.1×10^8	3d					
	1.0	Sm2897[H]	2.7×10^7	2-3d					
		DC1412[L]	1.8×10^7	3d					

Italian VHSV strains									
At the time of CPE					Maximum viral production				
Line	MOI	Strain	Titer	Time	Line	MOI	Strain	Titer	Time
EPC	0.1	TN68[H]	7.2×10^7	N/Av	EPC	0.1	TN68[H]	3.0×10^7 *7.47±0.14*	3d
		TN80[H]	NA				TN80[H]	NA	
		TN470[H]	3.5×10^7	N/Av			TN470[H]	4.0×10^6 *6.55±0.25*	3d
		TN480[L]	7.4×10^7	N/Av			TN480[L]	9.6×10^7 *7.97±0.14*	3d
RTG-2	0.01	TN68[H]	6.5×10^6	3-4d	RTG-2	0.1	TN68[H]	8.7×10^6 *6.88±0.29*	7d
		TN80[H]	NA				TN80[H]	3.5×10^6 *6.50±0.25*	6d
		TN470[H]	8.0×10^6	3-4d			TN470[H]	5.4×10^6 *6.58±0.45*	6.5d
		TN480[L]	6.2×10^8	3-4d			TN480[L]	8.7×10^7 *7.88±0.29*	4.5d
	0.1	TN68[H]	7.4×10^6	2-3d					
		TN80[H]	NA	3d					
		TN470[H]	5.0×10^6	2-3d					
		TN480[L]	6.5×10^7	2-3d					
	1.0	TN68[H]	3.5×10^6	2d					
		TN80[H]	NA						
		TN470[H]	1.1×10^6	2-3d					
		TN480[L]	3.1×10^8	2d					

Danish VHSV strains

At the time of CPE					Maximum viral production				
Line	MOI	Strain	Titer	Time	Line	MOI	Strain	Titer	Time
RTG-2	0.1	DK3592[H]	8.2 x 10^{6}<br>*6.75±0.53*	2-3d	RTG-2	0.1	DK3592[H]	1.7 x 10^{7}<br>*7.08±0.50*	2-3d
		DK-F1[V]	3.0 x 10^{5}<br>*5.47±0.14*	2-3d			DK-F1[V]	1.6 x 10^{7}<br>*7.08±0.38*	2d
		DK-1p8[L]	1.1 x 10^{5}<br>*5.05±0.10*	4d			DK-1p8[L]	3.8 x 10^{5}<br>*5.50±0.33*	2d
BF-2	0.1	DK3592[H]	1.8 x 10^{9}<br>*9.17±0.38*	3d	BF-2	0.1	DK3592[H]	3.7 x 10^{9}<br>*9.47±0.38*	6d
		DK-F1[V]	3.0 x 10^{6}<br>*6.25±0.66*	3d			DK-F1[V]	1.6 x 10^{7}<br>*7.15±0.26*	6d
		DK-1p8[L]	8.3 x 10^{7}<br>*7.92±0.14*	3d			DK-1p8[L]	2.3 x 10^{8}<br>*8.3±0.32*	4d

French VHSV recombinant strains

At the time of CPE					Maximum viral production				
Line	MOI	Strain	Titer	Time	Line	MOI	Strain	Titer	Time
EPC	0.1	Wt[H]	5.5 x 10^{8}	2-3d	EPC	0.1	Wt[H]	4.3 x 10^{8}<br>*8.58±0.2*	5d
		NV-R116Y[H]	7.6 x 10^{8}	2-3d			NV-R116Y[H]	5.6 x 10^{8}<br>*8.72±0.21*	5d
		NV-R116S[M]	6.4 x 10^{8}	2-3d			NV-R116S[M]	1.9 x 10^{7}<br>*7.22±0.31*	5d
		DD224[L]	1.1 x 10^{9}	2-3d			DD224[L]	1.4 x 10^{8}<br>*8.00±0.43*	5d
		N-K46G[L]	NA				N-K46G[L]	2.0 x 10^{7}<br>*7.28±0.12*	*5d
		NV_N[L]	NA				NV_N[L]	1.3 x 10^{6}<br>*6.00±0.43*	*5d

[1]CPE: Cytopathic Effect; [2]Line: cell line tested; [3]MOI: multiplicity of infection; [4]Titer: $TCID_{50}$/ml (below: Average±Standard Deviation, when 3 replicas were titrated); ND: Not Detected; NA: Not Assayed; NDm: Not Determined; N/Av: not available. *Considering the maximum time tested with the remaining strain (see Fig 10).
