# Supplementary material for: Steps of the Replication Cycle of the Viral Haemorrhagic Septicaemia Virus (VHSV) Affecting Its Virulence on Fish
Source: Animals (Basel). 2020 Dec 1;10(12):2264. doi: 10.3390/ani10122264 (PMC7761041; doi:10.3390/ani10122264)
Supplement: Supplementary file 1 [file animals-10-02264-s001.zip › Supplementary items-wo Fig Legend-2/Supplementary Table 1-Adsorption Spanish strains-vs3.pdf]

Supplementary Table 1.- Adsorption capacity of the Spanish VHSV strains

Cell line: EPC

Adsorption time	Strain	Method	AAE[1]	RAE[2]	EOA[3]
15 min	Sm2897[H]	TCID	97.55±1.25	97.35±1.24	99.80±0.02
		qPCR	87.97±9.13	87.29±9.28	99.23±0.30
	DC1412[L]	TCID	38.51±8.53	35.16±8.37	91.31±1.91
		qPCR	41.39±19.96	40.13±19.87	96.95±3.72
30 min	Sm2897[H]	TCID	95.30*	95.05*	99.74*
		qPCR	NP	NP	NP
	DC1412[L]	TCID	35.94*	33.17*	92.30*
		qPCR	NP	NP	NP
45 min	Sm2897[H]	TCID	96.04±1.42	95.83±1.42	99.78±0.003
		qPCR	90.62±5.06	90.09±4.86	99.42±0.19
	DC1412[L]	TCID	52.44±14.35	48.91±14.19	93.27±1.51
		qPCR	48.30±19.30	46.95±18.36	97.19±1.09
60 min	Sm2897[H]	TCID	95.80*	95.59*	99.87*
		qPCR	NP	NP	NP
	DC1412[L]	TCID	39.44*	35.72*	90.58*
		qPCR	NP	NP	NP

Cell line: RTG-2

Adsorption time	Strain	Method	AAE	RAE	EOA
30 min	Sm2897[H]	TCID	91.48±2.51	91.44±2.54	99.95±0.06
		qPCR	83.02±10.51	82.83±10.52	99.77±0.25
	DC1412[L]	TCID	52.06±13.69	51.96±13.69	99.81±0.05
		qPCR	60.53±10.97	60.39±11.06	99.76±0.19

[1]Apparent adsorption efficacy: AAE=TAV (total adsorbed virus)/TIV (total inoculated virus) × 100; [2]Real adsorption efficacy: RAE=IAV (irreversibly adsorbed virus)/TIV × 100; [3]Efficiency of adsorption: EOA=IAV/TAV × 100. *, Performed with only 2 replicas. NP, Not Performed
