# Supplementary material for: Steps of the Replication Cycle of the Viral Haemorrhagic Septicaemia Virus (VHSV) Affecting Its Virulence on Fish
Source: Animals (Basel). 2020 Dec 1;10(12):2264. doi: 10.3390/ani10122264 (PMC7761041; doi:10.3390/ani10122264)
Supplement: Supplementary file 1 [file animals-10-02264-s001.zip › Supplementary items-wo Fig Legend-2/Supplementary Table 2-Adsorption Italian strains-vs2.pdf]

Supplementary Table 2.- Adsorption capacity of the Italian VHSV strains

Cell line: EPC

Adsorption time	Strain	Method	AAE[1]	RAE[2]	EOA[3]
30 min	TN68[H]	TCID	86.80±11.10	86.80±11.66	98.85±0.83
		qPCR	79.23±15.88	77.97±15.92	98.41±0.82
	TN470[H]	TCID	79.95±6.87	79.76±6.96	99.77±0.73
		qPCR	66.59±17.88	66.22±18.13	99.45±0.73
	TN480[L]	TCID	50.81±13.90	50.02±13.78	98.44±0.59
		qPCR	57.28±6.34	56.27±6.44	98.22±0.60

Cell line: RTG-2

Adsorption time	Strain	Method	AAE[1]	RAE[2]	EOA[3]
15 min	TN68[H]	TCID	92.36±7.33	92.31±7.37	99.95±0.05
		qPCR	83.57±6.68	81.56±7.92	97.59±2.59
	TN470[H]	TCID	81.22±13.76	81.11±13.84	99.87±0.13
		qPCR	78.98±10.48	78.01±10.03	98.78±0.45
	TN480[L]	TCID	36.97±11.97	35.97±11.97	97.30±1.18
		qPCR	43.75±16.55	42.90±16.72	98.06±1.90
30 min	TN68[H]	TCID	95.14±4.48	95.09±4.47	99.95±0.04
		qPCR	88.81±8.82	88.10±9.14	99.19±0.64
	TN80[H]	TCID	87.01±4.55	84.79±4.51	97.46±1.49
		qPCR	88.56±14.22	77.66±22.36	87.69±18.55
	TN470[H]	TCID	77.26±6.87	77.17±6.91	99.88±0.10
		qPCR	75.24±16.13	74.54±15.94	99.07±0.44
	TN480[L]	TCID	45.27±1.72	44.41±1.54	98.11±0.52
		qPCR	36.40±4.94	35.77±4.79	98.26±0.94
45 min	TN68[H]	TCID	95.27±4.18	95.17±4.12	99.90±0.15
		qPCR	89.86±4.53	88.58±4.94	98.58±0.91
	TN470[H]	TCID	77.37±7.03	77.28±7.07	99.88±0.10
		qPCR	77.39±16.32	76.65±16.64	99.05±0.87
	TN480[L]	TCID	52.50±15.12	51.79±14.87	98.65±0.08
		qPCR	48.06±18.09	47.36±18.73	98.55±3.12
60 min	TN68[H]	TCID	86.80±20.25	85.80±21.33	98.85±2.03
		qPCR	87.42±8.09	86.32±8.44	98.74±0.59
	TN470[H]	TCID	71.38±3.54	71.17±3.69	99.71±0.37
		qPCR	72.00±13.87	71.36±14.37	99.11±0.93
	TN480[L]	TCID	44.14±0.80	43.43±0.81	98.40±0.59
		qPCR	40.31±19.12	39.53±19.07	98.06±1.30

[1]Apparent adsorption efficacy: AAE=TAV (total adsorbed virus)/TIV (total inoculated virus) × 100; [2]Real adsorption efficacy: RAE=IAV (irreversibly adsorbed virus)/TIV × 100; [3]Efficiency of adsorption: EOA=IAV/TAV × 100.
