# Supplementary material for: Steps of the Replication Cycle of the Viral Haemorrhagic Septicaemia Virus (VHSV) Affecting Its Virulence on Fish
Source: Animals (Basel). 2020 Dec 1;10(12):2264. doi: 10.3390/ani10122264 (PMC7761041; doi:10.3390/ani10122264)
Supplement: Supplementary file 1 [file animals-10-02264-s001.zip › Supplementary items-wo Fig Legend-2/Supplementary Table 8-Viral productio differences.docx]

Supplementary Table 8.- **t** Test comparison of maximum production values

| A/Comparison of titers data (TCID_50_/ml) | | | | | | | |  |  |  |  |  |  | | | | | | | | | | | | | | | |
| --- | --- | --- | --- | --- | --- | --- | --- | --- | --- | --- | --- | --- | --- | --- | --- | --- | --- | --- | --- | --- | --- | --- | --- | --- | --- | --- | --- | --- |
| Spanish strains | | | | | | | |  |  |  |  |  | Italian strains | | | | | | | | | | | | | | | |
|  | EPC | | |  | RTG-2 | | |  |  |  |  |  |  | EPC | | | | | | |  | RTG-2 | | | | | | |
|  | H |  | L |  | H |  | L |  |  |  |  |  |  | H68 |  | H80 |  | H470 |  | L480 |  | H68 |  | H80 |  | H470 |  | L480 |
| H | - |  | 0.0039 |  | - |  | 0.0082 |  |  |  |  |  | H68 | - |  | NA |  | 0.0053 |  | 0.0132 |  | - |  | 0.1571 |  | 0.3868 |  | 0.0132 |
| L | - |  | - |  | - |  | - |  |  |  |  |  | H80 | - |  | - |  | NA |  | NA |  | - |  | - |  | 0.7934 |  | 0.0033 |
|  |  |  |  |  |  |  |  |  |  |  |  |  | H470 | - |  | - |  | - |  | 0.0011 |  | - |  | - |  | - |  | 0.0136 |
|  |  |  |  |  |  |  |  |  |  |  |  |  | L480 | - |  | - |  | - |  | - |  | - |  | - |  | - |  | - |
|  |  |  |  |  |  |  |  |  |  |  |  |  |  |  |  |  |  |  |  |  |  |  |  |  |  |  |  |  |
|  |  |  |  |  |  |  |  |  |  |  |  |  |  |  |  |  |  |  |  |  |  |  |  |  |  |  |  |  |
| Danish strains | | | | | | | | | | | |  | French recombinants | | | | | | | | | | | |  |  |  |  |
|  | RTG-2 | | | | |  | BF-2 | | | | |  |  | EPC | | | | | | | | | | |  |  |  |  |
|  | H |  | V |  | L |  | H |  | V |  | L |  |  | Wt |  | DD |  | Y[H] |  | S[H] |  | NK46[L] |  | NV_N[L] |  |  |  |  |
| H | - |  | >0.9999 |  | 0.0103 |  | - |  | 0.0010 |  | 0.0148 |  | Wt | - |  | 0.1242 |  | 0.5518 |  | 0.0049 |  | 0.0019 |  | 0.0010 |  |  |  |  |
| V | - |  | - |  | 0.0055 |  | - |  | - |  | 0.0092 |  | DD | - |  | - |  | 0.0611 |  | 0.0626 |  | 0.0503 |  | 0.0048 |  |  |  |  |
| L | - |  | - |  | - |  | - |  | - |  | - |  | Y[H] | - |  | - |  | - |  | 0.0022 |  | 0.0005 |  | 0.0006 |  |  |  |  |
|  |  |  |  |  |  |  |  |  |  |  |  |  | S[H] | - |  | - |  | - |  | - |  | 0.7415 |  | 0.0164 |  |  |  |  |
|  |  |  |  |  |  |  |  |  |  |  |  |  | NK46[L] | - |  | - |  | - |  | - |  | - |  | 0.0077 |  |  |  |  |
|  |  |  |  |  |  |  |  |  |  |  |  |  | NV_N[L] | - |  | - |  | - |  | - |  | - |  | - |  |  |  |  |

| B/Comparison of RNA copies (determined by Rt-qPCR) | | | | | | | | | | |  | |  | |  | |  | | | | | | | | | | | | | | | | | | | | | | | | | | | | | | | |
| --- | --- | --- | --- | --- | --- | --- | --- | --- | --- | --- | --- | --- | --- | --- | --- | --- | --- | --- | --- | --- | --- | --- | --- | --- | --- | --- | --- | --- | --- | --- | --- | --- | --- | --- | --- | --- | --- | --- | --- | --- | --- | --- | --- | --- | --- | --- | --- | --- |
| Spanish strains | | | | | | | |  |  | |  | |  | |  | | Italian strains | | | | | | | | | | | | | | | | | | | | | | | | | | | | | | | |
|  | EPC | | |  | RTG-2 | | |  |  | |  | |  | |  | |  | | EPC | | | | | | | | | | | | | |  | | RTG-2 | | | | | | | | | | | | | |
|  | H |  | L |  | H |  | L |  |  | |  | |  | |  | |  | | H68 | |  | | H80 | |  | | H470 | |  | | L480 | |  | | H68 | |  | | H80 | |  | | H470 | |  | | L480 | |
| H | - |  | 0.9012 |  | - |  | 0.6864 |  |  | |  | |  | |  | | H68 | | - | |  | | NA | |  | | 0.5936 | |  | | 0.7846 | |  | | - | |  | | 0.0674 | |  | | 0.1032 | |  | | 0.0552 | |
| L | - |  | - |  | - |  | - |  |  | |  | |  | |  | | H80 | | - | |  | | - | |  | | NA | |  | | NA | |  | | - | |  | | - | |  | | 0.0059 | |  | | 0.0112 | |
|  |  |  |  |  |  |  |  |  |  | |  | |  | |  | | H470 | | - | |  | | - | |  | | - | |  | | 0.8781 | |  | | - | |  | | - | |  | | - | |  | | 0.2713 | |
|  |  |  |  |  |  |  |  |  |  | |  | |  | |  | | L480 | | - | |  | | - | |  | | - | |  | | - | |  | | - | |  | | - | |  | | - | |  | | - | |
|  |  |  |  |  |  |  |  |  |  | |  | |  | |  | |  | |  | |  | |  | |  | |  | |  | |  | |  | |  | |  | |  | |  | |  | |  | |  | |
|  |  |  |  |  |  |  |  |  |  | |  | |  | |  | |  | |  | |  | |  | |  | |  | |  | |  | |  | |  | |  | |  | |  | |  | |  | |  | |
| Danish strains | | | | | | | | | | | | | | |  | | French recombinants | | | | | | | | | | | | | | | | | | | | | | | |  | |  | |  | |  | |
|  | RTG-2 | | | | |  | BF-2 | | | | | | | |  | |  | | EPC | | | | | | | | | | | | | | | | | | | | | |  | |  | |  | |  | |
|  | H |  | V |  | L |  | H |  | V |  | | L | |  | |  | | Wt | |  | | DD | |  | | Y[H] | |  | | S[H] | |  | | NK46[L] | |  | | NV_N[L] | |  | |  | |  | |  | |  |
| H | - |  | 0.4128 |  | 0.0006 |  | - |  | NA |  | | NA | |  | | Wt | | - | |  | | 0.2422 | |  | | 0.5658 | |  | | 0.2213 | |  | | 0.0408 | |  | | 0.0011 | |  | |  | |  | |  | |  |
| V | - |  | - |  | 0.0008 |  | - |  | - |  | | NA | |  | | DD | | - | |  | | - | |  | | 0.6705 | |  | | 0.4956 | |  | | 0.0553 | |  | | <0.0001 | |  | |  | |  | |  | |  |
| L | - |  | - |  | - |  | - |  | - |  | | - | |  | | Y[H] | | - | |  | | - | |  | | - | |  | | 0.4172 | |  | | 0.0971 | |  | | 0.0022 | |  | |  | |  | |  | |  |
|  |  |  |  |  |  |  |  |  |  |  | |  | |  | | S[H] | | - | |  | | - | |  | | - | |  | | - | |  | | 0.4820 | |  | | 0.0141 | |  | |  | |  | |  | |  |
|  |  |  |  |  |  |  |  |  |  |  | |  | |  | | NK46[L] | | - | |  | | - | |  | | - | |  | | - | |  | | - | |  | | 0.0054 | |  | |  | |  | |  | |  |
|  |  |  |  |  |  |  |  |  |  |  | |  | |  | | NV_N[L] | | - | |  | | - | |  | | - | |  | | - | |  | | - | |  | | - | |  | |  | |  | |  | |  |

Names of strains are substituted by H or L (regarding their level of virulence), or by an abbreviated name when necessary. Data shown correspond to the P values; P≤0.05 are interpreted as significant differences. NA: Not Assayed.
