# Supplementary material for: Steps of the Replication Cycle of the Viral Haemorrhagic Septicaemia Virus (VHSV) Affecting Its Virulence on Fish
Source: Animals (Basel). 2020 Dec 1;10(12):2264. doi: 10.3390/ani10122264 (PMC7761041; doi:10.3390/ani10122264)
Supplement: Supplementary file 1 [file animals-10-02264-s001.zip › Supplementary items-wo Fig Legend-2/Supplementary Table 7-Viral production_RNACopies-vs3.pdf]

Supplementary Table 7- Viral production, quantified by qPCR (RNA copies)

Spanish VHSV strains									
At the time of CPE[1]					Maximum viral production				
Line[2]	MOI[3]	Strain	Titer[4]	Time	Line	MOI	Strain	Titer	Time
EPC	0.1	Sm2897[H]	1.9 x 10^8	3d	EPC	0.1	Sm2897[H]	1.0 x 10^8<br>*8.02±0.05*	6d
		DC1412[L]	6.1 x 10^7	3d			DC1412[L]	1.1 x 10^8<br>*8.00±0.17*	7d
RTG-2	0.01	Sm2897[H]	1.4 x 10^7	4-5d	RTG-2	0.1	Sm2897[H]	3.4 x 10^7<br>*7.52±0.14*	6d
		DC1412[L]	1.5 x 10^2				DC1412[L]	5.2 x 10^7<br>*7.62±0.39*	3d
	0.1	Sm2897[H]	1.8 x 10^7	3d					
		DC1412[L]	5.2 x 10^7	3d					
	1.0	Sm2897[H]	1.2 x 10^8	2-3d					
		DC1412[L]	1.8 x 10^8	3d					

Italian VHSV strains									
At the time of CPE					Maximum viral production				
Line	MOI	Strain	Titer	Time	Line	MOI	Strain	Titer	Time
EPC	0.1	TN68[H]	8.5 x 10^7	N/Av	EPC	0.1	TN68[H]	7.8 x 10^7<br>*7.87±0.15*	3d
		TN80[H]	NA				TN80[H]	NA	
		TN470[H]	7.5 x 10^7	N/Av			TN470[H]	9.3 x 10^7<br>*7.76±0.64*	3d
		TN480[L]	7.4 x 10^7	N/Av			TN480[L]	6.7 x 10^7<br>*7.83±0.03*	3d
RTG-2	0.01	TN68[H]	1.1 x 10^8	3-4d	RTG-2	0.1	TN68[H]	2.0 x 10^7<br>*7.28±0.15*	7d
		TN80[H]	NA				TN80[H]	1.1 x 10^7<br>*7.05±0.06*	4d
		TN470[H]	1.0 x 10^8	3-4d			TN470[H]	3.4 x 10^7<br>*7.52±0.14*	3d
		TN480[L]	4.1 x 10^7	3-4d			TN480[L]	6.0 x 10^7<br>*7.74±0.26*	2.5d
	0.1	TN68[H]	5.4 x 10^7	2-3d					
		TN80[H]	NA						
		TN470[H]	1.0 x 10^8	3d					
		TN480[L]	1.1 x 10^8	2-3d					
	1.0	TN68[H]	1.0 x 10^8	2d					
		TN80[H]	NA						
		TN470[H]	2.2 x 10^8	2-3d					
		TN480[L]	8.7 x 10^7	2d					

Danish VHSV strains

At the time of CPE					Maximum viral production				
Line	MOI	Strain	Titer	Time	Line	MOI	Strain	Titer	Time
RTG-2	0.1	DK3592[H]	9.4×10^7<br>*7.97±0.07*	3d	RTG-2	0.1	DK3592[H]	4.8×10^7<br>*7.70±0.07*	7.5d
		DK-F1[V]	8.8×10^7<br>*7.75±0.30*	2-3d			DK-F1[V]	6.2×10^7<br>*7.77±0.16*	2d
		DK-1p8[L]	7.7×10^6<br>*6.18±0.25*	3d			DK-1p8[L]	1.7×10^6<br>*6.18±0.25*	4d
BF-2	0.1	DK3592[H]	1.2×10^8	3d	BF-2	0.1	DK3592[H]	NDm	
		DK-F1[V]	2.3×10^6	3d			DK-F1[V]	NDm	
		DK-1p8[L]	2.3×10^6	3d			DK-1p8[L]	NDm	

French VHSV recombinant strains

At the time of CPE					Maximum viral production				
Line	MOI	Strain	Titer	Time	Line	MOI	Strain	Titer	Time
EPC	0.1	Wt[H]	7.5×10^8	2-3d	EPC	0.1	Wt[H]	4.5×10^8<br>*8.62±0.19*	5d
		NV-R116Y[H]	4.8×10^9	2-3d			NV-R116Y[H]	3.6×10^8<br>*8.52±0.21*	5d
		NV-R116S[M]	5.0×10^7	2-3d			NV-R116S[M]	2.5×10^8<br>*8.34±0.28*	5d
		DD224[L]	1.3×10^9	2-3d			DD224[L]	2.9×10^8<br>*8.47±0.06*	5d
		N-K46G[L]	NA				N-K46G[L]	1.7×10^8<br>*8.20±0.16*	5d*
		NV_N[L]	NA				NV_N[L]	4.6×10^7<br>*7.66±0.09*	5d*

[1]CPE: Cytopathic Effect; [2]Line: cell line tested; [3]MOI: multiplicity of infection; [4]Titer: $TCID_{50}$/ml (below: Average±Standard Deviation, when 3 replicas were titrated); ND: Not Detected; NA: Not Assayed; NDm: Not Determined; N/Av: not available. *Considering the maximum time tested with the remaining strain (see Fig 10).
