# Supplementary material for: Steps of the Replication Cycle of the Viral Haemorrhagic Septicaemia Virus (VHSV) Affecting Its Virulence on Fish
Source: Animals (Basel). 2020 Dec 1;10(12):2264. doi: 10.3390/ani10122264 (PMC7761041; doi:10.3390/ani10122264)
Supplement: Supplementary file 1 [file animals-10-02264-s001.zip › Supplementary items-wo Fig Legend-2/Supplementary Table 11-Corr coef and stat diffs between curves_DK strains-vs3.docx]

Supplementary Table 11.- Correlation coefficient and statistical differences between replication curves: Danish strains

| A.-Correlation between replication curves | | | | | | | | | | | | | | | | | | |
| --- | --- | --- | --- | --- | --- | --- | --- | --- | --- | --- | --- | --- | --- | --- | --- | --- | --- | --- |
|  |  | BF-2 | | | | | | | | | | | | | | | | |
|  |  | DK3592[H] | | | | |  | DK-F1[V] | | | | |  | DK1p8[L] | | | | |
|  |  | Intr |  | Extr |  | Prog |  | Intr |  | Extr |  | Prog |  | Intr |  | Extr |  | Prog |
| DK3592[H] | Intr | - |  | - |  | - |  | - |  | - |  | - |  | - |  | - |  | - |
|  | Extr | - |  | - |  | - |  | - |  | - |  | - |  | - |  | - |  | - |
|  | Prog | - |  | - |  | - |  | - |  | - |  | 0.9816 |  | - |  | - |  | 0.9968 |
| DK-F1[V] | Intr | - |  | - |  | - |  | - |  | - |  | - |  | - |  | - |  | - |
|  | Extr | - |  | - |  | - |  | - |  | - |  | - |  | - |  | - |  | - |
|  | Prog | - |  | - |  | - |  | - |  | - |  | - |  | - |  | - |  | 0.9819 |
| DK1p8[L] | Intr | - |  | - |  | - |  | - |  | - |  | - |  | - |  | - |  | - |
|  | Extr | - |  | - |  | - |  | - |  | - |  | - |  | - |  | - |  | - |
|  | Prog | - |  | - |  | - |  | - |  | - |  | - |  | - |  | - |  | - |
|  |  |  |  |  |  |  |  |  |  |  |  |  |  |  |  |  |  |  |
|  |  | RTG-2 | | | | | | | | | | | | | | | | |
|  |  | DK3592[H] | | | | |  | DK-F1[V] | | | | |  | DK1p8[L] | | | | |
|  |  | Intr |  | Extr |  | Prog |  | Intr |  | Extr |  | Prog |  | Intr |  | Extr |  | Prog |
| DK3592[H] | Intr | - |  | 0.9783 |  | 0.9738 |  | 0.9736 |  | - |  | - |  | 0.9306 |  | - |  | - |
|  | Extr | - |  | - |  | 0.9315 |  | - |  | 0.9453 |  | - |  | - |  | 0.8988 |  | - |
|  | Prog | - |  | - |  | - |  | - |  | - |  | 0.9023 |  | - |  | - |  | 0.9070 |
| DK-F1[V] | Intr | - |  | - |  | - |  | - |  | 0.9780 |  | 0.9322 |  | 0.8710 |  | - |  | - |
|  | Extr | - |  | - |  | - |  | - |  | - |  | 0.9483 |  | - |  | 0.7832 |  | - |
|  | Prog | - |  | - |  | - |  | - |  | - |  | - |  | - |  | - |  | 0.7141 |
| DK1p8[L] | Intr | - |  | - |  | - |  | - |  | - |  | - |  | - |  | 0.9219 |  | 0.9700 |
|  | Extr | - |  | - |  | - |  | - |  | - |  | - |  | - |  | - |  | 0.8506 |
|  | Prog | - |  | - |  | - |  | - |  | - |  | - |  | - |  | - |  | - |
|  |  |  |  |  |  |  |  |  |  |  |  |  |  |  |  |  |  |  |
| B.-Differences between replication curves (2 ways ANOVA; data are shown as P values) | | | | | | | | | | | | | | | | | | |
|  |  | BF-2 | | | | | | | | | | | | | | | | |
|  |  | DK3592[H] | | | | |  | DK-F1[V] | | | | |  | DK1p8[L] | | | | |
|  |  | Intr |  | Extr |  | Prog |  | Intr |  | Extr |  | Prog |  | Intr |  | Extr |  | Prog |
| DK3592[H] | Intr | - |  | - |  | - |  | - |  | - |  | - |  | - |  | - |  | - |
|  | Extr | - |  | - |  | - |  | - |  | - |  | - |  | - |  | - |  | - |
|  | Prog | - |  | - |  | - |  | - |  | - |  | 0.0001 |  | - |  | - |  | 0.0037 |
| DK-F1[V] | Intr | - |  | - |  | - |  | - |  | - |  | - |  | - |  | - |  | - |
|  | Extr | - |  | - |  | - |  | - |  | - |  | - |  | - |  | - |  | - |
|  | Prog | - |  | - |  | - |  | - |  | - |  | - |  | - |  | - |  | 0.0001 |
| DK1p8[L] | Intr | - |  | - |  | - |  | - |  | - |  | - |  | - |  | - |  | - |
|  | Extr | - |  | - |  | - |  | - |  | - |  | - |  | - |  | - |  | - |
|  | Prog | - |  | - |  | - |  | - |  | - |  | - |  | - |  | - |  | - |
|  |  |  |  |  |  |  |  |  |  |  |  |  |  |  |  |  |  |  |
|  |  | RTG-2 | | | | | | | | | | | | | | | | |
|  |  | DK3592[H] | | | | |  | DK-F1[V] | | | | |  | DK1p8[L] | | | | |
|  |  | Intr |  | Extr |  | Prog |  | Intr |  | Extr |  | Prog |  | Intr |  | Extr |  | Prog |
| DK3592[H] | Intr | - |  | 0.0684 |  | 0.1002 |  | 0.5573 |  | - |  | - |  | 0.0204 |  | - |  | - |
|  | Extr | - |  | - |  | 0.0030 |  | - |  | 0.0138 |  | - |  | - |  | <0.0001 |  | - |
|  | Prog | - |  | - |  | - |  | - |  | - |  | 0.0004 |  | - |  | - |  | 0.0001 |
| DK-F1[V] | Intr | - |  | - |  | - |  | - |  | 0.3439 |  | 0.2471 |  | 0.0017 |  | - |  | - |
|  | Extr | - |  | - |  | - |  | - |  | - |  | 0.0009 |  | - |  | 0.0005 |  | - |
|  | Prog | - |  | - |  | - |  | - |  | - |  | - |  | - |  | - |  | 0.0688 |
| DK1p8[L] | Intr | - |  | - |  | - |  | - |  | - |  | - |  | - |  | 0.0063 |  | 0.0478 |
|  | Extr | - |  | - |  | - |  | - |  | - |  | - |  | - |  | - |  | 0.0257 |
|  | Prog | - |  | - |  | - |  | - |  | - |  | - |  | - |  | - |  | - |

| C.-Differences between replication curves (Average difference between time points titers) | | | | | | | | | | | | | | | | | | |
| --- | --- | --- | --- | --- | --- | --- | --- | --- | --- | --- | --- | --- | --- | --- | --- | --- | --- | --- |
|  |  | BF-2 | | | | | | | | | | | | | | | | |
|  |  | DK3592[H] | | | | |  | DK-F1[V] | | | | |  | DK1p8[L] | | | | |
|  | AvTD SD | Intr |  | Extr |  | Prog |  | Intr |  | Extr |  | Prog |  | Intr |  | Extr |  | Prog |
| DK3592[H] | Intr | - |  | - |  | - |  | - |  | - |  | - |  | - |  | - |  | - |
|  | Extr | - |  | - |  | - |  | - |  | - |  | - |  | - |  | - |  | - |
|  | Prog | - |  | - |  | - |  | - |  | - |  | 0.88 |  | - |  | - |  | 1.63 |
| DK-F1[V] | Intr | - |  | - |  | - |  | - |  | - |  | - |  | - |  | - |  | - |
|  | Extr | - |  | - |  | - |  | - |  | - |  | - |  | - |  | - |  | - |
|  | Prog | - |  | - |  | 0.71 |  | - |  | - |  | - |  | - |  | - |  | 0.78 |
| DK1p8[L] | Intr | - |  | - |  | - |  | - |  | - |  | - |  | - |  | - |  | - |
|  | Extr | - |  | - |  | - |  | - |  | - |  | - |  | - |  | - |  | - |
|  | Prog | - |  | - |  | 0.36 |  | - |  | - |  | 0.74 |  | - |  | - |  | - |
|  |  |  |  |  |  |  |  |  |  |  |  |  |  |  |  |  |  |  |
|  |  | RTG-2 | | | | | | | | | | | | | | | | |
|  |  | DK3592[H] | | | | |  | DK-F1[V] | | | | |  | DK1p8[L] | | | | |
|  | AvTD SD | Intr |  | Extr |  | Prog |  | Intr |  | Extr |  | Prog |  | Intr |  | Extr |  | Prog |
| DK3592[H] | Intr | - |  | 0.63 |  | 0.57 |  | 0.57 |  | - |  | - |  | 0.96 |  | - |  | - |
|  | Extr | 0.47 |  | - |  | 0.80 |  | - |  | 1.05* |  | - |  | - |  | 1.44* |  | - |
|  | Prog | 0.49 |  | 1.11 |  | - |  | - |  | - |  | 1.34* |  | - |  | - |  | 1.05* |
| DK-F1[V] | Intr | 0.54 |  | - |  | - |  | - |  | 0.69 |  | 1.00* |  | 1.33* |  | - |  | - |
|  | Extr | - |  | 0.97 |  | - |  | 0.42 |  | - |  | 0.89 |  | - |  | 1.99* |  | - |
|  | Prog | - |  | - |  | 1.24 |  | 1.13 |  | 0.99 |  | - |  | - |  | - |  | 1.92* |
| DK1p8[L] | Intr | 0.96 |  | - |  | - |  | 0.60 |  | - |  | - |  | - |  | 0.78 |  | 0.67 |
|  | Extr | - |  | 0.88 |  | - |  | - |  | 1.00 |  | - |  | 0.98 |  | - |  | 0.91 |
|  | Prog | - |  | - |  | 0.78 |  | - |  | - |  | 1.33 |  | 0.42 |  | 1.36 |  | - |

A.- In the first part of the table, correlation between curves is given by the correlation coefficient values (**r**), being **r**=1 the maximum correlation between 2 curves; correlation is confirmed by P≤0.05; *no significant correlation values (**r** values with P>0.05). B.- In the second part of the table, the results of a SIDAK multiple comparison 2-way ANOVA test was employed (differences considered significant only for values of P≤0.01). C.- The third part shows the average differences of titer in each time point between two curves (average differences higher than 1 Log_10_ are considered significant and labelled with a *****). AvTD: Average titer differences (data in blue color; from 3 replicas); SD: Standard deviation.
