# Supplementary material for: Steps of the Replication Cycle of the Viral Haemorrhagic Septicaemia Virus (VHSV) Affecting Its Virulence on Fish
Source: Animals (Basel). 2020 Dec 1;10(12):2264. doi: 10.3390/ani10122264 (PMC7761041; doi:10.3390/ani10122264)
Supplement: Supplementary file 1 [file animals-10-02264-s001.zip › Supplementary items-wo Fig Legend-2/Supplementary Table 1-Adsorption Spanish strains-vs3.docx]

Supplementary Table 1.- Adsorption capacity of the Spanish VHSV strains

| *Cell line: EPC* |  |  |  |  |  |  |  |  |  |  |
| --- | --- | --- | --- | --- | --- | --- | --- | --- | --- | --- |
| Adsorption time |  | Strain |  | Method |  | AAE^1^ |  | RAE^2^ |  | EOA^3^ |
| 15 min |  | Sm2897[H] |  | TCID |  | 97.55±1.25 |  | 97.35±1.24 |  | 99.80±0.02 |
|  |  |  |  | qPCR |  | 87.97±9.13 |  | 87.29±9.28 |  | 99.23±0.30 |
|  |  | DC1412[L] |  | TCID |  | 38.51±8.53 |  | 35.16±8.37 |  | 91.31±1.91 |
|  |  |  |  | qPCR |  | 41.39±19.96 |  | 40.13±19.87 |  | 96.95±3.72 |
| 30 min |  | Sm2897[H] |  | TCID |  | 95.30* |  | 95.05* |  | 99.74* |
|  |  |  |  | qPCR |  | NP |  | NP |  | NP |
|  |  | DC1412[L] |  | TCID |  | 35.94* |  | 33.17* |  | 92.30* |
|  |  |  |  | qPCR |  | NP |  | NP |  | NP |
| 45 min |  | Sm2897[H] |  | TCID |  | 96.04±1.42 |  | 95.83±1.42 |  | 99.78±0.003 |
|  |  |  |  | qPCR |  | 90.62±5.06 |  | 90.09±4.86 |  | 99.42±0.19 |
|  |  | DC1412[L] |  | TCID |  | 52.44±14.35 |  | 48.91±14.19 |  | 93.27±1.51 |
|  |  |  |  | qPCR |  | 48.30±19.30 |  | 46.95±18.36 |  | 97.19±1.09 |
| 60 min |  | Sm2897[H] |  | TCID |  | 95.80* |  | 95.59* |  | 99.87* |
|  |  |  |  | qPCR |  | NP |  | NP |  | NP |
|  |  | DC1412[L] |  | TCID |  | 39.44* |  | 35.72* |  | 90.58* |
|  |  |  |  | qPCR |  | NP |  | NP |  | NP |
|  |  |  |  |  |  |  |  |  |  |  |
| *Cell line: RTG-2* |  |  |  |  |  |  |  |  |  |  |
| Adsorption time |  | Strain |  | Method |  | AAE |  | RAE |  | EOA |
| 30 min |  | Sm2897[H] |  | TCID |  | 91.48±2.51 |  | 91.44±2.54 |  | 99.95±0.06 |
|  |  |  |  | qPCR |  | 83.02±10.51 |  | 82.83±10.52 |  | 99.77±0.25 |
|  |  | DC1412[L] |  | TCID |  | 52.06±13.69 |  | 51.96±13.69 |  | 99.81±0.05 |
|  |  |  |  | qPCR |  | 60.53±10.97 |  | 60.39±11.06 |  | 99.76±0.19 |

^1^Apparent adsorption efficacy: AAE=TAV (total adsorbed virus)/TIV (total inoculated virus) 🞪 100; ^2^Real adsorption efficacy: RAE=IAV (irreversibly adsorbed virus)/TIV 🞪 100; ^3^Efficiency of adsorption: EOA=IAV/TAV 🞪 100. *, Performed with only 2 replicas. NP, Not Performed
