# Supplementary material for: Steps of the Replication Cycle of the Viral Haemorrhagic Septicaemia Virus (VHSV) Affecting Its Virulence on Fish
Source: Animals (Basel). 2020 Dec 1;10(12):2264. doi: 10.3390/ani10122264 (PMC7761041; doi:10.3390/ani10122264)
Supplement: Supplementary file 1 [file animals-10-02264-s001.zip › Supplementary items-wo Fig Legend-2/Supplementary Table 8-Viral productio differences.pdf]

Supplementary Table 8.- **t** Test comparison of maximum production values

A/Comparison of titers data ($TCID_{50}$/ml)

Spanish strains

	EPC		RTG-2	
	H	L	H	L
H	-	0.0039	-	0.0082
L	-	-	-	-

Italian strains

	EPC				RTG-2			
	H68	H80	H470	L480	H68	H80	H470	L480
H68	-	NA	0.0053	0.0132	-	0.1571	0.3868	0.0132
H80	-	-	NA	NA	-	-	0.7934	0.0033
H470	-	-	-	0.0011	-	-	-	0.0136
L480	-	-	-	-	-	-	-	-

Danish strains

	RTG-2			BF-2		
	H	V	L	H	V	L
H	-	>0.9999	0.0103	-	0.001<br>0	0.0148
V	-	-	0.0055	-	-	0.0092
L	-	-	-	-	-	-

French recombinants

	EPC					
	Wt	DD	Y[H]	S[H]	NK46[L]	NV_N[L]
Wt	-	0.1242	0.5518	0.0049	0.0019	0.0010
DD	-	-	0.0611	0.0626	0.0503	0.0048
Y[H]	-	-	-	0.0022	0.0005	0.0006
S[H]	-	-	-	-	0.7415	0.0164
NK46[L]	-	-	-	-	-	0.0077
NV_N[L]	-	-	-	-	-	-

B/Comparison of RNA copies (determined by Rt-qPCR)

Spanish strains

	EPC		RTG-2	
	H	L	H	L
H	-	0.9012	-	0.6864
L	-	-	-	-

Italian strains

	EPC				RTG-2			
	H6 8	H80	H470	L480	H68	H80	H470	L480
H68	-	NA	0.5936	0.7846	-	0.0674	0.1032	0.0552
H80	-	-	NA	NA	-	-	0.0059	0.0112
H470	-	-	-	0.8781	-	-	-	0.2713
L480	-	-	-	-	-	-	-	-

Danish strains

	RTG-2			BF-2		
	H	V	L	H	V	L
H	-	0.4128	0.0006	-	NA	NA
V	-	-	0.0008	-	-	NA
L	-	-	-	-	-	-

French recombinants

	EPC					
	Wt	DD	Y[H]	S[H]	NK46[L]	NV_N[L]
Wt	-	0.2422	0.5658	0.2213	0.0408	0.0011
DD	-	-	0.6705	0.4956	0.0553	<0.0001
Y[H]	-	-	-	0.4172	0.0971	0.0022
S[H]	-	-	-	-	0.4820	0.0141
NK46[L]	-	-	-	-	-	0.0054
NV_N[L]	-	-	-	-	-	-

Names of strains are substituted by H or L (regarding their level of virulence), or by an abbreviated name when necessary. Data shown correspond to the P values; P≤0.05 are interpreted as significant differences. NA: Not Assayed.
