# Supplementary material for: Steps of the Replication Cycle of the Viral Haemorrhagic Septicaemia Virus (VHSV) Affecting Its Virulence on Fish
Source: Animals (Basel). 2020 Dec 1;10(12):2264. doi: 10.3390/ani10122264 (PMC7761041; doi:10.3390/ani10122264)
Supplement: Supplementary file 1 [file animals-10-02264-s001.zip › Supplementary items-wo Fig Legend-2/Supplementary Table 9-Corr coef and stat diffs between curves_Sp strains-vs3.pdf]

Supplementary Table 9.- Correlation coefficient and statistical differences between replication curves: Spanish strains

A.- Correlation between replication curves

		EPC					
		Sm2897[H]			DC1412[L]		
		Intr	Extr	Prog	Intr	Extr	Prog
Sm[H]	Intr	-	0.983	0.860	0.945	-	-
	Extr	-	-	0.915	-	0.978	-
	Prog	-	-	-	-	-	0.988
DC[L]	Intr	-	-	-	-	0.961	0.874
	Extr	-	-	-	-	-	0.568
	Prog	-	-	-	-	-	-

		RTG-2					
		Sm2897[H]			DC1412[L]		
		Intr	Extr	Prog	Intr	Extr	Prog
Sm[H]	Intr	-	0.814	0.792	0.946	-	-
	Extr	-	-	0.950	-	0.958	-
	Prog	-	-	-	-	-	0.984
DC[L]	Intr	-	-	-	-	0.861	0.810
	Extr	-	-	-	-	-	0.912
	Prog	-	-	-	-	-	-

B.- Differences between replication curves (2 ways ANOVA; data are shown as P values)

		EPC					
		Sm2897[H]			DC1412[L]		
		Intr	Extr	Prog	Intr	Extr	Prog
Sm[H]	Intr	-	0.0002	-	0.0026	-	-
	Extr	-	-	0.0058	-	<0.0001	-
	Prog	-	-	-	-	-	0.0047
DC[L]	Intr	-	-	-	-	0.9274	
	Extr	-	-	-	-	-	0.0089
	Prog	-	-	-	-	-	-

		RTG-2					
		Sm2897[H]			DC1412[L]		
		Intr	Extr	Prog	Intr	Extr	Extr
Sm[H]	Intr	-	<0.0001	-	<0.0001	-	-
	Extr	-	-	0.0001	-	0.4277	-
	Prog	-	-	-	-		<0.0001
DC[L]	Intr	-	-	-	-	<0.0001	-
	Extr	-	-	-	-	-	0.0004
	Prog	-	-	-	-	-	-

C.- Differences between replication curves (Average difference between time points titers)

EPC		Sm2897[H]			DC1412[L]		
AvTD / SD		Intr	Extr	Prog	Intr	Extr	Prog
Sm[H]	Intr	-	1.41*	1.01*	0.46	-	-
	Extr	2.07	-	1.48*	-	0.41	-
	Prog	0.70	2.13	-	-	-	0.83
DC[L]	Intr	0.45	-	-	-	1.24*	1.15*
	Extr	-	0.33	-	1.93	-	1.16*
	Prog	-	-	0.45	0.87	1.89	-

RTG-2		Sm2897[H]			DC1412[L]		
AvTD / SD		Intr	Extr	Prog	Intr	Extr	Extr
Sm[H]	Intr	-	1.22*	1.33*	0.39	-	-
	Extr	1.20	-	0.73	-	0.40	-
	Prog	1.33	0.60	-	-	-	0.51
DC[L]	Intr	0.30	-	-	-	1.07*	2.33*
	Extr	-	0.42	-	0.87	-	2.12*
	Prog	-	-	2.77	2.54	2.69	-

A.- In the first part of the table, correlation between curves is given by the correlation coefficient values (**r**), being **r**=1 the maximum correlation between 2 curves; correlation is confirmed by P≤0.05; *no significant correlation values (**r** values with P>0.05). B.- In the second part of the table, the results of a SIDAK multiple comparison 2-way ANOVA test was employed (differences considered significant only for values of P≤0.01). C.- The third part shows the average differences of titer in each time point between two curves (average differences higher than 1 Log_{10} are considered significant and labelled with a *****). AvTD: Average titer differences (data in blue color; from 3 replicas); SD: Standard deviation.
