# Supplementary material for: Steps of the Replication Cycle of the Viral Haemorrhagic Septicaemia Virus (VHSV) Affecting Its Virulence on Fish
Source: Animals (Basel). 2020 Dec 1;10(12):2264. doi: 10.3390/ani10122264 (PMC7761041; doi:10.3390/ani10122264)
Supplement: Supplementary file 1 [file animals-10-02264-s001.zip › Supplementary items-wo Fig Legend-2/Supplementary Table 10-Corr coef and stat diffs between curves_It strains-vs5.docx]

Supplementary Table 10.- Correlation coefficient and statistical differences between replication curves: Italian strains

| A.- Correlation between replication curves | | | | | | | | | | | | | | | | | | | | | | | | |
| --- | --- | --- | --- | --- | --- | --- | --- | --- | --- | --- | --- | --- | --- | --- | --- | --- | --- | --- | --- | --- | --- | --- | --- | --- |
|  |  | EPC | | | | | | | | | | | | | | | | |  |  |  |  |  |  |
|  |  | TN68[H] | | | | |  | TN470[M] | | | | |  | TN480[L] | | | | |  |  |  |  |  |  |
|  |  | Intr |  | Extr |  | Prog |  | Intr |  | Extr |  | Prog |  | Intr |  | Extr |  | Prog |  |  |  |  |  |  |
| TN68[H] | Intr | - |  | 0.4290* |  | 0.9477 |  | 0.9931 |  | - |  | - |  | 0.9688 |  | - |  | - |  |  |  |  |  |  |
|  | Extr | - |  | - |  | 0.6910* |  | - |  | 0.9951 |  | - |  | - |  | 0.9903 |  | - |  |  |  |  |  |  |
|  | Prog | - |  | - |  | - |  | - |  | - |  | 0.9385 |  | - |  | - |  | 0.9889 |  |  |  |  |  |  |
| TN470[M] | Intr | - |  | - |  | - |  | - |  | 0.4006* |  | 0.8717 |  | 0.9715 |  | - |  | - |  |  |  |  |  |  |
|  | Extr | - |  | - |  | - |  | - |  | - |  | 0.6164* |  | - |  | 0.9741 |  | - |  |  |  |  |  |  |
|  | Prog | - |  | - |  | - |  | - |  | - |  | - |  | - |  | - |  | 0.8881 |  |  |  |  |  |  |
| TN480[L] | Intr | 0.0066 |  | - |  | - |  | 0.0057 |  | - |  | - |  | - |  | 0.4428* |  | 0.9350 |  |  |  |  |  |  |
|  | Extr | - |  | 0.0012 |  | - |  | - |  | 0.0050 |  | - |  | - |  | - |  | 0.6920* |  |  |  |  |  |  |
|  | Prog | - |  | - |  | 0.0014 |  | - |  | - |  | 0.0442 |  | - |  | - |  | - |  |  |  |  |  |  |
|  |  |  |  |  |  |  |  |  |  |  |  |  |  |  |  |  |  |  |  |  |  |  |  |  |
|  |  | RTG-2 | | | | | | | | | | | | | | | | | | | | | | |
|  |  | TN68[H] | | | | |  | TN80[H] | | | | |  | TN470[M] | | | | |  | TN480[L] | | | | |
|  |  | Intr |  | Extr |  | Prog |  | Intr |  | Extr |  | Prog |  | Intr |  | Extr |  | Prog |  | Intr |  | Extr |  | Prog |
| TN68[H] | Intr | - |  | 0.7796 |  | 0.7078 |  | 0.2039 |  | - |  | - |  | 0.9269 |  | - |  | - |  | 0.9373 |  | - |  | - |
|  | Extr | - |  | - |  | 0.7555 |  | - |  | 0.7087 |  | - |  | - |  | 0.9745 |  | - |  | - |  | 0.9754 |  | - |
|  | Prog | - |  | - |  | - |  | - |  | - |  | 0.9705 |  | - |  | - |  | 0.9209 |  | - |  | - |  | 0.966 |
| TN80[H] | Intr | - |  | - |  | - |  | - |  | 0.8810 |  | 0.9798 |  | 0.5023* |  | - |  | - |  | 0.2579* |  | - |  | - |
|  | Extr | - |  | - |  | - |  | - |  | - |  | 0.9383 |  | - |  | 0.8254 |  | - |  | - |  | 0.8074 |  | - |
|  | Prog | - |  | - |  | - |  | - |  | - |  | - |  | - |  | - |  | 0.9450 |  | - |  | - |  | 0.9041 |
| TN470[M] | Intr | - |  | - |  | - |  | - |  | - |  | - |  | - |  | 0.7086 |  | 0.5102 |  | 0.9555 |  | - |  | - |
|  | Extr | - |  | - |  | - |  | - |  | - |  | - |  | - |  | - |  | 0.6567 |  | - |  | 0.9825 |  | - |
|  | Prog | - |  | - |  | - |  | - |  | - |  | - |  | - |  | - |  | - |  | - |  | - |  | 0.8534 |
| TN480[L] | Intr | - |  | - |  | - |  | - |  | - |  | - |  | - |  | - |  | - |  | - |  | 0.7651 |  | 0.8805 |
|  | Extr | - |  | - |  | - |  | - |  | - |  | - |  | - |  | - |  | - |  | - |  | - |  | 0.8599 |
|  | Prog | - |  | - |  | - |  | - |  | - |  | - |  | - |  | - |  | - |  | - |  | - |  | - |
|  |  |  |  |  |  |  |  |  |  |  |  |  |  |  |  |  |  |  |  |  |  |  |  |  |
|  |  |  |  |  |  |  |  |  |  |  |  |  |  |  |  |  |  |  |  |  |  |  |  |  |
|  |  |  |  |  |  |  |  |  |  |  |  |  |  |  |  |  |  |  |  |  |  |  |  |  |
| B.- Differences between replication curves (2 ways ANOVA; data are shown as P values) | | | | | | | | | | | | | | | | | | | | | | | | |
|  |  | EPC | | | | | | | | | | | | | | | | |  |  |  |  |  |  |
|  |  | TN68[H] | | | | |  | TN470[M] | | | | |  | TN480[L] | | | | |  |  |  |  |  |  |
|  |  | Intr |  | Extr |  | Prog |  | Intr |  | Extr |  | Prog |  | Intr |  | Extr |  | Prog |  |  |  |  |  |  |
| TN68[H] | Intr | - |  | 0.2250 |  | - |  | 0.1708 |  | - |  | - |  | 0.0029 |  | - |  | - |  |  |  |  |  |  |
|  | Extr | - |  | - |  | 0.0012 |  | - |  | 0.0026 |  | - |  | - |  | 0.0273 |  | - |  |  |  |  |  |  |
|  | Prog | - |  | - |  | - |  | - |  | - |  | 0.0005 |  | - |  | - |  | 0.0432 |  |  |  |  |  |  |
| TN470[M] | Intr | - |  | - |  | - |  | - |  | 0.0015 |  | - |  | 0.0001 |  | - |  | - |  |  |  |  |  |  |
|  | Extr | - |  | - |  | - |  | - |  | - |  | 0.4450 |  | - |  | 0.0035 |  | - |  |  |  |  |  |  |
|  | Prog | - |  | - |  | - |  | - |  | - |  | - |  | - |  | - |  | 0.0002 |  |  |  |  |  |  |
| TN480[L] | Intr | - |  | - |  | - |  | - |  | - |  | - |  | - |  | 0.0612 |  |  |  |  |  |  |  |  |
|  | Extr | - |  | - |  | - |  | - |  | - |  | - |  | - |  | - |  | 0.0122 |  |  |  |  |  |  |
|  | Prog | - |  | - |  | - |  | - |  | - |  | - |  | - |  | - |  | - |  |  |  |  |  |  |
|  |  |  |  |  |  |  |  |  |  |  |  |  |  |  |  |  |  |  |  |  |  |  |  |  |
|  |  |  |  |  |  |  |  |  |  |  |  |  |  |  |  |  |  |  |  |  |  |  |  |  |
|  |  |  |  |  |  |  |  |  |  |  |  |  |  |  |  |  |  |  |  |  |  |  |  |  |
|  |  | RTG-2 | | | | | | | | | | | | | | | | | | | | | | |
|  |  | TN68[H] | | | | |  | TN80[H] | | | | |  | TN470[M] | | | | |  | TN480[L] | | | | |
|  |  | Intr |  | Extr |  | Prog |  | Intr |  | Extr |  | Prog |  | Intr |  | Extr |  | Prog |  | Intr |  | Extr |  | Prog |
| TN68[H] | Intr | - |  | 0.1127 |  | - |  | <0.0001 |  | - |  | - |  | 0.2129 |  | - |  | - |  | 0.9273 |  | - |  | - |
|  | Extr | - |  | - |  | 0.0073 |  | - |  | <0.0001 |  | - |  | - |  | 0.0008 |  | - |  | - |  | 0.0014 |  | - |
|  | Prog | - |  | - |  | - |  | - |  | - |  | 0.0002 |  | - |  | - |  | 0.0042 |  | - |  | - |  | 0.0166 |
| TN80[H] | Intr | - |  | - |  | - |  | - |  | 0.0148 |  | - |  | <0.0001 |  | - |  | - |  | <0.0001 |  | - |  | - |
|  | Extr | - |  | - |  | - |  | - |  | - |  | 0.0213 |  | - |  | <0.0001 |  | - |  | - |  | <0.0001 |  | - |
|  | Prog | - |  | - |  | - |  | - |  | - |  | - |  | - |  | - |  | <0.0001 |  | - |  | - |  | <0.0001 |
| TN470[M] | Intr | - |  | - |  | - |  | - |  | - |  | - |  | - |  | 0.6054 |  | - |  | 0.0602 |  | - |  | - |
|  | Extr | - |  | - |  | - |  | - |  | - |  | - |  | - |  | - |  | <0.0001 |  | - |  | 0.7984 |  | - |
|  | Prog | - |  | - |  | - |  | - |  | - |  | - |  | - |  | - |  | - |  | - |  | - |  | 0.0002 |
| TN480[L] | Intr | - |  | - |  | - |  | - |  | - |  | - |  | - |  | - |  | - |  | - |  | 0.0271 |  | - |
|  | Extr | - |  | - |  | - |  | - |  | - |  | - |  | - |  | - |  | - |  | - |  | - |  | 0.0335 |
|  | Prog | - |  | - |  | - |  | - |  | - |  | - |  | - |  | - |  | - |  | - |  | - |  | - |

| C.-Differences between replication curves (Average difference between time points titers) | | | | | | | | | | | | | | | | | | | | | | | | |
| --- | --- | --- | --- | --- | --- | --- | --- | --- | --- | --- | --- | --- | --- | --- | --- | --- | --- | --- | --- | --- | --- | --- | --- | --- |
|  |  | EPC | | | | | | | | | | | | | | | | |  |  |  |  |  |  |
|  |  | TN68[H] | | | | |  | TN470[M] | | | | |  | TN480[L] | | | | |  |  |  |  |  |  |
|  | AvTD SD | Intr |  | Extr |  | Prog |  | Intr |  | Extr |  | Prog |  | Intr |  | Extr |  | Prog |  |  |  |  |  |  |
| TN68[H] | Intr | - |  | 0.57 |  | 0.54 |  | 0.35 |  | - |  | - |  | 1.03* |  | - |  | - |  |  |  |  |  |  |
|  | Extr | 0.70 |  | - |  | 0.40 |  | - |  | 0.35 |  | - |  | - |  | 0.46 |  | - |  |  |  |  |  |  |
|  | Prog | 0.42 |  | 0.33 |  | - |  | - |  | - |  | 0.79 |  | - |  | - |  | 0.44 |  |  |  |  |  |  |
| TN470[M] | Intr | 0.23 |  | - |  | - |  | - |  | 0.09 |  | 1.06* |  | 0.74 |  | - |  | - |  |  |  |  |  |  |
|  | Extr | - |  | 0.29 |  | - |  | 0.06 |  | - |  | 0.97 |  | - |  | 0.79 |  | - |  |  |  |  |  |  |
|  | Prog | - |  | - |  | 0.60 |  | 0.27 |  | 0.22 |  | - |  | - |  | - |  | 1.17* |  |  |  |  |  |  |
| TN480[L] | Intr | 0.76 |  | - |  | - |  | 0.73 |  | - |  | - |  | - |  | 0.15 |  | 0.36 |  |  |  |  |  |  |
|  | Extr | - |  | 0.38 |  | - |  | - |  | 0.66 |  | - |  | 0.18 |  | - |  | 0.57 |  |  |  |  |  |  |
|  | Prog | - |  | - |  | 0.31 |  | - |  | - |  | 0.89 |  | 0.31 |  | 0.62 |  | - |  |  |  |  |  |  |
|  |  |  |  |  |  |  |  |  |  |  |  |  |  |  |  |  |  |  |  |  |  |  |  |  |
|  |  |  |  |  |  |  |  |  |  |  |  |  |  |  |  |  |  |  |  |  |  |  |  |  |
|  |  | RTG-2 | | | | | | | | | | | | | | | | | | | | | | |
|  |  | TN68[H] | | | | |  | TN80[H] | | | | |  | TN470[M] | | | | |  | TN480[L] | | | | |
|  | AvTD SD | Intr |  | Extr |  | Prog |  | Intr |  | Extr |  | Prog |  | Intr |  | Extr |  | Prog |  | Intr |  | Extr |  | Prog |
| TN68[H] | Intr | - |  | 0.75 |  | 1.19* |  | 3.12* |  | - |  | - |  | 0.44 |  | - |  | - |  | 0.46 |  | - |  | - |
|  | Extr | 0.40 |  | - |  | 0.99 |  | - |  | 3.25* |  | - |  | - |  | 0.48 |  | - |  | - |  | 0.57 |  | - |
|  | Prog | 0.72 |  | 0.52 |  | - |  | - |  | - |  | 2.29* |  | - |  | - |  | 0.76 |  | - |  | - |  | 0.75 |
| TN80[H] | Intr | 1.97* |  | - |  | - |  | - |  | 0.63 |  | 0.69 |  | 2.93* |  | - |  | - |  | 2.89* |  | - |  | - |
|  | Extr | - |  | 1.58* |  | - |  | 0.23 |  | - |  | 0.47 |  | - |  | 2.80* |  | - |  | - |  | 2.82* |  | - |
|  | Prog | - |  | - |  | 1.42 |  | 0.51 |  | 0.24 |  | - |  | - |  | - |  | 1.73* |  | - |  | - |  | 2.91* |
| TN470[M] | Intr | 0.30 |  | - |  | - |  | 1.64 |  | - |  | - |  | - |  | 0.79 |  | 1.73* |  | 0.35 |  | - |  | - |
|  | Extr | - |  | 0.47 |  | - |  | - |  | 1.33 |  | - |  | 0.44 |  | - |  | 1.41* |  | - |  | 0.36 |  | - |
|  | Prog | - |  | - |  | 0.59 |  | - |  | - |  | 1.22 |  | 1.13 |  | 0.89 |  | - |  | - |  | - |  | 1.45* |
| TN480[L] | Intr | 0.27 |  | - |  | - |  | 1.84 |  | - |  | - |  | 0.22 |  | - |  | - |  | - |  | 0.85 |  | 0.83 |
|  | Extr | - |  | 0.33 |  | - |  | - |  | 1.27 |  | - |  | - |  | 0.24 |  | - |  | 0.40 |  | - |  | 0.37 |
|  | Prog | - |  | - |  | 0.44 |  | - |  | - |  | 1.28 |  | -- |  | - |  | 0.92 |  | 0.40 |  | 0.29 |  | - |

A.- In the first part of the table, correlation between curves is given by the correlation coefficient values (r), being r=1 the maximum correlation between 2 curves; correlation is confirmed by P≤0.05; *no significant correlation values (**r** values with P>0.05). B.- In the second part of the table, the results of a SIDAK multiple comparison 2-way ANOVA test was employed (differences considered significant only for values of P≤0.01). C.- The third part shows the average differences among each time point between two curves (average differences higher 1 Log_10_ are considered significant and labelled with a *****). AvTD: Average titer differences (data in blue color; from 3 replicas); SD: Standard deviation.
