# Supplementary material for: Steps of the Replication Cycle of the Viral Haemorrhagic Septicaemia Virus (VHSV) Affecting Its Virulence on Fish
Source: Animals (Basel). 2020 Dec 1;10(12):2264. doi: 10.3390/ani10122264 (PMC7761041; doi:10.3390/ani10122264)
Supplement: Supplementary file 1 [file animals-10-02264-s001.zip › Supplementary items-wo Fig Legend-2/Supplementary Table 12-Corr coef and stat diffs between curves_Fr strains-vs5.docx]

Supplementary Table 12.- Correlation coefficient and statistical differences between replication curves: French strains

| A.-Correlation between replication curves | | | | | | | | | | | | | | | | | | | | | | | | | | | | | | | | | | | |
| --- | --- | --- | --- | --- | --- | --- | --- | --- | --- | --- | --- | --- | --- | --- | --- | --- | --- | --- | --- | --- | --- | --- | --- | --- | --- | --- | --- | --- | --- | --- | --- | --- | --- | --- | --- |
|  | EPC | | | | | | | | | | | | | | | | | | | | | | | | | | | | | | | | | | |
|  | WT[H] | | | | |  | DD224[L] | | | | |  | NV-R116Y[H] | | | | |  | NV-R116S[M] | | | | |  | N-K46[L] | | | | |  | NV_N[L] | | | | |
|  | Intr |  | Extr |  | Prog |  | Intr |  | Extr |  | Prog |  | Intr |  | Extr |  | Prog |  | Intr |  | Extr |  | Prog |  | Intr |  | Extr |  | Prog |  | Intr |  | Extr |  | Prog |
| WT[H] | |  |  |  |  |  |  |  |  |  |  |  |  |  |  |  |  |  |  |  |  |  |  |  |  |  |  |  |  |  |  |  |  |  |  |
| Intr | - |  | 0.8369 |  | 0.9822 |  | 0.9943 |  | - |  | - |  | 0.9917 |  | - |  | - |  | 0.9877 |  | - |  | - |  | 0.9388 |  | - |  | - |  | 0.5134* |  | - |  | - |
| Extr | - |  | - |  | 0.8709 |  | - |  | 0.9700 |  | - |  | - |  | 0.9874 |  | - |  | - |  | 0.9928 |  | - |  | - |  | 0.9229 |  | - |  | - |  | 0.8669 |  | - |
| Prog | - |  | - |  | - |  | - |  | - |  | 0.9945 |  | - |  | - |  | 0.9958 |  | - |  | - |  | 0.9841 |  | - |  | - |  | 0.7984 |  | - |  | - |  | 0.7416 |
| DD[L] | |  |  |  |  |  |  |  |  |  |  |  |  |  |  |  |  |  |  |  |  |  |  |  |  |  |  |  |  |  |  |  |  |  |  |
| Intr | - |  | - |  | - |  | - |  | 0.8770 |  | 0.9931 |  | 0.9966 |  | - |  | - |  | 0.9921 |  | - |  | - |  | 0.9065 |  | - |  | - |  | 0.5602* |  | - |  | - |
| Extr | - |  | - |  | - |  | - |  | - |  | 0.4900* |  | - |  | 0.9943 |  | - |  | - |  | 0.9974 |  | - |  | - |  | 0.9122 |  | - |  | - |  | 0.7710 |  | - |
| Prog | - |  | - |  | - |  | - |  | - |  | - |  | - |  | - |  | 0.9905 |  | - |  | - |  | 0.9767 |  | - |  | - |  | 0.8365 |  | - |  | - |  | 0.7851 |
| 116Y[H] | |  |  |  |  |  |  |  |  |  |  |  |  |  |  |  |  |  |  |  |  |  |  |  |  |  |  |  |  |  |  |  |  |  |  |
| Intr | - |  | - |  | - |  | - |  | - |  | - |  | - |  | 0.8045 |  | 0.9857 |  | 0.9971 |  | - |  | - |  | 0.9166 |  | - |  | - |  | 0.5317* |  | - |  | - |
| Extr | - |  | - |  | - |  | - |  | - |  | - |  | - |  | - |  | 0.4727* |  | - |  | 0.9865 |  | - |  | - |  | 0.8716 |  | - |  | - |  | 0.7370 |  | - |
| Prog | - |  | - |  | - |  | - |  | - |  | - |  | - |  | - |  | - |  | - |  | - |  | 0.9783 |  | - |  | - |  | 0.7813 |  | - |  | - |  | 0.7500 |
| 116S[M] | |  |  |  |  |  |  |  |  |  |  |  |  |  |  |  |  |  |  |  |  |  |  |  |  |  |  |  |  |  |  |  |  |  |  |
| Intr | - |  | - |  | - |  | - |  | - |  | - |  | - |  | - |  | - |  | - |  | 0.8398 |  | 0.9891 |  | 0.9095 |  | - |  | - |  | 0.5601* |  | - |  | - |
| Extr | - |  | - |  | - |  | - |  | - |  | - |  | - |  | - |  | - |  | - |  | - |  | 0.5138* |  | - |  | 0.8833 |  | - |  | - |  | 0.7503 |  | - |
| Prog | - |  | - |  | - |  | - |  | - |  | - |  | - |  | - |  | - |  | - |  | - |  | - |  | - |  | - |  | 0.7972 |  | - |  | - |  | 0.7199 |
| N-K46[L] | |  |  |  |  |  |  |  |  |  |  |  |  |  |  |  |  |  |  |  |  |  |  |  |  |  |  |  |  |  |  |  |  |  |  |
| Intr | - |  | - |  | - |  | - |  | - |  | - |  | - |  | - |  | - |  | - |  | - |  | - |  | - |  | 0.5788* |  | 0.8996 |  | 0.2844* |  | - |  | - |
| Extr | - |  | - |  | - |  | - |  | - |  | - |  | - |  | - |  | - |  | - |  | - |  | - |  | - |  | - |  | 0.3380* |  | - |  | 0.9205 |  | - |
| Prog | - |  | - |  | - |  | - |  | - |  | - |  | - |  | - |  | - |  | - |  | - |  | - |  | - |  | - |  | - |  | - |  | - |  | 0.6686* |
| NV_N[L] | |  |  |  |  |  |  |  |  |  |  |  |  |  |  |  |  |  |  |  |  |  |  |  |  |  |  |  |  |  |  |  |  |  |  |
| Intr | - |  | - |  | - |  | - |  | - |  | - |  | - |  | - |  | - |  | - |  | - |  | - |  | - |  | - |  | - |  | - |  | 0.9359 |  | 0.6395* |
| Extr | - |  | - |  | - |  | - |  | - |  | - |  | - |  | - |  | - |  | - |  | - |  | - |  | - |  | - |  | - |  | - |  | - |  | 0.9153 |
| Prog | - |  | - |  | - |  | - |  | - |  | - |  | - |  | - |  | - |  | - |  | - |  | - |  | - |  | - |  | - |  | - |  | - |  | - |
|  |  |  |  |  |  |  |  |  |  |  |  |  |  |  |  |  |  |  |  |  |  |  |  |  |  |  |  |  |  |  |  |  |  |  |  |
| B.-Differences between replication curves (2 ways ANOVA) | | | | | | | | | | | | | | | | | | | | | | | | | | | | | | | | | | | |
|  | EPC | | | | | | | | | | | | | | | | | | | | | | | | | | | | | | | | | | |
|  | WT[H] | | | | |  | DD224[L] | | | | |  | NV-R116Y[H] | | | | |  | NV-R116S[M] | | | | |  | N-K46[L] | | | | |  | NV_N[L] | | | | |
|  | Intr |  | Extr |  | Prog |  | Intr |  | Extr |  | Prog |  | Intr |  | Extr |  | Prog |  | Intr |  | Extr |  | Prog |  | Intr |  | Extr |  | Prog |  | Intr |  | Extr |  | Prog |
| WT[H] | |  |  |  |  |  |  |  |  |  |  |  |  |  |  |  |  |  |  |  |  |  |  |  |  |  |  |  |  |  |  |  |  |  |  |
| Intr | - |  | 0.0026 |  | - |  | 0.0065 |  | - |  | - |  | 0.0183 |  | - |  | - |  | <0.0001 |  | - |  | - |  | <0.0001 |  | - |  | - |  | 0.0005 |  | - |  | - |
| Extr | - |  | - |  | 0.0029 |  | - |  | 0.0026 |  | - |  | - |  | 0.0328 |  | - |  | - |  | 0.0069 |  | - |  | - |  | <0.0001 |  | - |  | - |  | <0.0001 |  | - |
| Prog | - |  | - |  | - |  | - |  | - |  | 0.0001 |  | - |  | - |  | 0.0090 |  | - |  | - |  | 0.1680 |  | - |  | - |  | <0.0001 |  | - |  | - |  | <0.0001 |
| DD[L] | |  |  |  |  |  |  |  |  |  |  |  |  |  |  |  |  |  |  |  |  |  |  |  |  |  |  |  |  |  |  |  |  |  |  |
| Intr | - |  | - |  | - |  | - |  | 0.0095 |  | - |  | 0.4587 |  | - |  | - |  | 0.0021 |  | - |  | - |  | <0.0001 |  | - |  | - |  | 0.0010 |  | - |  | - |
| Extr | - |  | - |  | - |  | - |  | - |  | 0.0005 |  | - |  | 0.124 |  | - |  | - |  | 0.0072 |  | - |  | - |  | <0.0001 |  | - |  | - |  | <0.0001 |  | - |
| Prog | - |  | - |  | - |  | - |  | - |  | - |  | - |  | - |  | 0.0002 |  | - |  | - |  | 0.0594 |  | - |  | - |  | <0.0001 |  | - |  | - |  | <0.0001 |
| 116Y[H] | |  |  |  |  |  |  |  |  |  |  |  |  |  |  |  |  |  |  |  |  |  |  |  |  |  |  |  |  |  |  |  |  |  |  |
| Intr | - |  | - |  | - |  | - |  | - |  | - |  | - |  | 0.0167 |  | - |  | 0.0435 |  | - |  | - |  | 0.0008 |  | - |  | - |  | 0.0015 |  | - |  | - |
| Extr | - |  | - |  | - |  | - |  | - |  | - |  | - |  | - |  | 0.7590 |  | - |  | 0.7430 |  | - |  | - |  | 0.0005 |  | - |  | - |  | 0.0001 |  | - |
| Prog | - |  | - |  | - |  | - |  | - |  | - |  | - |  | - |  | - |  | - |  | - |  | 0.0332 |  | - |  | - |  | <0.0001 |  | - |  | - |  | <0.0001 |
| 116S[M] | |  |  |  |  |  |  |  |  |  |  |  |  |  |  |  |  |  |  |  |  |  |  |  |  |  |  |  |  |  |  |  |  |  |  |
| Intr | - |  | - |  | - |  | - |  | - |  | - |  | - |  | - |  | - |  | - |  | <0.0001 |  | - |  | <0.0001 |  | - |  | - |  | 0.0021 |  | - |  | - |
| Extr | - |  | - |  | - |  | - |  | - |  | - |  | - |  | - |  | - |  | - |  | - |  | 0.0519 |  | - |  | <0.0001 |  | - |  | - |  | <0.0001 |  | - |
| Prog | - |  | - |  | - |  | - |  | - |  | - |  | - |  | - |  | - |  | - |  | - |  | - |  | - |  | - |  | <0.0001 |  | - |  | - |  | <0.0001 |
| N-K46[L] | |  |  |  |  |  |  |  |  |  |  |  |  |  |  |  |  |  |  |  |  |  |  |  |  |  |  |  |  |  |  |  |  |  |  |
| Intr | - |  | - |  | - |  | - |  | - |  | - |  | - |  | - |  | - |  | - |  | - |  | - |  | - |  | 0.0298 |  | - |  | 0.0143 |  | - |  | - |
| Extr | - |  | - |  | - |  | - |  | - |  | - |  | - |  | - |  | - |  | - |  | - |  | - |  | - |  | - |  | <0.0001 |  | - |  | 0.0006 |  | - |
| Prog | - |  | - |  | - |  | - |  | - |  | - |  | - |  | - |  | - |  | - |  | - |  | - |  | - |  | - |  | - |  | - |  | - |  | <0.0001 |
| NV_N[L] | |  |  |  |  |  |  |  |  |  |  |  |  |  |  |  |  |  |  |  |  |  |  |  |  |  |  |  |  |  |  |  |  |  |  |
| Intr | - |  | - |  | - |  | - |  | - |  | - |  | - |  | - |  | - |  | - |  | - |  | - |  | - |  | - |  | - |  | - |  | 0.0694 |  |  |
| Extr | - |  | - |  | - |  | - |  | - |  | - |  | - |  | - |  | - |  | - |  | - |  | - |  | - |  | - |  | - |  | - |  | - |  | 0.0001 |
| Prog | - |  | - |  | - |  | - |  | - |  | - |  | - |  | - |  | - |  | - |  | - |  | - |  | - |  | - |  | - |  | - |  | - |  | - |

| C.-Differences between replication curves (Average difference between time points titers) | | | | | | | | | | | | | | | | | | | | | | | | | | | | | | | | | | | |
| --- | --- | --- | --- | --- | --- | --- | --- | --- | --- | --- | --- | --- | --- | --- | --- | --- | --- | --- | --- | --- | --- | --- | --- | --- | --- | --- | --- | --- | --- | --- | --- | --- | --- | --- | --- |
|  | EPC | | | | | | | | | | | | | | | | | | | | | | | | | | | | | | | | | | |
|  | WT[H] | | | | |  | DD224[L] | | | | |  | NV-R116Y[H] | | | | |  | NV-R116S[M] | | | | |  | N-K46[L] | | | | |  | NV_N[L] | | | | |
| AvTD SD | Intr |  | Extr |  | Prog |  | Intr |  | Extr |  | Prog |  | Intr |  | Extr |  | Prog |  | Intr |  | Extr |  | Prog |  | Intr |  | Extr |  | Prog |  | Intr |  | Extr |  | Prog |
| WT[H] | |  |  |  |  |  |  |  |  |  |  |  |  |  |  |  |  |  |  |  |  |  |  |  |  |  |  |  |  |  |  |  |  |  |  |
| Intr | - |  | 1.38* |  | 0.65 |  | 0.34 |  | - |  | - |  | 0.38 |  | - |  | - |  | 0.67 |  | - |  | - |  | 1.32* |  | - |  | - |  | 2.77* |  | - |  | - |
| Extr | 0.92 |  | - |  | 1.28* |  | - |  | 0.45 |  | - |  | - |  | 0.50 |  | - |  | - |  | 0.59 |  | - |  | - |  | 1.63* |  | - |  | - |  | 2.76* |  | - |
| Prog | 0.23 |  | 1.26 |  | - |  | - |  | - |  | 0.53 |  | - |  | - |  | 0.32 |  | - |  | - |  | 0.43 |  | - |  | - |  | 2.59* |  | - |  | - |  | 3.75* |
| DD[L] | |  |  |  |  |  |  |  |  |  |  |  |  |  |  |  |  |  |  |  |  |  |  |  |  |  |  |  |  |  |  |  |  |  |  |
| Intr | 0.24 |  | - |  | - |  | - |  | 0.86 |  | 0.61 |  | 0.25 |  | - |  | - |  | 0.48 |  | - |  | - |  | 1.41* |  | - |  | - |  | 2.52* |  | - |  | - |
| Extr | - |  | 0.32 |  | - |  | 0.92 |  | - |  | 1.44* |  | - |  | 0.32 |  | - |  | - |  | 0.21 |  | - |  | - |  | 1.39* |  | - |  | - |  | 2.31* |  | - |
| Prog | - |  | - |  | 0.30 |  | 0.38 |  | 0.98 |  | - |  | - |  | - |  | 0.70 |  | - |  | - |  | 0.59 |  | - |  | - |  | 2.10* |  | - |  | - |  | 3.38* |
| 116Y[H] | |  |  |  |  |  |  |  |  |  |  |  |  |  |  |  |  |  |  |  |  |  |  |  |  |  |  |  |  |  |  |  |  |  |  |
| Intr | 0.33 |  | - |  | - |  | 0.11 |  | - |  | - |  | - |  | 0.75 |  | 0.44 |  | 0.33 |  | - |  | - |  | 1.35* |  | - |  | - |  | 2.52* |  | - |  | - |
| Extr | - |  | 0.55 |  | - |  | - |  | 0.21 |  | - |  | 1.10 |  | - |  | 1.08* |  | - |  | 0.51 |  | - |  | - |  | 1.55* |  | - |  | - |  | 2.41* |  | - |
| Prog | - |  | - |  | 0.17 |  | - |  | - |  | 0.46 |  | 0.28 |  | 1.41 |  | - |  | - |  | 0.00 |  | 0.70 |  | - |  | - |  | 2.70* |  | - |  | - |  | 3.94* |
| 116S[M] | |  |  |  |  |  |  |  |  |  |  |  |  |  |  |  |  |  |  |  |  |  |  |  |  |  |  |  |  |  |  |  |  |  |  |
| Intr | 0.34 |  | - |  | - |  | 0.32 |  | - |  | - |  | 0.28 |  | - |  | - |  | - |  | 0.96 |  | 0.37 |  | 1.17* |  | - |  | - |  | 2.41* |  | - |  | - |
| Extr | - |  | 0.27 |  | - |  | - |  | 0.15 |  | - |  | - |  | 0.26 |  | - |  | 0.81 |  | - |  | 0.86 |  | - |  | 5.25* |  | - |  | - |  | 2.66* |  | - |
| Prog | - |  | - |  | 0.45 |  | - |  | - |  | 0.44 |  | - |  | - |  | 0.39 |  | 0.30 |  | 1.13 |  | - |  | - |  | - |  | 2.42* |  | - |  | - |  | 3.72* |
| N-K46[L] | |  |  |  |  |  |  |  |  |  |  |  |  |  |  |  |  |  |  |  |  |  |  |  |  |  |  |  |  |  |  |  |  |  |  |
| Intr | 0.90 |  | - |  | - |  | 1.01 |  | - |  | - |  | 0.91 |  | - |  | - |  | 0.76 |  | - |  | - |  | - |  | 1.73* |  | 1.87* |  | 2.50* |  | - |  | - |
| Extr | - |  | 1.20 |  | - |  | - |  | 0.91 |  | - |  | - |  | 1.03 |  | - |  | - |  | 1.91 |  | - |  | 1.28 |  | - |  | 2.87* |  | - |  | 1.26* |  | - |
| Prog | - |  | - |  | 1.07 |  | - |  | - |  | 0.69 |  | - |  | - |  | 1.23 |  | - |  | - |  | 1.40 |  | 0.64 |  | 1.48 |  | - |  | - |  | - |  | 2.30* |
| NV_N[L] | |  |  |  |  |  |  |  |  |  |  |  |  |  |  |  |  |  |  |  |  |  |  |  |  |  |  |  |  |  |  |  |  |  |  |
| Intr | 1.58 |  | - |  | - |  | 1.57 |  | - |  | - |  | 1,66 |  | - |  | - |  | 1.47 |  | - |  | - |  | 2.47 |  | - |  | - |  | - |  | 0.49 |  | 2.6*3 |
| Extr | - |  | 2.71 |  | - |  | - |  | 2.38 |  | - |  | - |  | 2.70 |  | - |  | - |  | 2.34 |  | - |  | - |  | 1.14 |  | - |  | 0.64 |  | - |  | 2.19* |
| Prog | - |  | - |  | 1.30 |  | - |  | - |  | 1.41 |  | - |  | - |  | 1.51 |  | - |  | - |  | 1.35 |  | - |  | - |  | 1.84 |  | 0.19 |  | 0.52 |  | - |

A.- In the first part of the table, correlation between curves is given by the correlation coefficient values (**r**), being **r**=1 the maximum correlation between 2 curves; correlation is confirmed by P≤0.05; *no significant correlation values (**r** values with P>0.05). B.- In the second part of the table, the results of a SIDAK multiple comparison 2-way ANOVA test was employed (differences considered significant only for values of P≤0.01). C.- The third part shows the average differences of titer in each time point between two curves (average differences higher than 1 Log_10_ are considered significant and labelled with a *****). AvTD: Average titer differences (data in blue color; from 3 replicas); SD: Standard deviation.
