# Supplementary material for: Steps of the Replication Cycle of the Viral Haemorrhagic Septicaemia Virus (VHSV) Affecting Its Virulence on Fish
Source: Animals (Basel). 2020 Dec 1;10(12):2264. doi: 10.3390/ani10122264 (PMC7761041; doi:10.3390/ani10122264)
Supplement: Supplementary file 1 [file animals-10-02264-s001.zip › Supplementary items-wo Fig Legend-2/Supplementary Table 11-Corr coef and stat diffs between curves_DK strains-vs3.pdf]

Supplementary Table 11.- Correlation coefficient and statistical differences between replication curves: Danish strains

A.-Correlation between replication curves

		BF-2								
		DK3592[H]			DK-F1[V]			DK1p8[L]		
		Intr	Extr	Prog	Intr	Extr	Prog	Intr	Extr	Prog
DK3592[H]	Intr	-	-	-	-	-	-	-	-	-
	Extr	-	-	-	-	-	-	-	-	-
	Prog	-	-	-	-	-	0.9816	-	-	0.9968
DK-F1[V]	Intr	-	-	-	-	-	-	-	-	-
	Extr	-	-	-	-	-	-	-	-	-
	Prog	-	-	-	-	-	-	-	-	0.9819
DK1p8[L]	Intr	-	-	-	-	-	-	-	-	-
	Extr	-	-	-	-	-	-	-	-	-
	Prog	-	-	-	-	-	-	-	-	-

		RTG-2								
		DK3592[H]			DK-F1[V]			DK1p8[L]		
		Intr	Extr	Prog	Intr	Extr	Prog	Intr	Extr	Prog
DK3592[H]	Intr	-	0.9783	0.9738	0.9736	-	-	0.9306	-	-
	Extr	-	-	0.9315	-	0.9453	-	-	0.8988	-
	Prog	-	-	-	-	-	0.9023	-	-	0.9070
DK-F1[V]	Intr	-	-	-	-	0.9780	0.9322	0.8710	-	-
	Extr	-	-	-	-	-	0.9483	-	0.7832	-
	Prog	-	-	-	-	-	-	-	-	0.7141
DK1p8[L]	Intr	-	-	-	-	-	-	-	0.9219	0.9700
	Extr	-	-	-	-	-	-	-	-	0.8506
	Prog	-	-	-	-	-	-	-	-	-

B.-Differences between replication curves (2 ways ANOVA; data are shown as P values)

		BF-2								
		DK3592[H]			DK-F1[V]			DK1p8[L]		
		Intr	Extr	Prog	Intr	Extr	Prog	Intr	Extr	Prog
DK3592[H]	Intr	-	-	-	-	-	-	-	-	-
	Extr	-	-	-	-	-	-	-	-	-
	Prog	-	-	-	-	-	0.0001	-	-	0.0037
DK-F1[V]	Intr	-	-	-	-	-	-	-	-	-
	Extr	-	-	-	-	-	-	-	-	-
	Prog	-	-	-	-	-	-	-	-	0.0001
DK1p8[L]	Intr	-	-	-	-	-	-	-	-	-
	Extr	-	-	-	-	-	-	-	-	-
	Prog	-	-	-	-	-	-	-	-	-

		RTG-2								
		DK3592[H]			DK-F1[V]			DK1p8[L]		
		Intr	Extr	Prog	Intr	Extr	Prog	Intr	Extr	Prog
DK3592[H]	Intr	-	0.0684	0.1002	0.5573	-	-	0.0204	-	-
	Extr	-	-	0.0030	-	0.0138	-	-	<0.0001	-
	Prog	-	-	-	-	-	0.0004	-	-	0.0001
DK-F1[V]	Intr	-	-	-	-	0.3439	0.2471	0.0017	-	-
	Extr	-	-	-	-	-	0.0009	-	0.0005	-
	Prog	-	-	-	-	-	-	-	-	0.0688
DK1p8[L]	Intr	-	-	-	-	-	-	-	0.0063	0.0478
	Extr	-	-	-	-	-	-	-	-	0.0257
	Prog	-	-	-	-	-	-	-	-	-

C.-Differences between replication curves (Average difference between time points titers)

		BF-2								
		DK3592[H]			DK-F1[V]			DK1p8[L]		
	AvTD / SD	Intr	Extr	Prog	Intr	Extr	Prog	Intr	Extr	Prog
DK3592[H]	Intr	-	-	-	-	-	-	-	-	-
	Extr	-	-	-	-	-	-	-	-	-
	Prog	-	-	-	-	-	0.88	-	-	1.63
DK-F1[V]	Intr	-	-	-	-	-	-	-	-	-
	Extr	-	-	-	-	-	-	-	-	-
	Prog	-	-	0.71	-	-	-	-	-	0.78
DK1p8[L]	Intr	-	-	-	-	-	-	-	-	-
	Extr	-	-	-	-	-	-	-	-	-
	Prog	-	-	0.36	-	-	0.74	-	-	-

		RTG-2								
		DK3592[H]			DK-F1[V]			DK1p8[L]		
	AvTD / SD	Intr	Extr	Prog	Intr	Extr	Prog	Intr	Extr	Prog
DK3592[H]	Intr	-	0.63	0.57	0.57	-	-	0.96	-	-
	Extr	0.47	-	0.80	-	1.05*	-	-	1.44*	-
	Prog	0.49	1.11	-	-	-	1.34*	-	-	1.05*
DK-F1[V]	Intr	0.54	-	-	-	0.69	1.00*	1.33*	-	-
	Extr	-	0.97	-	0.42	-	0.89	-	1.99*	-
	Prog	-	-	1.24	1.13	0.99	-	-	-	1.92*
DK1p8[L]	Intr	0.96	-	-	0.60	-	-	-	0.78	0.67
	Extr	-	0.88	-	-	1.00	-	0.98	-	0.91
	Prog	-	-	0.78	-	-	1.33	0.42	1.36	-

A.- In the first part of the table, correlation between curves is given by the correlation coefficient values (**r**), being **r**=1 the maximum correlation between 2 curves; correlation is confirmed by P≤0.05; *no significant correlation values (**r** values with P>0.05). B.- In the second part of the table, the results of a SIDAK multiple comparison 2-way ANOVA test was employed (differences considered significant only for values of P≤0.01). C.- The third part shows the average differences of titer in each time point between two curves (average differences higher than 1 Log_{10} are considered significant and labelled with a *). AvTD: Average titer differences (data in blue color; from 3 replicas); SD: Standard deviation.
