# Supplementary material for: Steps of the Replication Cycle of the Viral Haemorrhagic Septicaemia Virus (VHSV) Affecting Its Virulence on Fish
Source: Animals (Basel). 2020 Dec 1;10(12):2264. doi: 10.3390/ani10122264 (PMC7761041; doi:10.3390/ani10122264)
Supplement: Supplementary file 1 [file animals-10-02264-s001.zip › Supplementary items-wo Fig Legend-2/Supplementary Table 12-Corr coef and stat diffs between curves_Fr strains-vs5.pdf]

Supplementary Table 12.- Correlation coefficient and statistical differences between replication curves: French strains

A.-Correlation between replication curves

	EPC																	
	WT[H]			DD224[L]			NV-R116Y[H]			NV-R116S[M]			N-K46[L]			NV_N[L]		
	Intr	Extr	Prog	Intr	Extr	Prog	Intr	Extr	Prog	Intr	Extr	Prog	Intr	Extr	Prog	Intr	Extr	Prog
WT[H]																		
Intr	-	0.8369	0.9822	0.9943	-	-	0.9917	-	-	0.9877	-	-	0.9388	-	-	0.5134*	-	-
Extr	-	-	0.8709	-	0.9700	-	-	0.9874	-	-	0.9928	-	-	0.9229	-	-	0.8669	-
Prog	-	-	-	-	-	0.9945	-	-	0.9958	-	-	0.9841	-	-	0.7984	-	-	0.7416
DD[L]																		
Intr	-	-	-	-	0.8770	0.9931	0.9966	-	-	0.9921	-	-	0.9065	-	-	0.5602*	-	-
Extr	-	-	-	-	-	0.4900*	-	0.9943	-	-	0.9974	-	-	0.9122	-	-	0.7710	-
Prog	-	-	-	-	-	-	-	-	0.9905	-	-	0.9767	-	-	0.8365	-	-	0.7851
116Y[H]																		
Intr	-	-	-	-	-	-	-	0.8045	0.9857	0.9971	-	-	0.9166	-	-	0.5317*	-	-
Extr	-	-	-	-	-	-	-	-	0.4727*	-	0.9865	-	-	0.8716	-	-	0.7370	-
Prog	-	-	-	-	-	-	-	-	-	-	-	0.9783	-	-	0.7813	-	-	0.7500
116S[M]																		
Intr	-	-	-	-	-	-	-	-	-	-	0.8398	0.9891	0.9095	-	-	0.5601*	-	-
Extr	-	-	-	-	-	-	-	-	-	-	-	0.5138*	-	0.8833	-	-	0.7503	-
Prog	-	-	-	-	-	-	-	-	-	-	-	-	-	-	0.7972	-	-	0.7199
N-K46[L]																		
Intr	-	-	-	-	-	-	-	-	-	-	-	-	-	0.5788*	0.8996	0.2844*	-	-
Extr	-	-	-	-	-	-	-	-	-	-	-	-	-	-	0.3380*	-	0.9205	-
Prog	-	-	-	-	-	-	-	-	-	-	-	-	-	-	-	-	-	0.6686*
NV_N[L]																		
Intr	-	-	-	-	-	-	-	-	-	-	-	-	-	-	-	-	0.9359	0.6395*
Extr	-	-	-	-	-	-	-	-	-	-	-	-	-	-	-	-	-	0.9153
Prog	-	-	-	-	-	-	-	-	-	-	-	-	-	-	-	-	-	-

B.-Differences between replication curves (2 ways ANOVA)

	EPC																	
	WT[H]			DD224[L]			NV-R116Y[H]			NV-R116S[M]			N-K46[L]			NV_N[L]		
	Intr	Extr	Prog	Intr	Extr	Prog	Intr	Extr	Prog	Intr	Extr	Prog	Intr	Extr	Prog	Intr	Extr	Prog
**WT[H]**																		
Intr	-	0.0026	-	0.0065	-	-	0.0183	-	-	<0.0001	-	-	<0.0001	-	-	0.0005	-	-
Extr	-	-	0.0029	-	0.0026	-	-	0.0328	-	-	0.0069	-	-	<0.0001	-	-	<0.0001	-
Prog	-	-	-	-	-	0.0001	-	-	0.0090	-	-	0.1680	-	-	<0.0001	-	-	<0.0001
**DD[L]**																		
Intr	-	-	-	-	0.0095	-	0.4587	-	-	0.0021	-	-	<0.0001	-	-	0.0010	-	-
Extr	-	-	-	-	-	0.0005	-	0.124	-	-	0.0072	-	-	<0.0001	-	-	<0.0001	-
Prog	-	-	-	-	-	-	-	-	0.0002	-	-	0.0594	-	-	<0.0001	-	-	<0.0001
**116Y[H]**																		
Intr	-	-	-	-	-	-	-	0.0167	-	0.0435	-	-	0.0008	-	-	0.0015	-	-
Extr	-	-	-	-	-	-	-	-	0.7590	-	0.7430	-	-	0.0005	-	-	0.0001	-
Prog	-	-	-	-	-	-	-	-	-	-	-	0.0332	-	-	<0.0001	-	-	<0.0001
**116S[M]**																		
Intr	-	-	-	-	-	-	-	-	-	-	<0.0001	-	<0.0001	-	-	0.0021	-	-
Extr	-	-	-	-	-	-	-	-	-	-	-	0.0519	-	<0.0001	-	-	<0.0001	-
Prog	-	-	-	-	-	-	-	-	-	-	-	-	-	-	<0.0001	-	-	<0.0001
**N-K46[L]**																		
Intr	-	-	-	-	-	-	-	-	-	-	-	-	-	0.0298	-	0.0143	-	-
Extr	-	-	-	-	-	-	-	-	-	-	-	-	-	-	<0.0001	-	0.0006	-
Prog	-	-	-	-	-	-	-	-	-	-	-	-	-	-	-	-	-	<0.0001
**NV_N[L]**																		
Intr	-	-	-	-	-	-	-	-	-	-	-	-	-	-	-	-	0.0694	
Extr	-	-	-	-	-	-	-	-	-	-	-	-	-	-	-	-	-	0.0001
Prog	-	-	-	-	-	-	-	-	-	-	-	-	-	-	-	-	-	-

C.-Differences between replication curves (Average difference between time points titers)

AvTD / SD	EPC																	
	WT[H]			DD224[L]			NV-R116Y[H]			NV-R116S[M]			N-K46[L]			NV_N[L]		
	Intr	Extr	Prog	Intr	Extr	Prog	Intr	Extr	Prog	Intr	Extr	Prog	Intr	Extr	Prog	Intr	Extr	Prog
WT[H]																		
Intr	-	1.38*	0.65	0.34	-	-	0.38	-	-	0.67	-	-	1.32*	-	-	2.77*	-	-
Extr	0.92	-	1.28*	-	0.45	-	-	0.50	-	-	0.59	-	-	1.63*	-	-	2.76*	-
Prog	0.23	1.26	-	-	-	0.53	-	-	0.32	-	-	0.43	-	-	2.59*	-	-	3.75*
DD[L]																		
Intr	0.24	-	-	-	0.86	0.61	0.25	-	-	0.48	-	-	1.41*	-	-	2.52*	-	-
Extr	-	0.32	-	0.92	-	1.44*	-	0.32	-	-	0.21	-	-	1.39*	-	-	2.31*	-
Prog	-	-	0.30	0.38	0.98	-	-	-	0.70	-	-	0.59	-	-	2.10*	-	-	3.38*
116Y[H]																		
Intr	0.33	-	-	0.11	-	-	-	0.75	0.44	0.33	-	-	1.35*	-	-	2.52*	-	-
Extr	-	0.55	-	-	0.21	-	1.10	-	1.08*	-	0.51	-	-	1.55*	-	-	2.41*	-
Prog	-	-	0.17	-	-	0.46	0.28	1.41	-	-	0.00	0.70	-	-	2.70*	-	-	3.94*
116S[M]																		
Intr	0.34	-	-	0.32	-	-	0.28	-	-	-	0.96	0.37	1.17*	-	-	2.41*	-	-
Extr	-	0.27	-	-	0.15	-	-	0.26	-	0.81	-	0.86	-	5.25*	-	-	2.66*	-
Prog	-	-	0.45	-	-	0.44	-	-	0.39	0.30	1.13	-	-	-	2.42*	-	-	3.72*
N-K46[L]																		
Intr	0.90	-	-	1.01	-	-	0.91	-	-	0.76	-	-	-	1.73*	1.87*	2.50*	-	-
Extr	-	1.20	-	-	0.91	-	-	1.03	-	-	1.91	-	1.28	-	2.87*	-	1.26*	-
Prog	-	-	1.07	-	-	0.69	-	-	1.23	-	-	1.40	0.64	1.48	-	-	-	2.30*
NV_N[L]																		
Intr	1.58	-	-	1.57	-	-	1,66	-	-	1.47	-	-	2.47	-	-	-	0.49	2.6*3
Extr	-	2.71	-	-	2.38	-	-	2.70	-	-	2.34	-	-	1.14	-	0.64	-	2.19*
Prog	-	-	1.30	-	-	1.41	-	-	1.51	-	-	1.35	-	-	1.84	0.19	0.52	-

A.- In the first part of the table, correlation between curves is given by the correlation coefficient values (**r**), being **r**=1 the maximum correlation between 2 curves; correlation is confirmed by P≤0.05; *no significant correlation values (**r** values with P>0.05). B.- In the second part of the table, the results of a SIDAK multiple comparison 2-way ANOVA test was employed (differences considered significant only for values of P≤0.01). C.- The third part shows the average differences of titer in each time point between two curves (average differences higher than 1 Log_{10} are considered significant and labelled with a *). AvTD: Average titer differences (data in blue color; from 3 replicas); SD: Standard deviation.
