# Supplementary material for: Steps of the Replication Cycle of the Viral Haemorrhagic Septicaemia Virus (VHSV) Affecting Its Virulence on Fish
Source: Animals (Basel). 2020 Dec 1;10(12):2264. doi: 10.3390/ani10122264 (PMC7761041; doi:10.3390/ani10122264)
Supplement: Supplementary file 1 [file animals-10-02264-s001.zip › Supplementary items-wo Fig Legend-2/Supplementary Table 10-Corr coef and stat diffs between curves_It strains-vs5.pdf]

Supplementary Table 10.- Correlation coefficient and statistical differences between replication curves: Italian strains

A.- Correlation between replication curves

EPC

		TN68[H]			TN470[M]			TN480[L]		
		Intr	Extr	Prog	Intr	Extr	Prog	Intr	Extr	Prog
TN68[H]	Intr	-	0.4290*	0.9477	0.9931	-	-	0.9688	-	-
	Extr	-	-	0.6910*	-	0.9951	-	-	0.9903	-
	Prog	-	-	-	-	-	0.9385	-	-	0.9889
TN470[M]	Intr	-	-	-	-	0.4006*	0.8717	0.9715	-	-
	Extr	-	-	-	-	-	0.6164*	-	0.9741	-
	Prog	-	-	-	-	-	-	-	-	0.8881
TN480[L]	Intr	0.0066	-	-	0.0057	-	-	-	0.4428*	0.9350
	Extr	-	0.0012	-	-	0.0050	-	-	-	0.6920*
	Prog	-	-	0.0014	-	-	0.0442	-	-	-

RTG-2

		TN68[H]			TN80[H]			TN470[M]			TN480[L]		
		Intr	Extr	Prog	Intr	Extr	Prog	Intr	Extr	Prog	Intr	Extr	Prog
TN68[H]	Intr	-	0.7796	0.7078	0.2039	-	-	0.9269	-	-	0.9373	-	-
	Extr	-	-	0.7555	-	0.7087	-	-	0.9745	-	-	0.9754	-
	Prog	-	-	-	-	-	0.9705	-	-	0.9209	-	-	0.966
TN80[H]	Intr	-	-	-	-	0.8810	0.9798	0.5023*	-	-	0.2579*	-	-
	Extr	-	-	-	-	-	0.9383	-	0.8254	-	-	0.8074	-
	Prog	-	-	-	-	-	-	-	-	0.9450	-	-	0.9041
TN470[M]	Intr	-	-	-	-	-	-	-	0.7086	0.5102	0.9555	-	-
	Extr	-	-	-	-	-	-	-	-	0.6567	-	0.9825	-
	Prog	-	-	-	-	-	-	-	-	-	-	-	0.8534
TN480[L]	Intr	-	-	-	-	-	-	-	-	-	-	0.7651	0.8805
	Extr	-	-	-	-	-	-	-	-	-	-	-	0.8599
	Prog	-	-	-	-	-	-	-	-	-	-	-	-

B.- Differences between replication curves (2 ways ANOVA; data are shown as P values)

		EPC								
		TN68[H]			TN470[M]			TN480[L]		
		Intr	Extr	Prog	Intr	Extr	Prog	Intr	Extr	Prog
TN68[H]	Intr	-	0.2250	-	0.1708	-	-	0.0029	-	-
	Extr	-	-	0.0012	-	0.0026	-	-	0.0273	-
	Prog	-	-	-	-	-	0.0005	-	-	0.0432
TN470[M]	Intr	-	-	-	-	0.0015	-	0.0001	-	-
	Extr	-	-	-	-	-	0.4450	-	0.0035	-
	Prog	-	-	-	-	-	-	-	-	0.0002
TN480[L]	Intr	-	-	-	-	-	-	-	0.0612	
	Extr	-	-	-	-	-	-	-	-	0.0122
	Prog	-	-	-	-	-	-	-	-	-

		RTG-2											
		TN68[H]			TN80[H]			TN470[M]			TN480[L]		
		Intr	Extr	Prog	Intr	Extr	Prog	Intr	Extr	Prog	Intr	Extr	Prog
TN68[H]	Intr	-	0.1127	-	<0.0001	-	-	0.2129	-	-	0.9273	-	-
	Extr	-	-	0.0073	-	<0.0001	-	-	0.0008	-	-	0.0014	-
	Prog	-	-	-	-	-	0.0002	-	-	0.0042	-	-	0.0166
TN80[H]	Intr	-	-	-	-	0.0148	-	<0.0001	-	-	<0.0001	-	-
	Extr	-	-	-	-	-	0.0213	-	<0.0001	-	-	<0.0001	-
	Prog	-	-	-	-	-	-	-	-	<0.0001	-	-	<0.0001
TN470[M]	Intr	-	-	-	-	-	-	-	0.6054	-	0.0602	-	-
	Extr	-	-	-	-	-	-	-	-	<0.0001	-	0.7984	-
	Prog	-	-	-	-	-	-	-	-	-	-	-	0.0002
TN480[L]	Intr	-	-	-	-	-	-	-	-	-	-	0.0271	-
	Extr	-	-	-	-	-	-	-	-	-	-	-	0.0335
	Prog	-	-	-	-	-	-	-	-	-	-	-	-

C.-Differences between replication curves (Average difference between time points titers)

	AvTD / SD	EPC								
		TN68[H]			TN470[M]			TN480[L]		
		Intr	Extr	Prog	Intr	Extr	Prog	Intr	Extr	Prog
TN68[H]	Intr	-	0.57	0.54	0.35	-	-	1.03*	-	-
	Extr	0.70	-	0.40	-	0.35	-	-	0.46	-
	Prog	0.42	0.33	-	-	-	0.79	-	-	0.44
TN470[M]	Intr	0.23	-	-	-	0.09	1.06*	0.74	-	-
	Extr	-	0.29	-	0.06	-	0.97	-	0.79	-
	Prog	-	-	0.60	0.27	0.22	-	-	-	1.17*
TN480[L]	Intr	0.76	-	-	0.73	-	-	-	0.15	0.36
	Extr	-	0.38	-	-	0.66	-	0.18	-	0.57
	Prog	-	-	0.31	-	-	0.89	0.31	0.62	-

	AvTD / SD	RTG-2											
		TN68[H]			TN80[H]			TN470[M]			TN480[L]		
		Intr	Extr	Prog	Intr	Extr	Prog	Intr	Extr	Prog	Intr	Extr	Prog
TN68[H]	Intr	-	0.75	1.19*	3.12*	-	-	0.44	-	-	0.46	-	-
	Extr	0.40	-	0.99	-	3.25*	-	-	0.48	-	-	0.57	-
	Prog	0.72	0.52	-	-	-	2.29*	-	-	0.76	-	-	0.75
TN80[H]	Intr	1.97*	-	-	-	0.63	0.69	2.93*	-	-	2.89*	-	-
	Extr	-	1.58*	-	0.23	-	0.47	-	2.80*	-	-	2.82*	-
	Prog	-	-	1.42	0.51	0.24	-	-	-	1.73*	-	-	2.91*
TN470[M]	Intr	0.30	-	-	1.64	-	-	-	0.79	1.73*	0.35	-	-
	Extr	-	0.47	-	-	1.33	-	0.44	-	1.41*	-	0.36	-
	Prog	-	-	0.59	-	-	1.22	1.13	0.89	-	-	-	1.45*
TN480[L]	Intr	0.27	-	-	1.84	-	-	0.22	-	-	-	0.85	0.83
	Extr	-	0.33	-	-	1.27	-	-	0.24	-	0.40	-	0.37
	Prog	-	-	0.44	-	-	1.28	--	-	0.92	0.40	0.29	-

A.- In the first part of the table, correlation between curves is given by the correlation coefficient values (r), being r=1 the maximum correlation between 2 curves; correlation is confirmed by P≤0.05; *no significant correlation values (**r** values with P>0.05). B.- In the second part of the table, the results of a SIDAK multiple comparison 2-way ANOVA test was employed (differences considered significant only for values of P≤0.01). C.- The third part shows the average differences among each time point between two curves (average differences higher 1 Log_{10} are considered significant and labelled with a *). AvTD: Average titer differences (data in blue color; from 3 replicas); SD: Standard deviation.
