# Supplementary material for: Steps of the Replication Cycle of the Viral Haemorrhagic Septicaemia Virus (VHSV) Affecting Its Virulence on Fish
Source: Animals (Basel). 2020 Dec 1;10(12):2264. doi: 10.3390/ani10122264 (PMC7761041; doi:10.3390/ani10122264)
Supplement: Supplementary file 1 [file animals-10-02264-s001.zip › Supplementary items-wo Fig Legend-2/Supplementary Table 3-Adsorption Danish strains-vs6.pdf]

Supplementary Table 3.- Adsorption capacity of the Danish VHSV strains

Cell line: EPC

Adsorption time	Strain	Method	AAE[1]	RAE[2]	EOA[3]
30 min	DK3592B[H]	TCID	43.88±0.16	43.18±0.16	98.40±0.01
		qPCR	44.50±14.34	42.69±14.84	95.94±3.70
	DK-F1[V]	TCID	43.58±0.32	42.99±0.43	98.65±1.33
		qPCR	60.15±18.82	58.96±20.30	98.02±4.70
	DK1p8[L]	TCID	38.94±7.62	38.72±7.62	99.43±0.11
		qPCR	37.14±10.61	34.62±10.00	93.22±3.84

Cell line: RTG-2 (Repeat 1)

Adsorption time	Strain	Method	AAE	RAE	EOA
30 min	DK3592B[H]	TCID	43.18±0.46	40.75±2.68	94.37±5.78
		qPCR	47.76±6.57	45.98±7.88	95.32±4.41
	DK-F1[V]	TCID	53.55±13.98	52.38±14.20	97.81±1.29
		qPCR	44.93±7.85	44.82±9.71	96.02±5.01
	DK1p8[L]	TCID	43.77±6.49	39.45±7.91	90.13±10.14
		qPCR	43.79±11.15	43.48±1.79	97.10±1.25

Cell line: RTG-2 (Repeat 2)

Adsorption time	Strain	Method	AAE	RAE	EOA
30 min	DK3592B[H]	TCID	56.44±10.40	51.98±17.69	95.10±10.02
		qPCR	47.62±6.57	45.88±7.78	95.32±4.41
	DK-F1[V]	TCID	55.95±11.14	55.39±10.85	98.99±0.76
		qPCR	46.67±7.55	43.52±8.50	96.02±5.01
	DK1p8[L]	TCID	41.87±0.49	41.61±0.69	99.38±0.46
		qPCR	22.77±13.33	19.66±12.19	89.99±1.15

Cell line: RTG-2 (Average[4])

Adsorption time	Strain	Method	AAE	RAE	EOA
30 min	DK3592B[H]	TCID	49.81±5.43	45.37±10.19	94.74±7.9
		qPCR	47.76±5.88	45.93±7.83	95.32±4.41
	DK-F1[V]	TCID	55.75±12.56	55.89±12.53	98.40±1.03
		qPCR	44.93±7.02	44.17±9.15	96.02±5.01
	DK1p8[L]	TCID	41.82±3.54	40.53±4.30	94.76±5.30
		qPCR	33.28±15.91	31.57±6.99	93.55±1.20

Cell line: BF-2

Adsorption time	Strain	Method	AAE	RAE	EOA
30 min	DK3592B[H]	TCID	55.77±15.28	53.88±15.85	96.61±1.96
		qPCR	59.64±21.97	56.22±24.83	94.25±10.41
	DK-F1[V]	TCID	40.95±0.56	39.91±1.50	97.45±2.59
		qPCR	31.84±1.97	29.31±1.74	92.03±3.53
	DK1p8[L]	TCID	35.89±2.98	35.01±2.89	97.54±0.81
		qPCR	27.38±9.43	25.45±10.11	92.95±7.08

[1]Apparent adsorption efficacy: AAE=TAV (total adsorbed virus)/TIV (total inoculated virus) × 100; [2]Real adsorption efficacy: RAE=IAV (irreversibly adsorbed virus)/TIV × 100; [3]Efficiency of adsorption: EOA=IAV/TAV × 100; [4]Average from repeats 1 and 2.
