# Supplementary material for: Steps of the Replication Cycle of the Viral Haemorrhagic Septicaemia Virus (VHSV) Affecting Its Virulence on Fish
Source: Animals (Basel). 2020 Dec 1;10(12):2264. doi: 10.3390/ani10122264 (PMC7761041; doi:10.3390/ani10122264)
Supplement: Supplementary file 1 [file animals-10-02264-s001.zip › Supplementary items-wo Fig Legend-2/Supplementary Table 5-Adsorption differences-vs4.docx]

Supplementary Table 5.- **t** Test comparison of adsorption values

| A/Comparison of titers data (TCID_50_/ml) | | | | | | | | | | | | | | | | | |  |  |  |  |  |  | | | | | | | | | | | |
| --- | --- | --- | --- | --- | --- | --- | --- | --- | --- | --- | --- | --- | --- | --- | --- | --- | --- | --- | --- | --- | --- | --- | --- | --- | --- | --- | --- | --- | --- | --- | --- | --- | --- | --- |
| Spanish Strains | | | |  | Danish strains | | | | | |  | Italian strains | | | | | |  |  |  |  |  | French strains | | | | | | | | | | | |
|  | EPC-15min^1^ | | |  |  | EPC | | | | |  | EPC | | | | | |  |  |  |  |  | EPC^3^ | | | | | | | | | | | |
|  | H |  | [L] |  |  | H |  | V |  | L |  |  | H68 |  | H470 |  | L480 |  |  |  |  | | | Wt |  | DD |  | Y[H] |  | S[H] |  | NV_N[L] |  | NK46[L] |
| H | - |  | 0.0003 |  | H | - |  | 0.2390 |  | 0.1696 |  | H68 | - |  | 0.2542 |  | 0.0262 |  |  |  | Wt[H] | | | - |  | 0.0119 |  | 0.4288 |  | 0.3864 |  | 0.4636 |  | 0.0075 |
| L | - |  | - |  | V | - |  | - |  | 0.1890 |  | H470 | - |  | - |  | 0.0474 |  |  |  | DD[L] | | | - |  | - |  | 0.5507 |  | 0.4288 |  | 0.0383 |  | 0.0013 |
|  |  |  |  |  | L | - |  | - |  | - |  | L | - |  | - |  | - |  |  |  | Y[H] | | | - |  | - |  | - |  | 0.9513 |  | 0.2940 |  | 0.0940 |
| EPC-30min | | | |  | RTG-2^2^ | | | | | |  |  | RTG-15min | | | | |  |  |  | S[H] | | | - |  | - |  | - |  | - |  | 0.2614 |  | 0.0623 |
|  | H |  | [L] |  |  | H |  | V |  | L |  |  | H68 |  | H470 |  | L480 |  |  |  | NV_N[L] | | | - |  | - |  | - |  | - |  | - |  | 0.2807 |
| H | - |  | 0.0001 |  | H | - |  | 0.5367 |  | 0.3587 |  | H68 | - |  | 0.1616 |  | 0.0029 |  |  |  | NK46[L] | | | - |  | - |  | - |  | - |  | - |  | - |
| L | - |  | - |  | V | - |  | - |  | 0.3003 |  | H470 | - |  | - |  | 0.0240 |  |  |  |  | | |  |  |  |  |  |  |  |  |  |  |  |
|  |  |  |  |  | L | - |  | - |  | - |  | L | - |  | - |  | - |  |  |  |  | | |  |  |  |  |  |  |  |  |  |  |  |
| EPC-45min | | | |  | BF2 | | | | | |  | RTG-30 min | | | | | | | |  |  | | | | | | | | | |  |  |  |  |
|  | H |  | [L] |  |  | H |  | V |  | L |  |  | H68 |  | H80 |  | H470 |  | L480 |  |  | | |  |  |  |  |  |  |  |  |  |  |  |
| H | - |  | 0.0064 |  | H | - |  | 0.1686 |  | 0.0444 |  | H68 | - |  | 0.1068 |  | 0.0155 |  | <0.0001 |  |  | | |  |  |  |  |  |  |  |  |  |  |  |
| L | - |  | - |  | V | - |  | - |  | 0.0914 |  | H80 | - |  | - |  | 0.0724 |  | 0.0001 |  |  | | |  |  |  |  |  |  |  |  |  |  |  |
|  |  |  |  |  | L | - |  | - |  | - |  | H470 | - |  | - |  | - |  | 0.0017 |  |  | | |  |  |  |  |  |  |  |  |  |  |  |
|  |  |  |  |  |  |  |  |  |  |  |  | L480 | - |  | - |  | - |  | - |  |  | | |  |  |  |  |  |  |  |  |  |  |  |
| EPC-60min | | | |  |  | | | | | |  | RTG-45min | | | | | | |  |  |  | | |  |  |  |  |  |  |  |  |  |  |  |
|  | H |  | [L] |  |  |  |  |  |  |  |  |  | H68 |  | H470 |  | L480 |  |  |  |  | | |  |  |  |  |  |  |  |  |  |  |  |
| H | - |  | 0.0001 |  |  |  |  |  |  |  |  | H68 | - |  | 0.0153 |  | 0.0092 |  |  |  |  | | |  |  |  |  |  |  |  |  |  |  |  |
| L | - |  | - |  |  |  |  |  |  |  |  | H470 | - |  | - |  | 0.0706 |  |  |  |  | | |  |  |  |  |  |  |  |  |  |  |  |
|  |  |  |  |  |  |  |  |  |  |  |  | L480 | - |  | - |  | - |  |  |  |  | | |  |  |  |  |  |  |  |  |  |  |  |
| RTG-30min | | | |  |  |  |  |  |  |  |  | RTG-60min | | | | | |  |  |  |  | | |  |  |  |  |  |  |  |  |  |  |  |
|  | H |  | [L] |  |  |  |  |  |  |  |  |  | H68 |  | H470 |  | L480 |  |  |  |  | | |  |  |  |  |  |  |  |  |  |  |  |
| H | - |  | 0.0080 |  |  |  |  |  |  |  |  | H68 | - |  | 0.3530 |  | 0.0310 |  |  |  |  | | |  |  |  |  |  |  |  |  |  |  |  |
| L | - |  | - |  |  |  |  |  |  |  |  | H470 | - |  | - |  | 0.0002 |  |  |  |  | | |  |  |  |  |  |  |  |  |  |  |  |
|  |  |  |  |  |  |  |  |  |  |  |  | L480 | - |  | - |  | - |  |  |  |  | | |  |  |  |  |  |  |  |  |  |  |  |

| B/Comparison of RNA copies (determined by Rt-qPCR) | | | | | | | | | | | | | | | | | |  |  |  |  |  |  | | | | | | | | | | | |
| --- | --- | --- | --- | --- | --- | --- | --- | --- | --- | --- | --- | --- | --- | --- | --- | --- | --- | --- | --- | --- | --- | --- | --- | --- | --- | --- | --- | --- | --- | --- | --- | --- | --- | --- |
| Spanish Strains | | | |  | Danish strains | | | | | |  | Italian strains | | | | | |  |  |  |  |  | French strains | | | | | | | | | | | |
|  | EPC-15min^1^ | | |  |  | EPC | | | | |  | EPC | | | | | |  |  |  |  |  | EPC^3^ | | | | | | | | | | | |
|  | H |  | [L] |  |  | H |  | V |  | L |  |  | H68 |  | H470 |  | L480 |  |  |  |  | | | Wt |  | DD |  | Y[H] |  | S[H] |  | NV_N[L] |  | NK46[L] |
| H | - |  | 0.0770 |  | H | - |  | 0.3719 |  | 0.5852 |  | H68 | - |  | 0.3978 |  | 0.1238 |  |  |  | Wt[H] | | | - |  | 0.0560 |  | 0.5613 |  | 0.0588 |  | 0.4553 |  | 0.5399 |
| L | - |  | - |  | V | - |  | - |  | 0.1872 |  | H470 | - |  | - |  | 0.6057 |  |  |  | DD[L] | | | - |  | - |  | 0.1216 |  | 0.9225 |  | 0.0103 |  | 0.1125 |
|  |  |  |  |  | L | - |  | - |  | - |  | L | - |  | - |  | - |  |  |  | Y[H] | | | - |  | - |  | - |  | 0.1262 |  | 0.9884 |  | 0.9829 |
|  | | | |  | RTG-2^2^ | | | | | |  |  | RTG-15min | | | | |  |  |  | S[H] | | | - |  | - |  | - |  | - |  | 0.0174 |  | 0.1178 |
|  |  |  |  |  |  | H |  | V |  | L |  |  | H68 |  | H470 |  | L480 |  |  |  | NV_N[L] | | | - |  | - |  | - |  | - |  | - |  | 0.9892 |
|  |  |  |  |  | H | - |  | 0.6570 |  | 0.0965 |  | H68 | - |  | 0.4101 |  | 0.0162 |  |  |  | NK46[L] | | | - |  | - |  | - |  | - |  | - |  | - |
|  |  |  |  |  | V | - |  | - |  | 0.1780 |  | H470 | - |  | - |  | 0.0378 |  |  |  |  | | |  |  |  |  |  |  |  |  |  |  |  |
|  |  |  |  |  | L | - |  | - |  | - |  | L | - |  | - |  | - |  |  |  |  | | |  |  |  |  |  |  |  |  |  |  |  |
| EPC-45min | | | |  | BF2 | | | | | |  | RTG-30 min | | | | | | | |  |  | | | | | | | | | |  |  |  |  |
|  | H |  | [L] |  |  | H |  | V |  | L |  |  | H68 |  | H80 |  | H470 |  | L480 |  |  | | |  |  |  |  |  |  |  |  |  |  |  |
| H | - |  | 0.0256 |  | H | - |  | 0.1401 |  | 0.1128 |  | H68 | - |  | 0.7253 |  | 0.1881 |  | 0.0009 |  |  | | |  |  |  |  |  |  |  |  |  |  |  |
| L | - |  | - |  | V | - |  | - |  | 0.4585 |  | H80 | - |  | - |  | 0.3480 |  | 0.0054 |  |  | | |  |  |  |  |  |  |  |  |  |  |  |
|  |  |  |  |  | L | - |  | - |  | - |  | H470 | - |  | - |  | - |  | 0.0240 |  |  | | |  |  |  |  |  |  |  |  |  |  |  |
|  |  |  |  |  |  |  |  |  |  |  |  | L480 | - |  | - |  | - |  | - |  |  | | |  |  |  |  |  |  |  |  |  |  |  |
|  | | | |  |  | | | | | |  | RTG-45min | | | | | | |  |  |  | | |  |  |  |  |  |  |  |  |  |  |  |
|  |  |  |  |  |  |  |  |  |  |  |  |  | H68 |  | H470 |  | L480 |  |  |  |  | | |  |  |  |  |  |  |  |  |  |  |  |
|  |  |  |  |  |  |  |  |  |  |  |  | H68 | - |  | 0.1603 |  | 0.0150 |  |  |  |  | | |  |  |  |  |  |  |  |  |  |  |  |
|  |  |  |  |  |  |  |  |  |  |  |  | H470 | - |  | - |  | 0.1254 |  |  |  |  | | |  |  |  |  |  |  |  |  |  |  |  |
|  |  |  |  |  |  |  |  |  |  |  |  | L480 | - |  | - |  | - |  |  |  |  | | |  |  |  |  |  |  |  |  |  |  |  |
| RTG-30min | | | |  |  |  |  |  |  |  |  | RTG-60min | | | | | |  |  |  |  | | |  |  |  |  |  |  |  |  |  |  |  |
|  | H |  | [L] |  |  |  |  |  |  |  |  |  | H68 |  | H470 |  | L480 |  |  |  |  | | |  |  |  |  |  |  |  |  |  |  |  |
| H | - |  | 0.0080 |  |  |  |  |  |  |  |  | H68 | - |  | 0.1250 |  | 0.0158 |  |  |  |  | | |  |  |  |  |  |  |  |  |  |  |  |
| L | - |  | - |  |  |  |  |  |  |  |  | H470 | - |  | - |  | 0.0902 |  |  |  |  | | |  |  |  |  |  |  |  |  |  |  |  |
|  |  |  |  |  |  |  |  |  |  |  |  | L480 | - |  | - |  | - |  |  |  |  | | |  |  |  |  |  |  |  |  |  |  |  |

Names of strains are substituted by H or L (regarding their level of virulence), or by an abbreviated name when necessary. Data shown correspond to the P values; P≤0.05 are interpreted as significant differences. 1- Adsorption time. 2- Averaged from 2 repeats. 3- Averaged from 3 repeats.
