# Supplementary material for: Steps of the Replication Cycle of the Viral Haemorrhagic Septicaemia Virus (VHSV) Affecting Its Virulence on Fish
Source: Animals (Basel). 2020 Dec 1;10(12):2264. doi: 10.3390/ani10122264 (PMC7761041; doi:10.3390/ani10122264)
Supplement: Supplementary file 1 [file animals-10-02264-s001.zip › Supplementary items-wo Fig Legend-2/Supplementary Table 5-Adsorption differences-vs4.pdf]

Supplementary Table 5.- **t** Test comparison of adsorption values

A/Comparison of titers data ($TCID_{50}$/ml)

Spanish Strains

EPC-15min[1]

	H	[L]
H	-	0.0003
L	-	-

EPC-30min

	H	[L]
H	-	0.0001
L	-	-

EPC-45min

	H	[L]
H	-	0.0064
L	-	-

EPC-60min

	H	[L]
H	-	0.0001
L	-	-

RTG-30min

	H	[L]
H	-	0.0080
L	-	-

Danish strains

EPC

	H	V	L
H	-	0.2390	0.1696
V	-	-	0.1890
L	-	-	-

RTG-2[2]

	H	V	L
H	-	0.5367	0.3587
V	-	-	0.3003
L	-	-	-

BF2

	H	V	L
H	-	0.1686	0.0444
V	-	-	0.0914
L	-	-	-

Italian strains

EPC

	H68	H470	L480
H68	-	0.2542	0.0262
H470	-	-	0.0474
L	-	-	-

RTG-15min

	H68	H470	L480
H68	-	0.1616	0.0029
H470	-	-	0.0240
L	-	-	-

RTG-30 min

	H68	H80	H470	L480
H68	-	0.1068	0.0155	<0.0001
H80	-	-	0.0724	0.0001
H470	-	-	-	0.0017
L480	-	-	-	-

RTG-45min

	H68	H470	L480
H68	-	0.0153	0.0092
H470	-	-	0.0706
L480	-	-	-

RTG-60min

	H68	H470	L480
H68	-	0.3530	0.0310
H470	-	-	0.0002
L480	-	-	-

French strains

EPC[3]

	Wt	DD	Y[H]	S[H]	NV_N[L]	NK46[L]
Wt[H]	-	0.0119	0.4288	0.3864	0.4636	0.0075
DD[L]	-	-	0.5507	0.4288	0.0383	0.0013
Y[H]	-	-	-	0.9513	0.2940	0.0940
S[H]	-	-	-	-	0.2614	0.0623
NV_N[L]	-	-	-	-	-	0.2807
NK46[L]	-	-	-	-	-	-

B/Comparison of RNA copies (determined by Rt-qPCR)

Spanish Strains

EPC-15min[1]

	H	[L]
H	-	0.0770
L	-	-

EPC-45min

	H	[L]
H	-	0.0256
L	-	-

RTG-30min

	H	[L]
H	-	0.0080
L	-	-

Danish strains

EPC

	H	V	L
H	-	0.3719	0.5852
V	-	-	0.1872
L	-	-	-

RTG-2[2]

	H	V	L
H	-	0.6570	0.0965
V	-	-	0.1780
L	-	-	-

BF2

	H	V	L
H	-	0.1401	0.1128
V	-	-	0.4585
L	-	-	-

Italian strains

EPC

	H68	H470	L480
H68	-	0.3978	0.1238
H470	-	-	0.6057
L	-	-	-

RTG-15min

	H68	H470	L480
H68	-	0.4101	0.0162
H470	-	-	0.0378
L	-	-	-

RTG-30 min

	H68	H80	H470	L480
H68	-	0.7253	0.1881	0.0009
H80	-	-	0.3480	0.0054
H470	-	-	-	0.0240
L480	-	-	-	-

RTG-45min

	H68	H470	L480
H68	-	0.1603	0.0150
H470	-	-	0.1254
L480	-	-	-

RTG-60min

	H68	H470	L480
H68	-	0.1250	0.0158
H470	-	-	0.0902
L480	-	-	-

French strains

EPC[3]

	Wt	DD	Y[H]	S[H]	NV_N[L]	NK46[L]
Wt[H]	-	0.0560	0.5613	0.0588	0.4553	0.5399
DD[L]	-	-	0.1216	0.9225	0.0103	0.1125
Y[H]	-	-	-	0.1262	0.9884	0.9829
S[H]	-	-	-	-	0.0174	0.1178
NV_N[L]	-	-	-	-	-	0.9892
NK46[L]	-	-	-	-	-	-

Names of strains are substituted by H or L (regarding their level of virulence), or by an abbreviated name when necessary. Data shown correspond to the P values; P≤0.05 are interpreted as significant differences. 1- Adsorption time. 2- Averaged from 2 repeats. 3- Averaged from 3 repeats.
