# Supplementary material for: Steps of the Replication Cycle of the Viral Haemorrhagic Septicaemia Virus (VHSV) Affecting Its Virulence on Fish
Source: Animals (Basel). 2020 Dec 1;10(12):2264. doi: 10.3390/ani10122264 (PMC7761041; doi:10.3390/ani10122264)
Supplement: Supplementary file 1 [file animals-10-02264-s001.zip › Supplementary items-wo Fig Legend-2/Supplementary Table 7-Viral production_RNACopies-vs3.docx]

Supplementary Table 7- Viral production, quantified by qPCR (RNA copies)

| Spanish VHSV strains | | | | | | | | | | | | | | | | | | | | | | | | | | | | | | |
| --- | --- | --- | --- | --- | --- | --- | --- | --- | --- | --- | --- | --- | --- | --- | --- | --- | --- | --- | --- | --- | --- | --- | --- | --- | --- | --- | --- | --- | --- | --- |
| At the time of CPE^1^ | | | | | | | | | | | | |  | | Maximum viral production | | | | | | | | | | | | | | | |
| Line^2^ |  | MOI^3^ |  | Strain |  | Titer^4^ |  | | Time | |  | | | Line | |  | MOI |  | | Strain | |  | | Titer | |  | | Time | |  |
| EPC |  | 0.1 |  | Sm2897[H] |  | 1.9 x 10^8^ |  | | 3d | |  | | | EPC | |  | 0.1 |  | | Sm2897[H] | |  | | 1.0 x 10^8^  *8.02±0.05* | |  | | 6d | |  |
|  |  |  |  | DC1412[L] |  | 6.1 x 10^7^ |  | | 3d | |  | | |  | |  |  |  | | DC1412[L] | |  | | 1.1 x 10^8^  *8.00±0.17* | |  | | 7d | |  |
|  |  |  |  |  |  |  | |  | |  | |  | |  | |  |  |  |  | |  | |  | |  | |  | |  |  |
| RTG-2 |  | 0.01 |  | Sm2897[H] |  | 1.4 x 10^7^ |  | | 4-5d | |  | | | RTG-2 | |  | 0.1 |  | | Sm2897[H] | |  | | 3.4 x 10^7^  *7.52±0.14* | |  | | 6d | |  |
|  |  |  |  | DC1412[L] |  | 1.5 x 10^2^ |  | |  | |  | | |  | |  |  |  | | DC1412[L] | |  | | 5.2 x 10^7^  *7.62±0.39* | |  | | 3d | |  |
|  |  |  |  |  |  |  |  | |  | |  | | |  | |  |  |  | |  | |  | |  | |  | |  | |  |
|  |  | 0.1 |  | Sm2897[H] |  | 1.8 x 10^7^ |  | | 3d | |  | | |  | |  |  |  | |  | |  | |  | |  | |  | |  |
|  |  |  |  | DC1412[L] |  | 5.2 x 10^7^ |  | | 3d | |  | | |  | |  |  |  | |  | |  | |  | |  | |  | |  |
|  |  |  |  |  |  |  |  | |  | |  | | |  | |  |  |  | |  | |  | |  | |  | |  | |  |
|  |  | 1.0 |  | Sm2897[H] |  | 1.2 x 10^8^ |  | | 2-3d | |  | | |  | |  |  |  | |  | |  | |  | |  | |  | |  |
|  |  |  |  | DC1412[L] |  | 1.8 x 10^8^ |  | | 3d | |  | | |  | |  |  |  | |  | |  | |  | |  | |  | |  |
|  |  |  |  |  |  |  |  | |  | |  | | |  | |  |  |  | |  | |  | |  | |  | |  | |  |
| Italian VHSV strains | | | | | | | | | | | | | | | | | | | | | | | | | | | | | | |
| At the time of CPE | | | | | | | | | | | | |  | | Maximum viral production | | | | | | | | | | | | | | | |
| Line |  | MOI |  | Strain |  | Titer | |  | | Time | |  | | Line | |  | MOI |  | Strain | |  | | Titer | |  | | Time | |  |  |
| EPC |  | 0.1 |  | TN68[H] |  | 8.5 x 10^7^ | |  | | N/Av | |  | | EPC | |  | 0.1 |  | TN68[H] | |  | | 7.8 x 10^7^  *7.87±0.15* | |  | | 3d | |  |  |
|  |  |  |  | TN80[H] |  | NA | |  | |  | |  | |  | |  |  |  | TN80[H] | |  | | NA | |  | |  | |  |  |
|  |  |  |  | TN470[H] |  | 7.5 x 10^7^ | |  | | N/Av | |  | |  | |  |  |  | TN470[H] | |  | | 9.3 x 10^7^  *7.76±0.64* | |  | | 3d | |  |  |
|  |  |  |  | TN480[L] |  | 7.4 x 10^7^ | |  | | N/Av | |  | |  | |  |  |  | TN480[L] | |  | | 6.7 x 10^7^  *7.83±0.03* | |  | | 3d | |  |  |
|  |  |  |  |  |  |  | |  | |  | |  | |  | |  |  |  |  | |  | |  | |  | |  | |  |  |
| RTG-2 |  | 0.01 |  | TN68[H] |  | 1.1 x 10^8^ | |  | | 3-4d | |  | | RTG-2 | |  | 0.1 |  | TN68[H] | |  | | 2.0 x 10^7^  *7.28±0.15* | |  | | 7d | |  |  |
|  |  |  |  | TN80[H] |  | NA | |  | |  | |  | |  | |  |  |  | TN80[H] | |  | | 1.1 x 10^7^  *7.05±0.06* | |  | | 4d | |  |  |
|  |  |  |  | TN470[H] |  | 1.0 x 10^8^ | |  | | 3-4d | |  | |  | |  |  |  | TN470[H] | |  | | 3.4 x 10^7^  *7.52±0.14* | |  | | 3d | |  |  |
|  |  |  |  | TN480[L] |  | 4.1 x 10^7^ | |  | | 3-4d | |  | |  | |  |  |  | TN480[L] | |  | | 6.0 x 10^7^  *7.74±0.26* | |  | | 2.5d | |  |  |
|  |  |  |  |  |  |  | |  | |  | |  | |  | |  |  |  |  | |  | |  | |  | |  | |  |  |
|  |  | 0.1 |  | TN68[H] |  | 5.4 x 10^7^ | |  | | 2-3d | |  | |  | |  |  |  |  | |  | |  | |  | |  | |  |  |
|  |  |  |  | TN80[H] |  | NA | |  | |  | |  | |  | |  |  |  |  | |  | |  | |  | |  | |  |  |
|  |  |  |  | TN470[H] |  | 1.0 x 10^8^ | |  | | 3d | |  | |  | |  |  |  |  | |  | |  | |  | |  | |  |  |
|  |  |  |  | TN480[L] |  | 1.1 x 10^8^ | |  | | 2-3d | |  | |  | |  |  |  |  | |  | |  | |  | |  | |  |  |
|  |  |  |  |  |  |  | |  | |  | |  | |  | |  |  |  |  | |  | |  | |  | |  | |  |  |
|  |  | 1.0 |  | TN68[H] |  | 1.0 x 10^8^ | |  | | 2d | |  | |  | |  |  |  |  | |  | |  | |  | |  | |  |  |
|  |  |  |  | TN80[H] |  | NA | |  | |  | |  | |  | |  |  |  |  | |  | |  | |  | |  | |  |  |
|  |  |  |  | TN470[H] |  | 2.2 x 10^8^ | |  | | 2-3d | |  | |  | |  |  |  |  | |  | |  | |  | |  | |  |  |
|  |  |  |  | TN480[L] |  | 8.7 x 10^7^ | |  | | 2d | |  | |  | |  |  |  |  | |  | |  | |  | |  | |  |  |
|  |  |  |  |  |  |  | |  | |  | |  | |  | |  |  |  |  | |  | |  | |  | |  | |  |  |
| Danish VHSV strains | | | | | | | | | | | | | | | | | | | | | | | | | | | | | | |
| At the time of CPE | | | | | | | | | | | | |  | | Maximum viral production | | | | | | | | | | | | | | | |
| Line |  | MOI |  | Strain |  | Titer | |  | | Time | |  | | Line | |  | MOI |  | Strain | |  | | Titer | |  | | Time | |  |  |
| RTG-2 |  | 0.1 |  | DK3592[H] |  | 9.4 x 10^7^  *7.97±0.07* | |  | | 3d | |  | | RTG-2 | |  | 0.1 |  | DK3592[H] | |  | | 4.8 x 10^7^  *7.70±0.07* | |  | | 7.5d | |  |  |
|  |  |  |  | DK-F1[V] |  | 8.8 x 10^7^  *7.75±0.30* | |  | | 2-3d | |  | |  | |  |  |  | DK-F1[V] | |  | | 6.2 x 10^7^  *7.77±0.16* | |  | | 2d | |  |  |
|  |  |  |  | DK-1p8[L] |  | 7.7 x 10^6^  *6.18±0.25* | |  | | 3d | |  | |  | |  |  |  | DK-1p8[L] | |  | | 1.7 x 10^6^  *6.18±0.25* | |  | | 4d | |  |  |
|  |  |  |  |  |  |  | |  | |  | |  | |  | |  |  |  |  | |  | |  | |  | |  | |  |  |
| BF-2 |  | 0.1 |  | DK3592[H] |  | 1.2 x 10^8^ | |  | | 3d | |  | | BF-2 | |  | 0.1 |  | DK3592[H] | |  | | NDm | |  | |  | |  |  |
|  |  |  |  | DK-F1[V] |  | 2.3 x 10^6^ | |  | | 3d | |  | |  | |  |  |  | DK-F1[V] | |  | | NDm | |  | |  | |  |  |
|  |  |  |  | DK-1p8[L] |  | 2.3 x 10^6^ | |  | | 3d | |  | |  | |  |  |  | DK-1p8[L] | |  | | NDm | |  | |  | |  |  |
|  |  |  |  |  |  |  | |  | |  | |  | |  | |  |  |  |  | |  | |  | |  | |  | |  |  |
| French VHSV recombinant strains | | | | | | | | | | | | | | | | | | | | | | | | | | | | | | |
| At the time of CPE | | | | | | | | | | | | |  | | Maximum viral production | | | | | | | | | | | | | | | |
| Line |  | MOI |  | Strain |  | Titer | |  | | Time | |  | | Line | |  | MOI |  | Strain | |  | | Titer | |  | | Time | |  |  |
| EPC |  | 0.1 |  | Wt[H] |  | 7.5 x 10^8^ | |  | | 2-3d | |  | | EPC | |  | 0.1 |  | Wt[H] | |  | | 4.5 x 10^8^  *8.62±0.19* | |  | | 5d | |  |  |
|  |  |  |  | NV-R116Y[H] |  | 4.8 x 10^9^ | |  | | 2-3d | |  | |  | |  |  |  | NV-R116Y[H] | |  | | 3.6 x 10^8^  *8.52±0.21* | |  | | 5d | |  |  |
|  |  |  |  | NV-R116S[M] |  | 5.0 x 10^7^ | |  | | 2-3d | |  | |  | |  |  |  | NV-R116S[M] | |  | | 2.5 x 10^8^  *8.34±0.28* | |  | | 5d | |  |  |
|  |  |  |  | DD224[L] |  | 1.3 x 10^9^ | |  | | 2-3d | |  | |  | |  |  |  | DD224[L] | |  | | 2.9 x 10^8^  *8.47±0.06* | |  | | 5d | |  |  |
|  |  |  |  | N-K46G[L] |  | NA | |  | |  | |  | |  | |  |  |  | N-K46G[L] | |  | | 1.7 x 10^8^  *8.20±0.16* | |  | | 5d* | |  |  |
|  |  |  |  | NV_N[L] |  | NA | |  | |  | |  | |  | |  |  |  | NV_N[L] | |  | | 4.6 x 10^7^  *7.66±0.09* | |  | | 5d* | |  |  |

^1^CPE: Cytopathic Effect; ^2^Line: cell line tested; ^3^MOI: multiplicity of infection; ^4^Titer: TCID_50_/ml (below: Average±Standard Deviation, when 3 replicas were titrated); ND: Not Detected; NA: Not Assayed; NDm: Not Determined; N/Av: not available. *Considering the maximum time tested with the remaining strain (see Fig 10).
