# Supplementary material for: Steps of the Replication Cycle of the Viral Haemorrhagic Septicaemia Virus (VHSV) Affecting Its Virulence on Fish
Source: Animals (Basel). 2020 Dec 1;10(12):2264. doi: 10.3390/ani10122264 (PMC7761041; doi:10.3390/ani10122264)
Supplement: Supplementary file 1 [file animals-10-02264-s001.zip › Supplementary items-wo Fig Legend-2/Supplementary Table 4-Adsorption French strains-vs3.pdf]

Supplementary. Table 4.- Adsorption capacity of the French VHSV recombinant strains

Cell line: EPC

Adsorption time	Strain	Method	AAE[1]	RAE[2]	EOA[3]
Repetition 1: MOI 0.01-0.1					
30 min	Wt[H]	TCID	52.02±11.68	51.72±11.56	99.41±0.10
		qPCR	54.91±22.71	52.90±20.81	96.34±1.79
	DD224[L]	TCID	39.28±4.05	37.98±3.22	96.69±1.68
		qPCR	24.95±14.31	20.86±13.21	83.60±7.78
	NV-R116Y[H]	TCID	50.15±11.98	48.90±12.39	97.51±2.11
		qPCR	61.59±21.14	59.27±20.56	96.22±2.21
Repetition 2: MOI 0.1-1					
30 min	Wt[H]	TCID	72.99±7.99	72.60±7.99	99.46±0.06
		qPCR	95.26±2.25	91.94±3.63	96.51±2.46
	DD224[L]	TCID	50.27±24.79	49.77±24.92	99.01±0.99
		qPCR	27.93±21.42	26.34±21.62	94.32±8.48
	NV-R116Y[H]	TCID	70.99±28.50	70.89±28.54	99.82±.68
		qPCR	79.07±7.11	77.55±7.44	98.07±0.61
	NV-R116S[M]	TCID	55.07±19.57	54.97±19.62	99.83±0.14
		qPCR	24.25±8.36	22.44±7.78	92.54±0.71
Repetition 3: MOI 0.1-1					
30 min	Wt[H]	TCID	74.67±13.07	74.37±13.03	99.59±0.08
		qPCR	45.48±6.66	45.13±6.63	99.24±0.08
	DD224[L]	TCID	43.99±0.23	42.21±1.68	95.96±4.22
		qPCR	18.67±20.69	15.29±20.52	82.82±11.76
	NV_N[L]	TCID	73.61±14.90	73.28±14.95	99.54±0.21
		qPCR	50.94±9.49	49.21±9.39	96.61±2.51
	N-K46G[L]	TCID	84.81±4.49	84.62±4.32	99.77±0.19
		qPCR	49.52±23.24	46.57±22.84	94.06±3.73
Average from 3 repeats					
30 min	Wt[H]	TCID	66.56±10.91	66.23±10.86	99.49±0.08
		qPCR	65.22±10.54	63.32±10.36	97.36±1.44
	DD224[L]	TCID	44.51±9.69	43.32±9.94	97.22±2.30
		qPCR	23.85±18.81	20.83±18.85	86.91±9.34
	NV-R116Y[H]	TCID	60.57±20.24	59.90±20.47	98.67±1.40
		qPCR	70.33±14.13	68.41±14.00	97.15±1.41
	NV-R116S[M]	TCID	55.07±19.57	54.97±19.62	99.83±0.14
		qPCR	24.25±8.36	22.44±7.78	92.54±0.71
	NV_N[L]	TCID	73.61±14.90	73.28±14.95	99.54±0.21
		qPCR	50.94±9.49	49.21±9.39	96.61±2.51
	N-K46G[L]	TCID	84.81±4.49	84.62±4.32	99.77±0.19
		qPCR	49.52±23.24	46.57±22.84	94.06±3.73

[1]Apparent adsorption efficacy: AAE=TAV (total adsorbed virus)/TIV (total inoculated virus) × 100; [2]Real adsorption efficacy: RAE=IAV (irreversibly adsorbed virus)/TIV × 100; [3]Efficiency of adsorption: EOA=IAV/TAV × 100.
